# Direct evidence for poison use on microlithic arrowheads in Southern Africa at 60,000 years ago

**DOI:** 10.1126/sciadv.adz3281

**Published:** 2026-01-07

**Authors:** Sven Isaksson, Anders Högberg, Marlize Lombard

**Affiliations:** ^1^ Archaeological Research Laboratory, Stockholm University, Sweden.; ^2^ Department of Cultural Sciences, Linnaeus University, Sweden.; ^3^ Palaeo-Research Institute, University of Johannesburg, South Africa.

## Abstract

Poisoned weapons are a hallmark of advanced hunter-gatherer technology. Through targeted microchemical and biomolecular analyses, we identified traces of toxic plant alkaloids on backed microliths from Umhlatuzana Rock Shelter in KwaZulu-Natal, South Africa, excavated from a level dated to 60,000 years ago. The alkaloids buphandrine and epibuphanisine only originate from Amaryllidaceae indigenous to southern Africa. The most likely source is *Boophone disticha* (L.f.) Herb. bulb exudate, also associated with historically documented arrow poisons. To our knowledge, we present the first direct evidence for the application of this plant-based poison on the tips of Pleistocene hunting weapons. The discovery highlights the complexity of subsistence strategies and cognition in southern Africa since the mid-Pleistocene.

## Teaser

Earliest proof of plant poisons on arrows reveals complex Pleistocene hunting in southern Africa.

## INTRODUCTION

Human adaptation to meat consumption brought several evolutionary advantages, including resistance to certain diseases, enhanced life expectancy, and neuronal growth (*1*). These benefits likely contributed to the development of weapon-assisted hunting techniques. Evidence of bowhunting in Pleistocene southern Africa includes bone arrowheads dated to approximately 70 to 60 thousand years (ka) that closely resemble in shape and size Holocene examples of poisoned bone arrowheads (*2*). Some ancient bone arrowheads also show use wear and residue distribution patterns similar to those observed on arrow tips used during the last century (*3*). The use of poisoned hunting weapons represents a remarkable innovation in meat acquisition strategies and has intrigued researchers for centuries (*4*), with recent neurocognitive work highlighting how the technology may contribute to our understanding of the development of technical and cognitive complexity (*5*).

**Fig. 1.**
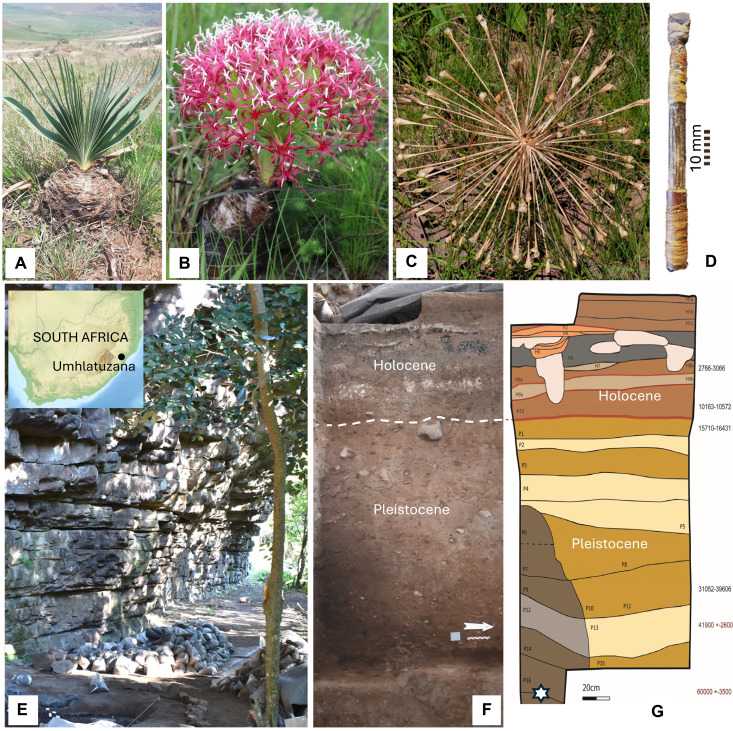
*Boophone disticha* (L.f.) Herb*.*, microlithic arrowhead, and Umhlatuzana Rock Shelter. (**A**) *B. disticha* bulb and fan-like leaves (photo credit: A. Motala, CC BY-SA, https://creativecommons.org/licenses/by-nc-sa/4.0/deed.en), (**B**) flowerhead (photo credit: G. Bowers-Winters, CC BY-NC, https://creativecommons.org/licenses/by-nc/4.0/deed.en), and (**C**) seeding structure (photo credit: R. Taylor, CC BY-NC, https://creativecommons.org/licenses/by-nc/4.0/deed.en). (**D**) An approximately 2000-year-old arrowhead from South Africa with transversely hafted microlith [(*11*); photo credit: by M.L.]. (**E**) Umhlatuzana Rock Shelter, with (**F**) the western profile exposed in 2018–2019, and (**G**) simplified stratigraphic drawing with ages in BP, the star indicating the layer from which the backed microliths were excavated [images adapted from (*53*), an open-source paper under a Creative Commons Attribution 4.0 International License (http://creativecommons.org/licenses/by/4.0/), with permission from G. Dusseldorp and I. Sifogeorgaki].

Early analyses of poison residues indicate that some components are resilient. For instance, poison containing diamphotoxin on arrows from Namibia was found to be pharmacologically active after 80 years (*6*). Similarly, *Boophone disticha* (L.f.) Herb. (Fig. 1, A to C) bulb poison on arrowheads made by hunter-gatherers in present-day South Africa still contained active components after 100 years (*7*). More recently, residues of plant derived cardiotonic glycosides were detected on a 1000-year-old archaeological bone arrow tip (*8*).

Before the present study, the earliest direct evidence of poisoned arrows dated to the mid-Holocene. Bone-tipped arrows containing toxic glycoside residues were found in an Egyptian tomb dating to 4431 to 4000 years before the present (BP) (*9*), and cardiac glycosides were identified on bone arrow points from Kruger Cave in South Africa, dating to approximately 6700 BP (*10*). Pleistocene evidence of poison traces was previously limited to two finds: an approximately 24 ka “poison applicator” found at Border Cave in KwaZulu-Natal, South Africa [although source-plant identification remains debated (*10*)], and in an approximately 35 ka lump of beeswax from the same site (*11*).

Alongside bone points, hunters used backed microliths as arrow tips throughout the African Holocene (*9*, *12*–*14*) (Fig. 1D). On the basis of use-traces and residue distribution patterns (*15*), as well as replication experiments and fracture studies (*16*), similar artifacts have been interpreted as tips and barbs for arrows dating to >60 ka. Some researchers (*17*–*19*) hypothesized that these small stone artifacts were used with poison. At Umhlatuzana Rock Shelter in KwaZulu-Natal (Fig. 1E), backed microliths recovered from the same general stratigraphic position dated by optically stimulated luminescence to 60 ± 3.5 ka [(*20*); Fig. 1, F and G], exhibit macrofractures consistent with projectile use (*21*) and residue traces consistent with complex adhesives containing resin or water-soluble gum mixed with ochre (*22*). Most of these artifacts are also morphologically best suited for use as tips on poisoned arrows (*23*).

Recent geochemical analyses of the site’s sediments (*24*) confirmed a gradual and steady depositional history, with no evidence for large-scale depositional mixing. Although single quartz grains show some evidence of percolation, the majority at each level remained in primary context, so that accurate ages could be estimated for the associated artifacts from the host quartz-grain populations (*20*). A magnetic analysis showed that, although seemingly homogenous, distinctive lateral and vertical variations between the layers are present in the Pleistocene deposits. The Pleistocene sediments are geogenic with distinct anthropogenic input and traces of biogenic materials (excrement), with preservation driven by the variation in pH, which shows acidic values for the Pleistocene and alkaline conditions for the Holocene deposits (*24*) (Fig. 1, F and G). Small-scale sediment mixing related to bioturbation is evidenced in the micromorphological thin sections. It did not prevent the recording of horizontal microlayering in both the Pleistocene and Holocene contexts and does not seem to affect the vertical distribution of the artifacts (*25*) suggested by Kaplan (*26*). These stratigraphic and sedimentological studies confirm that although minor bioturbation occurred, the Pleistocene layers at Umhlatuzana Rock Shelter remained sufficiently intact to allow the attribution of artifacts to coherent assemblages (*24*).

What is more, Sifogeorgaki and Dusseldorp (*27*) found that certain sedimentary contexts, such as Kaplan’s (*26*) RBS (red brown sand) deposits, within the site’s deposit were less exposed to moisture than others, which may be conducive of some organic preservation. The previous functional observations that backed microliths from Umhlatuzana Rock Shelter may have functioned as arrow tips (*21*, *22*), together with the new geoarchaeological results (*24*, *25*), prompted us to analyze 10 quartz backed pieces with macroscopically visible residues from the 60 ka context to test for microchemical and biomolecular poison traces (see Materials and Methods and Supplementary Text).

## RESULTS

Gas chromatography–mass spectrometry (GC-MS) analysis revealed the presence of plant derived toxic alkaloids on five of the 10 analyzed quartz microliths (Table 1). Buphanidrine (1,2-didehydro-3α,7-dimethoxycrinan) was detected in samples 001 to 005, and epibuphanisine (1,2-didehydro-3α-methoxycrinan) in sample 001 (data S1 to S10). The compounds were also detected on four 250-year-old poisoned bone arrowheads in the ethnohistorical comparative material (fig. S2 and data S11 to S18). While structurally related alkaloids, such as these crinane-type compounds, can produce similar mass spectra, the main components we identified are well-characterized and produce a distinct EI mass spectra in both the ethnohistorical and the archaeological samples. Both alkaloids are found in several southern African Amaryllidaceae species (table S2), but only *B. disticha* (Fig. 1, A to C) bulb exudate is well recorded as an arrow poison (table S3). To authenticate the detection of these alkaloids, we analyzed extracts of modern *B. disticha* bulb exudate, using the same procedure as for the archaeological samples, confirming the detection of buphanidrine (1,2-didehydro-3α,7-dimethoxycrinan) and epibuphanisine (1,2-didehydro-3α-methoxycrinan) (data S19). The chemical stability of buphanidrine, due to its moderate lipophilicity and low water solubility, its structural rigidity and lack of hydrogen bond donors, contributed to its preservation on the Umhlatuzana Rock Shelter microliths. A detailed account of the chemical and physical properties of buphanidrine and epibuphanisine is available in Supplementary Text.

**Table 1.** Result summary of the microchemical and biomolecular analysis of organic residues on backed microliths from Umhlatuzana Rock Shelter, KwaZulu-Natal, dated to 60 ka, and four ethnohistorical poisoned bone arrowheads collected in South Africa sometime during 1772–1774. (sample numbers = artifact numbers, fig. S1; square = excavated square for points sampled; L = length, W = width, and T = thickness of quartz microliths). For each identified compound in each sample, the match score given by the NIST Mass Spectral Search Program 2.3 software to the mass spectrum recorded in the extract from the sample in comparison to the reference mass spectrum in the NIST 2017 MS database is given. Match score <600 = poor match, 600 to 700 = between poor and fair match, 700 to 800 = fair match, 800 to 900 = good match, and >900 = excellent match.

Sample (square)	L/W/T in mm	Epibuphanisine	Buphanidrine	Myristic acid	Palmitic acid	Stearic acid	Oleic acid	Dehydro-abietic acid
001 (J2)	14/9/2	729	873		876	819		598
002 (J2)	19/11/2		810	816	897	838	761	
003 (J2)	13/7/3		682		861			
004 (J2)	19/11/3		683		839	751		
005 (J2)	15/7/3		729		875	797		
006 (J2)	13/8/2				875	814		
007 (J2)	10/5/2				882			
008 (K2)	15/9/4				877	849		
009 (K2)	17/8/2				863	817		
010 (K2)	15/11/3				834			
1799.02.75.k	NA	893	897		783		825	
1799.02.75.m	NA	869	842		796		865	
1874.01.251.c	NA	889	902		775			
1874.01.251.d	NA	895	900		865			

**Fig. 2.**
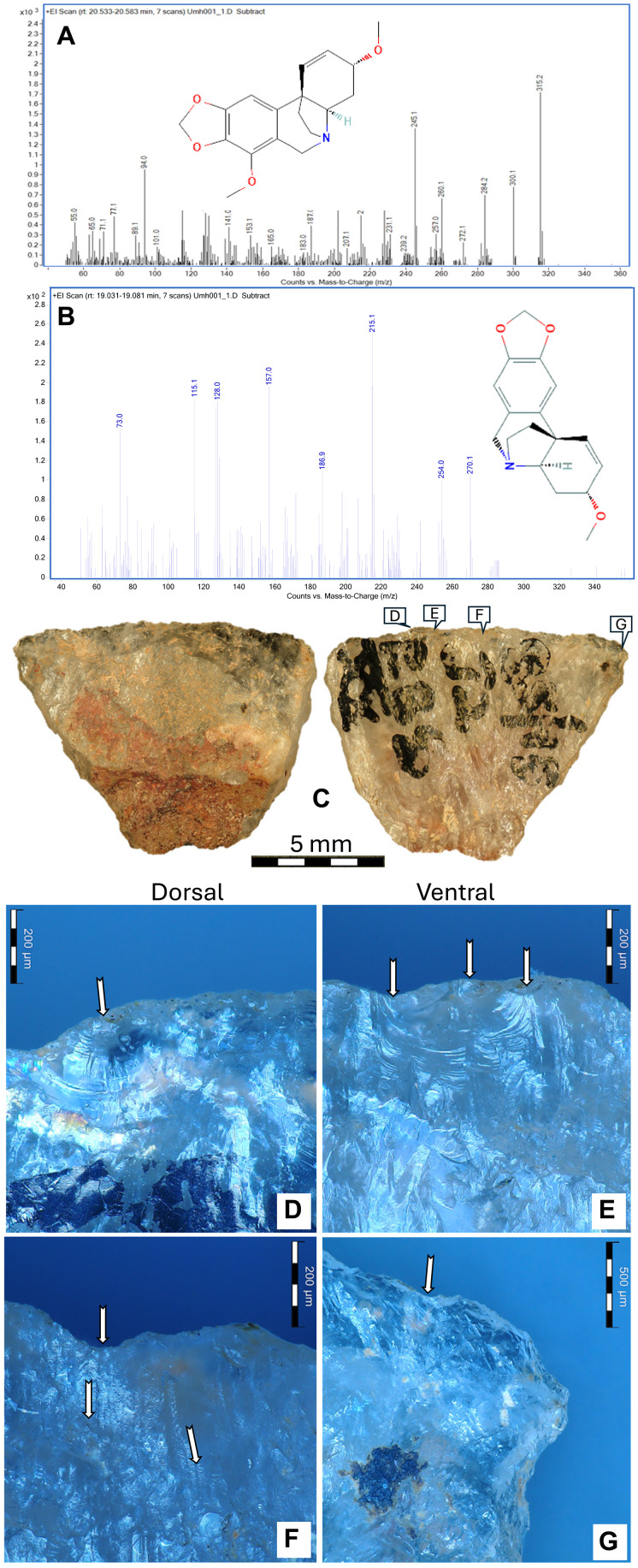
Umhlatuzana Rock Shelter backed microlith 001. (**A**) The buphanidrine and (**B**) epibuphanisine detected in sample 001. (**C**) Microlith 001 showing the reddish poisonous adhesive residue still adhering to the dorsal backed portion of the tool, D-G on the ventral edge correspond to the micrographs below. (**D** and **E**) Microscopic impact scars along the sharp ventral edge, and (**F**) transverse microstriations initiating from the sharp ventral edge. (**G**) Impact scars on the transition between the sharp ventral edge and one of the lateral sides [for comparison with experimental transverse arrow-tip use, see (*16*, *28*, *29*)].

On artifact 001, the residue was macroscopically visible along the dorsal backed portion, suggesting that the alkaloids were likely part of the adhesive (Fig. 2, A to C). The residue distribution pattern indicates that the microlith was hafted transversely, similar to the 2000-year-old arrow from Adams Kranz, Eastern Cape, South Africa [(*12*); Fig. 1D]. Impact scarring and microstriations along the sharp ventral edge of the microlith suggest transverse impact (Fig. 2, D to G), consistent with its use as a transversely hafted arrow tip (*16*, *28*, *29*).

Fatty acids were also detected, dominated by palmitic acid. One sample contained traces of monounsaturated oleic acid (Table 1). Since unsaturated fatty acids decompose easily its presence in these very old samples may point toward a more recent origin for these lipid residues, deriving from, e.g., human fingerprints due to handling. However, other common components of human skin lipids, such as cholesterol and squalene, are not present in the samples. Traces of unsaturated fatty acids have been reported before in ancient organic residues from southern Africa (*11*, *30*). The palmitic-to-stearic acid (*P*/*S*) ratios (2.5 to 5.5) are consistent with a plant lipid origin (*31*, *32*).

## DISCUSSION

Archaeobotanical remains of *B. disticha* have been recovered from South African Holocene archaeological contexts (approximately 4000 to 500 BP), where the less toxic leaves or outer bulb scales were used as preservatives (*33*–*35*). The plant, known locally as *gifbol* (poison bulb) (Fig. 1, A to C), is widely recognized as a source for arrow poison in southern Africa where it grows abundantly [(*4*); tables S2 and S3]. It was also the first arrow-poison from the region to be identified chemically in a residue (*7*).

Carl Peter Thunberg, an early European traveler (1772–1774) into the hinterland of the Cape of Good Hope, who collected plant specimens and indigenous knowledge about their uses for Carl Linnaeus, wrote of *B. disticha* (named Thunberg *Amaryllis disticha* and by Linneaus *Buphane toxicaria* Thunberg) that the fist-sized root of the plant is poisonous, and that indigenous hunters used it mainly for poisoning their arrows to hunt game such as springbok (*Antidorcas marsupialis*) (*36*). Two of the arrows probably collected by him (fig. S2), included in our ethnohistorical control, also tested positive for buphanidrine and epibuphanisine (data S13 and S14), consistent with *B. disticha* bulb exudate (*37*)*. B. disticha* bulb extracts contain several toxic alkaloids (*37*), but buphanidrine stands out (data S19), and are notably resistant to decomposition (see discussion in Supplementary Text). While other Amaryllidaceae species also contain these alkaloids (table S2), only *B. disticha* bulb exudate has repeated and authenticated use in arrow poisons. The Ju/‘uan San of the Kalahari, however, uses *Ammocharis coranica* (Ker Gawl.) Herb. bulb scales, which may contain epibuphanisine but not buphanidrine (table S2), heated and beaten into an elastic pulp, as adhesive for making arrows (*38*).

Finding buphanidrine on five of the 10 sampled backed microliths, with morpho-ballistic traits similar to Holocene arrow tips presumed to have been used with poison (*23*), cannot be coincidental. If *B. disticha* leaves were used as preservative at the site, then buphanidrine and epibuphanisine from bulb exudates (tables S2 and S3), would not be present on the microliths. Ancient hunter-gatherers would have been familiar with the toxic properties of the bulb exudates, in the same way they understood the insecticidal and larvicidal properties of some aromatic leaves in KwaZulu-Natal by approximately 77 ka (*39*), so that the substance would probably not have been ubiquitous in their living space. The specificity of these compounds also excludes postexcavation contamination (see Supplementary Text for further curatorial detail).

Although there are no direct archaeobotanical or palaeobotanical data to show that *B. disticha* grew near Umhlatuzana Rock Shelter during MIS 4 (approximately 71 to 57 ka), the plant is indigenous to South Africa and KwaZulu-Natal and has a molecular philogyny that suggests an age of 20 to 10 million years ago (*40*) (Supplementary Text). The common habitat of *B. disticha* is grassland, savanna, and Karoo vegetation (*41*), with a grassland-savanna mosaic ecology surrounding Umhlatuzana Rock Shelter, and bulbs can survive for a century or more (*42*), despite drought cycles and fire regimes (*43*). The plant is therefore widespread throughout the southern, eastern and northern regions of South Africa (table S2). Its long evolutionary history in the region, its hardiness, and the absence of ice covers in southern Africa since the Paleozoic Ice Ages [370 to 260 million years ago (*44*)] suggest that *B. disticha* was available to the mid-Pleistocene inhabitants of the site.

The milky *B. disticha* bulb exudate can be used on its own as a minimally processed arrow poison. For example, after the thick liquid is sundried into a gum-like consistency it is ready for application (*45*). It can also be reduced by cooking or heating over a fire and be mixed with other plant- or animal-based ingredients (table S3). Toxicological tests of a San arrow treated with *B. disticha* show that even small quantities of the exudate can be lethal to rodents within 20 to 30 min (*46*). In humans, symptoms include nausea, coma, muscular flaccidity, visual impairment, stertorous breathing, respiratory paralysis, feeble or increased pulse, dyspnea, and hyperemia and edema of the lungs (*37*). Its efficacy as a hunting poison depends on preparation and dosage, and in lower doses it may have medicinal application (*4*, *37*).

When considering arrow-poison toxicity, it is important to understand that Holocene poisoned arrows from southern Africa are/were not designed to kill instantly, on impact or through lethal penetration depth. Instead, they are small, light-weight artifacts (*23*), adapted to carry poison (*38*), which is delivered into an animal’s bloodstream through a small wound pierced by an arrowhead engineered to break loose from its shaft and remain under the animal’s skin. Wounded animals usually continue to run for several kilometers during which hunters will track them, sometimes for a day or more (*45*, *47*). We cannot know to which extent Pleistocene stone-tipped or stone-barbed arrows were engineered in the same way. The quartz microliths from Umhlatuzana Rock Shelter are, however, similar in size and shape compared to those from the Holocene (*12*, *22*), and by using similar poisons as Holocene bowhunters, hunting weapons and strategies may not have differed much.

Although the mid-Pleistocene hunters from Umhlatuzana Rock Shelter lacked formal chemical knowledge, our study demonstrates that they had a knowledge system or procedural knowledge (*48*) enabling them to identify, extract and apply toxic plant exudates effectively. They must have also had the necessary understanding of prey ecology and behavior (ethology) to know that if shot into a prey animal, the delayed effect of poison would cause it to weaken after some time, contributing to the efficiency of attrition or persistence hunting. Such out-of-sight, long-distance action is a convincing proxy for complex cognition that requires response inhibition enabled through enhanced working memory (*49*). Because poison is not a physical force, but functions chemically, the hunters must also have relied on advanced planning, abstraction and causal reasoning (*50*). Thus, apart from providing the first evidence of hunting with poisoned arrows during the late-Pleistocene in southern Africa, our findings contribute to the understanding of human adaptation and technobehavioral complexity during a phase of rapid, cumulative innovation in the region (*5**,* *47**,* *51**,* *52*).

## MATERIALS AND METHODS

We sampled 10 quartz backed pieces (artifact numbers = sample numbers; fig. S1) from Layer RBS XX associated with the Howiesons Poort microlithic technocomplex at Umhlatuzana Rock Shelter (*26*) (Fig. 1G), and with dryer Pleistocene sediments (*27*), which may be conducive of slightly better preservation conditions compared to the wetter phosphate-buffered saline layers. This represents a minimally destructive sampling strategy [10 of 216 artifacts; 649 total backed pieces reported (*26*)], preserving materials for future research. The remarkable chemical diversity of naturally occurring products made even more complex in potentially degraded, archaeological samples is analytically challenging. However, this same chemical diversity also results in a wide range of mass spectral signatures, which can aid in distinguishing between different compounds. Small amounts (<100 μg) of residues, previously interpreted as adhesives based on optical microscopy (*22*), were microscopically collected (away from any traces of recent handling), dissolved via a cradled microchemical technique, and derivatized before GC-MS analysis. This analytical technique allows us to identify components that are already well-characterized and with specific electron ionization mass spectra such as buphanidrine and epibuphanisine. For comparative purposes, we also analyzed ethnohistorical arrow poison from four arrows [two of which were collected by Thunberg who observed the use of *B. disticha* arrow poison in South Africa during the late 1700s (*36*)] (fig. S2), and bulb exudes from modern *B. disticha* (data S19), obtaining similar GC-MS results as for the 60 ka quartz microliths from Umhlatuzana Rock Shelter. Such authentic comparative material and results provide strong contextual and chemical support for the identification of components in the archaeological samples. Detailed protocols and further information are available in the Supplementary Materials.

## Supplementary Materials

### This PDF file includes:

Supplementary Text Figs. S1 to S6 Tables S1 to S3 Data S1 to S19 References

## References

[1] C. E. Finch, C. B. Stanford, Meat-adaptive genes and the evolution of slower aging in humans. Q. Rev. Biol. 79, 3–50 (2004). 15101252 10.1086/381662

[2] L. Backwell, J. Bradfield, K. J. Carlson, T. Jashashvili, L. Wadley, F. d’Errico, The antiquity of bow-and-arrow technology: Evidence from Middle Stone Age layers at Sibudu Cave. Antiquity 92, 289–303 (2018).

[3] J. Bradfield, M. Lombard, J. Reynard, A. Wurz, Further evidence for bow hunting and its implications more than 60 000 years ago: Results of a use-trace analysis of the bone point from Klasies River Main site, South Africa. Quat. Sci. Rev. 236, 106295 (2020).

[4] H.D. Neuwinger, *African ethnobotany: Poisons and drugs: Chemistry*, *pharmacology*, *toxicology* (CRC Press, 1996).

[5] M. Lombard, From complex techno-behaviour to complex attention through the genes of the precuneus. J. Archaeol. Method Theory 32, e46 (2025).

[6] M. Shaw, P. Woolley, F. Rae, Bushmen arrow poisons. Cimbebasia 7, 2–41 (1963).

[7] L. Lewin, Untersuchungen über Buphane disticha (*Haemanthus toxicarius*). Archiv f. experiment. Pathol. u. Pharmakol. 68, 333–340 (1912).

[8] S. Isaksson, A. Högberg, M. Lombard, J. Bradfield, Potential biomarkers for southern African hunter-gatherer arrow poisons applied to ethno-historical and archaeological samples. Sci. Rep. 13, 11877 (2023). 37482542 10.1038/s41598-023-38735-0 PMC10363533

[9] J. D. Clark, J. L. Phillips, P. S. Staley, Interpretations of prehistoric technology from ancient Egyptian and other Sources. Part I: Ancient Egyptian bows and arrows and their relevance for African prehistory. *Paléorient* 2, 323–388 (1974).

[10] J. Bradfield, I. A. Dubery, P. A. Steenkamp, A 7,000-year-old multi-component arrow poison from Kruger Cave, South Africa. iScience 27, 111438 (2024). 39759027 10.1016/j.isci.2024.111438 PMC11699280

[11] F. d’Errico, L. Backwell, P. Villa, I. Degano, J. J. Lucejko, M. K. Bamford, T. F. G. Higham, M. P. Colombini, P. B. Beaumont, Early evidence of San material culture represented by organic artifacts from Border Cave, South Africa. Proc. Natl. Acad. Sci. U.S.A. 109, 13214–13219 (2012). 22847420 10.1073/pnas.1204213109 PMC3421171

[12] J. N. F. Binneman, A unique stone-tipped arrowhead from Adam’s Kranz Cave, Eastern Cape. South. Afr. field archaeol. 3, 58–60 (1994).

[13] M. M. Lahr, F. Rivera, R. K. Power, A. Mounier, B. Copsey, F. Crivellaro, J. E. Edung, J. M. Fernandez, C. Kiarie, J. Lawrence, A. Leakey, E. Mbua, H. Miller, A. Muigai, D. M. Mukhongo, A. van Baelen, R. Wood, J.-L. Schwenninger, R. Grün, H. Achyuthan, A. Wilshaw, R. A. Foley, Inter-group violence among early Holocene hunter-gatherers of West Turkana, Kenya. Nature 529, 394–398 (2016). 26791728 10.1038/nature16477

[14] S. T. Goldstein, C. M. Shaffer, Experimental and archaeological investigations of backed microlith function among mid-to-late Holocene herders in southwestern Kenya. Archaeol. Anthropol. Sci. 9, 1767–1788 (2017).

[15] M. Lombard, Quartz-tipped arrows older than 60 ka: Further use-trace evidence from Sibudu, KwaZulu-Natal, South Africa. J. Archaeol. Sci. 38, 1918–1930 (2011).

[16] P. de la Peña, N. Taipale, L. Wadley, V. Rots, A techno-functional perspective on quartz micro-notches in Sibudu’s Howiesons Poort indicates the use of barbs in hunting technology. J. Archaeol. Sci. 93, 166–195 (2018).

[17] D. W. Phillipson, Some speculations on the beginning of backed-microlith manufacture, in *Proceedings of the Eighth Pan-African Congress of Prehistory and Quaternary Studies*. B. A. Ogot, R. E. Leakey, Eds., 229–230. (Nairobi: The International Louis Leakey Memorial Institute for Prehistory and Paleontology, 1980).

[18] S. H. Ambrose, Small things remembered: Origins of early microlithic industries in sub-Saharan Africa. Archeol. Pap. Am. Anthropol. Assoc. 12, 9–29 (2002).

[19] J. Pargeter, J. J. Shea, Going big versus going small: Lithic miniaturization in hominin lithic technology. Evol. Anthropol. Issues News Rev. 28, 72–85 (2019). 10.1002/evan.21775 30924224

[20] M. Lombard, L. Wadley, Z. Jacobs, M. Mohapi, R. G. Roberts, Still Bay and serrated points from Umhlatuzana rock shelter, KwaZulu-Natal, South Africa. J. Archaeol. Sci. 37, 1773–1784 (2010).

[21] M. Lombard, L. Phillipson, Indications of bow and stone-tipped arrow use 64 000 years ago in KwaZulu-Natal, South Africa. Antiquity 84, 635–648 (2010).

[22] M. Lombard, The gripping nature of ochre: The association of ochre with Howiesons Poort adhesives and Later Stone Age mastics from South Africa. J. Hum. Evol. 53, 406–419 (2007). 17643475 10.1016/j.jhevol.2007.05.004

[23] M. Lombard, Testing for poisoned arrows in the Middle Stone Age: A tip cross-sectional analysis of backed microliths from southern Africa. J. Archaeol. Sci. Rep. 34, 102630 (2020).

[24] F. H. Reidsma, I. Sifogeorgaki, A. Dinckal, H. Huisman, M. J. Sier, B. van Os, G. L. Dusseldorp, Making the invisible stratigraphy visible: A grid-based, multi-proxy geoarchaeological study of Umhlatuzana Rockshelter, South Africa. Front. Earth Sci. 9, 664105 (2021).

[25] I. Sifogeorgaki, H. Huisman, P. Karkanas, V. C. Schmid, G. L. Dusseldorp, Sand, hearths, lithics and a bit of bioturbation: Site formation processes at Umhlatuzana rockshelter, South Africa. Geoarchaeology 39, 212–237 (2024).

[26] J. Kaplan, The Umhlatuzana rock shelter sequence: 100 000 years of Stone Age history. South. Afr. Humanit. 2, 1–94 (1990).

[27] I. Sifogeorgaki, G. L. Dusseldorp, True colours: Analysing apparent sedimentary divisions in the stratigraphic sequence of Umhlatuzana Rockshelter (South Africa) using Munsell-based colour determinations. South. Afr. field archaeol. 17, e1329 (2023).

[28] A. Fischer, P. V. Hansen, P. Rasmussen, Macro and micro wear traces on lithic projectile points. J. Dan. Archaeol. 3, 19–46 (1984).

[29] A. Yaroshevich, D. Kaufman, D. Nuzhnyy, O. Bar-Yosef, M. Weinstein-Evron, Design and performance of microlith implemented projectiles during the Middle and the Late Epipaleolithic of the Levant: Experimental and archaeological evidence. J. Archaeol. Sci. 37, 368–388 (2010).

[30] P. Villa, L. L. Pollarolo, I. Degano, L. Birolo, M. Pasero, C. Biagioni, K. Douka, R. Vinciguerra, J. J. Lucejko, L. Wadley, A milk and ochre paint mixture used 49,000 years ago at Sibudu, South Africa. PLOS ONE 10, e0131273 (2015). 26125562 10.1371/journal.pone.0131273 PMC4488428

[31] A. Casoli, P. C. Musini, G. Palla, Gas chromatographic-mass spectrometric approach to the problem of characterizing binding media in paintings. J. Chromatogr. A 731, 237–246 (1996).

[32] R. Chasan, M.-A. Veall, L. I. Baron, A. Aleo, P. R. B. Kozowyk, G. H. J. Langejans, Podocarpaceae and Cupressaceae: A tale of two conifers and ancient adhesives production in South Africa. PLOS ONE 19, e0306402 (2024). 39536024 10.1371/journal.pone.0306402 PMC11560044

[33] H. J. Deacon, Excavations at Boomplaas Cave: A sequence through the upper Pleistocene and Holocene in South Africa. World Archaeol. 10, 241–257 (1979).

[34] M. Steyn, J. Binneman, M. Loots, The Kouga mummified human remains. S. Afr. Archaeol. Bull. 62, 3–8 (2007).

[35] J. Bradfield, S. Woodborne, J. Hollmann, I. Dubery, A 500-year-old medicine container discovered near Misgund, Eastern Cape, South Africa: Residue characterisation by GC-MS. S. Afr. J. Sci. 119, 1–8 (2023).

[36] C. P. Thunberg, *Travels in Europe*, *Africa and Asia made between the years 1772 and 1779*, *Volume II: Containing two expeditions to the interior part of the country adjacent to the Cape of Good Hope and a voyage to the island of Java*, *performed in the years 1773*, *1774*, *and 1775*. Second Edition. (London: Printed for F. & C. Rivington and sold by W. Richardson, 1795).

[37] J. J. Nair, J. Van Staden, Traditional usage, phytochemistry and pharmacology of the South African medicinal plant *Boophone disticha* (L.f.) Herb. (Amaryllidaceae). J. Ethnopharmacol. 151, 12–26 (2014). 24211396 10.1016/j.jep.2013.10.053

[38] L. Wadley, G. Trower, L. Backwell, F. d’Errico, Traditional glue, adhesive and poison used for composite weapons by Ju/hoan San in Nyae Nyae, Namibia. Implications for the evolution of hunting equipment in prehistory. PLOS ONE 10, e0140269 (2015). 26509730 10.1371/journal.pone.0140269 PMC4625037

[39] L. Wadley, C. Sievers, M. Bamford, P. Goldberg, F. Berna, C. Miller, Middle Stone Age bedding construction and settlement patterns at Sibudu, South Africa. Science 334, 1388–1391 (2011). 22158814 10.1126/science.1213317

[40] L. Costa, H. Jimenez, R. Carvalho, J. Carvalho-Sobrinho, I. Escobar, G. Souza, Divide to conquer: Evolutionary history of Allioideae tribes (Amaryllidaceae) is linked to distinct trends of karyotype evolution. Front. Plant Sci. 11, e320 (2020). 10.3389/fpls.2020.00320 PMC7155398 32318079

[41] E. Van Jaarsveld, *Boophone* Amaryllidaceae, in *Monocotyledons*, *Illustrated Handbook of Succulent Plants*, U. Eggli, R. Nyffeler, Eds., (Berlin, Heidelberg, Springer, 2020).

[42] P. Xaba, G. Duncan, *Boophone disticha*, the century plant. Veld & Flora 94, 38–40 (2008).

[43] G. Williamson, The remarkable *Boophone disticha* (L.f.) Herb. (Amaryllidaceae); Survivor from the northern tropical savanna woodlands to the South African semi-arid Karoo. Cact. Succ. J. 84, 88–91 (2012).

[44] P. Dietrich, F. Guillocheau, G. A. Douillet, N. P. Griffis, G. Baby, D. P. Le Héron, L. Barrier, M. Mathian, I. P. Montañez, C. Robin, T. Gyomlai, C. Kettler, A. Hofmann, The Glacial Paleolandscapes of Southern Africa: The legacy of the late Paleozoic ice age. EGUsphere, 10.5194/egusphere-2024-467. hal-04675295 (2024).

[45] W. Paterson, *Quatre voyages chez les Hottentots et chez les Cafres*, *Depuis mai 1777 jusqu’en décembre 1779*. (Didot, 1790). [Four Journeys to the Hottentots and the Caffres, From May 1777 to December 1779].

[46] D. Mebs, J. Rohrich, G. Kauert, P. R. Becker, Pfeilgifte: Eine toxokologische Spurensuche im Mumeum. Dtsch. Apoth. Ztg. 136, 24–27 (1996). [Arrow Poisons: A Toxicological Investigation in the Museum].

[47] L. Wadley, What stimulated rapid, cumulative innovation after 100,000 years ago? J. Archaeol. Method Theory 28, 120–141 (2021).

[48] T. Wynn, F. L. Coolidge, A Stone-Age meeting of minds: Neandertals became extinct while *Homo sapiens* prospered. A marked contrast in mental capacities may account for these different fates. Am. Sci. 96, 44–51 (2008).

[49] L. Wadley, Were snares and traps used in the Middle Stone Age and does it matter? A review and a case study from Sibudu, South Africa. J. Hum. Evol. 58, 179–192 (2010). 20031191 10.1016/j.jhevol.2009.10.004

[50] P. Gärdenfors, M. Lombard, Causal cognition, force dynamics and early hunting technologies. Front. Psychol. 9, 87 (2018). 29483885 10.3389/fpsyg.2018.00087 PMC5816055

[51] G. B. Silberbauer, *Hunter and habitat in the central Kalahari Desert*. (Cambridge Univ. Press, 1981).

[52] T. Rito, D. Vieira, M. Silva, E. Conde-Sousa, L. Pereira, P. Mellars, M. B. Richards, P. Soares, A dispersal of *Homo sapiens* from southern to eastern Africa immediately preceded the out-of-Africa migration. Sci. Rep. 9, 4728 (2019). 30894612 10.1038/s41598-019-41176-3 PMC6426877

[53] I. Sifogeorgaki, V. Klinkenberg, I. Esteban, M. Murungi, A. S. Carr, V. B. van den Brink, G. L. Dusseldorp, New Excavations at Umhlatuzana Rockshelter, KwaZulu-Natal, South Africa: A Stratigraphic and taphonomic evaluation. Afr. Archaeol Rev. 37, 551–578 (2020).

[54] A. Sparrman, *A Voyage to the Cape of Good Hope*, *Towards the Antarctic Polar Circle*, *and Round the World: But Chiefly Into the Country of the Hottentots and Caffres*, *from the year 1772*, *to 1776* (Vol. 1). G. G. J. and J. Robinson, Pater-noster-row: London, (1786).

[55] V. Papakosta, R. H. Smittenberg, K. Gibbs, P. Jordan, S. Isaksson, Extraction and derivatization of absorbed lipid residues from very small and very old samples of ceramic potsherds for molecular analysis by gas chromatography–mass spectrometry (GC–MS) and single compound stable carbon isotope analysis by gas chromatography–combustion–isotope ratio mass spectrometry (GC–C–IRMS). Microchem. J. 123, 196–200 (2015).

[56] L. Scott, H. M. Anderson, J. M. Anderson, R. M. Cowling, D. M. Richardson, S. M. Pierce, Vegetation history. Vegetation of southern Africa, 62–84 (1997).

[57] G. J. Bredenkamp, F. Spada, E. Kazmierczak, On the origin of northern and southern hemisphere grasslands. Plant Ecol. 163, 209–229 (2002).

[58] M. E. Bianconi, J. Hackel, M. S. Vorontsova, A. Alberti, W. Arthan, S. V. Burke, M. R. Duvall, E. A. Kellogg, S. Lavergne, M. R. McKain, A. Meunier, C. P. Osborne, P. Traiperm, P.-A. Christin, G. Besnard, Continued adaptation of C4 photosynthesis after an initial burst of changes in the Andropogoneae grasses. Syst. Biol. 69, 445–461 (2020). 31589325 10.1093/sysbio/syz066 PMC7672695

[59] C. A. Smith, *Common names of South African plants* (The Government Printer of South Africa: Pretoria, 1966).

[60] F. Masson, XVI. An account of three journeys from the Cape Town into the southern parts of Africa; undertaken for the discovery of new plants, towards the improvement of the Royal Botanical Gardens at Kew. By Mr. Francis Masson, one of his Majesty’s gardeners. Addressed to Sir John Pringle, Bart. P.R.S. Phil. Trans. R. Soc. 66, 268–317 (1776).

[61] L. Cheesman, J. J. Nair, J. van Staden, Antibacterial activity of crinane alkaloids from *Boophone disticha* (Amaryllidaceae). J. Ethnopharmacol. 140, 405–408 (2012). 22322252 10.1016/j.jep.2012.01.037

[62] F. Viladomat, J. Bastida, C. Codina, J.J. Nair, W. E. Campbell, Alkaloids of the South African Amaryllidaceae in *Recent Research Developments in Phytochemistry*, S. G. Pandalai Ed., (Research Signpost Publishers, Trivandrum, 1997) pp. 131–117.

[63] S. Tonisi, K. Okaiyeto, L. V. Mabinya, A. I. Okoh, Evaluation of bioactive compounds, free radical scavenging and anticancer activities of bulb extracts of *Boophone disticha* from Eastern Cape Province, South Africa. Saudi J. Biol. Sci. 27, 3559–3569 (2020). 33304167 10.1016/j.sjbs.2020.07.028 PMC7715441

[64] J. J. Nair, J. Bastida, C. Codina, F. Viladomat, J. van Staden, Alkaloids of the South African Amaryllidaceae: A review. Nat. Prod. Commun. 8, 1335–1350 (2013). 24273880

[65] A. S. M. Ibrakaw, Chemical investigation of some species of Amaryllidaceae from the Greater Cape Region of South Africa as a source of bioactive compounds, PhD thesis, University of the Western Cape, (2020).

[66] E. E. Elgorashi, Phytochemistry and pharmacology of the family Amaryllidaceae: An overview of research at RCPGD. Nat. Prod. Commun. 14, 1934578X19872929 (2019).

[67] E. E. Elgorashi, S. E. Drewes, C. Morris, J. van Staden, Variation among three *Crinum* species in alkaloid content. Biochem. Syst. Ecol. 31, 601–615 (2003).

[68] H. Lichtenstein, *Reisen in südlichen Africa in den Jahren 1803*, *1804*, *1805 und 1806* (C. Salfeld: Berlin, 1812). [Travels in southern Africa in the years 1803, 1804, 1805, and 1806].

[69] G. A. Farini, *Through the Kalahari Desert: A Narrative of a Journey with Gun*, *Camera*, *and Note-book to Lake N’Gami and back* (Vol. 32). (S. Lw, Marston, Searle, & Rivington. 1886).

[70] G. W. Stow, *The Native Races of South Africa: A History of the Intrusion of the Hottentots and Bantu into the Hunting Grounds of the Bushmen*. (S. Sonnenschein & Company limited, 1905).

[71] W. E. Stanford, Statement of Silayi, with reference to his life among the Bushmen. Trans. R. Soc. S. Afr. 1, 435–440 (1910).

[72] W. H. I. Bleek, L. Lloyd, *Specimens of Bushman folklore*. (G. Allen limited, 1911).

[73] S. S. Dornan, *Pygmies and Bushmen of the Kalahari: An Account of the Hunting Tribes Inhabiting the Great Arid Plateau of the Kalahari Desert: Their Precarious Manner of Living*, *Their Habits*, *Customs and Beliefs*, *with some Reference to Bushman Art*, *both Early and of Recent Date*, *and to the Neighbouring African Tribes*. (Seeley, Service, 1925).

[74] I. C. Hall, R. W. Whitehead, A pharmaco-bacteriologic study of African poisoned arrows. J Infect Dis 41, 51–69 (1927).

