# Supplementary material for: Direct evidence for poison use on microlithic arrowheads in Southern Africa at 60,000 years ago
Source: Sci Adv. 2026 Jan 7;12(2):eadz3281. doi: 10.1126/sciadv.adz3281 (PMC12778036; doi:10.1126/sciadv.adz3281)
Supplement: Supplementary file 1 — Supplementary Text Figs. S1 to S6 Tables S1 to S3 Data S1 to S19 References [file sciadv.adz3281_sm.pdf]

Science Advances
AAAS

Supplementary Materials for

Direct evidence for poison use on microlithic arrowheads in Southern Africa at 60,000 years ago

Sven Isaksson *et al.*

Corresponding author: Sven Isaksson, sven.isaksson@arklab.su.se

Sci. Adv. **12**, eadz3281 (2026)
DOI: 10.1126/sciadv.adz3281

This PDF file includes:

Supplementary Text
Figs. S1 to S6
Tables S1 to S3
Data S1 to S19
References

Supplementary Text

Materials

The artefacts from Umhlatuzana Rock Shelter were excavated by Jonathan Kaplan in 1985 as part of a rescue excavation during road construction (*26*). They were transported to the KwaZulu-Natal Museum, where they were rinsed, marked with Indian ink and placed in plastic baggies by a curator. The original baggies were new South African bank coin baggies, in which the material was grouped according to context in carboard boxes, and stored in a room that is rigidly climate controlled for good curatorial conditions. The Museum has back-up generators for normal power supply during power outages to maintain ideal storeroom conditions. In 2005 ML was the first person to open these baggies since 1985. Under clean laboratory conditions she selected all the quartz backed microliths from Kaplan's Layer 24 (*26*, p. 5, fig. 6), a context that is most distinctly associated with the Howiesons Poort technocomplex of South Africa (*20*, *26*). From those, 10 quartz artefacts with visible residues (Fig. S1) were selected for sampling out of the 216 backed pieces from this single excavation level (*24*) [RBS XX; PBS XII]). All 10 selected artefacts come from RBS XX only, 001-007 from square J2 and 008-010 from square K2. They were placed in separate baggies until residue extraction in 2021. In total 649 backed pieces were reported for the Howiesons Poort layers at the site (*26*). Our sampling strategy was therefore kept to a bare minimum, safeguarding ample untouched material for future research and long-term preservation.

As an ethno-historical control, we also analysed residues extracted from known poisoned arrows curated at The Museum of Ethnography (Etnografiska museet) in Stockholm, Sweden, under ideally controlled conditions. The arrows were collected by Anders Sparrman (1748–1820) and Carl Peter Thunberg (1743–1828), both mentees of Carl Linnaeus. Sparrman embarked on a journey from Cape Town into the interior in July 1775, returning in April 1776 (*54*). He mentions collecting two quivers with arrows, providing an illustration of one of them that is similar to the one at The Museum of Ethnography (museum accession number 1799.02.075) (Fig. S2). Thunberg undertook journeys from Cape Town into the interior in 1772 and 1775. He too described quivers and poison arrows used by the indigenous groups he encountered and observed the use of *B. disticha* arrow poison in South Africa during the late 1700s (*36*; table S3). Although he does not explicitly mention collecting the arrows, he returned to Sweden with a collection of ethnographic items. The history of Thunberg's collection is unclear, but the quiver in Fig. S2 (museum accession number 1874.01.251), along with its accompanying arrows, is considered to be from his collection.

We also sourced and analysed extracts of modern *B. disticha* bulb exudate from the Bergius Botanical Garden in Stockholm, Sweden to authenticate results obtained from the archaeological and ethnographic material.

Provenance

The archaeological artefacts from Umhlatuzana:

1. The artefacts were excavated from Umhlatuzana Rock Shelter in KwaZulu-Natal South Africa in 1985 by Dr Jonathan Kaplan.
2. They were excavated following standard archaeological procedure, and validated for authenticity by Prof John Parkington (supervisor of the excavator, University of Cape Town), Dr Brian Stuckenberg (Detector of the KwaZulu-Natal Museum), Prof Lyn Wadley (examiner of the excavator, University of the Witwatersrand), and Prof Marlize Lombard (researcher on the Umhlatuzana material since 2005, University of Johannesburg). The deposits were dated in 2010 by Prof Richard Roberts and Dr Zenobia Jacobs (University of Wollongong) using the single-grain OSL method with Marlize Lombard as Principal Investigator.

3. The artefacts are curated by the Human Sciences Department of the KwaZulu-Natal Museum (https://www.nmsa.org.za/departments/human-sciences.html) where they can be accessed for further research through lodging an application with the curators. For destructive sampling and export, a Material Transfer Agreement is require from The KwaZulu-Natal AMAFA and Research Institute (https://amafainstitute.org.za/compliance/) or The South African Heritage Resources Agencies (https://www.sahra.org.za/development-applications-unit/) – depending on the nature of the research and material exported.

The ethnographic control artefacts:
1. The arrows were validated for authenticity and age by Michael Barrett, curator of the African collections at the National Museums of World Culture in Sweden.
2. The arrows are curated at The Museum of Ethnography in Stockholm (https://www.etnografiskamuseet.se/en/) where they can be accessed for further research through lodging an application with the curators.

The modern *Boophone distichia* bulb:
1. The plant materials were authenticated and provided for sampling by Gunvor Larsson, Axel Gustafsson and Lars Gunnar Reinhammar of the Bergius Botanical Garden, Stockholm, Sweden. The sampled specimen has Bergius Botanical Garden accession number: SBT:V:24220, accession year 2016 – sown from seed from an older plant (SBT:V:87 – source H. Wanntorp/T. Höijer 1995).

Methods

Minute samples (< 0.1 mg) were removed from the surface of the archaeological artifacts under microscope using sterile dental probes and scalpels, making sure that only part of the visible residues was sampled for microchemical analysis, avoiding obvious recent contaminations. The samples were immediately transferred to cleaned class vials, sealed in wait for analysis.

Due to the small sample sizes and the risk of laboratory contamination the samples were treated using a cradled dissolution and derivatisation technique: To reduce loss risk during transfer, the dissolution of extractable components from the residues was performed directly in the sample glass vials. To each vial 1000 μl of chloroform and 500 μl of methanol was added, mixed and shaken vigorously using a vortex. The extraction continued by means of ultrasonication for thirty minutes after which the solvent was removed using a gentle flow of nitrogen gas. The samples were immediately treated with 100 μl of bis(trimethylsilyl)trifluoroacetamide with 10% (v) chlorotrimethylsilane for 20 minutes at 70 °C. Access reagent was removed again using a gentle flow of nitrogen gas, and the extractives were immediately dissolved in 50 μl of toluene and transferred to a vial with a glass insert due to the very small volume. Toluene was chosen here, instead of the more commonly used n-hexane, due to the lower volatility of toluene and the very small volumes needed for these samples (*55*).

The analysis was performed on an Agilent 8860 Gas Chromatograph with an HP5 MS UI non-polar capillary column (30m x 250μm x 0.25μm). The injection volume was 1 μl and the injection were done pulsed splitless (pulse pressure 26.1 Psi) at 325 °C using an Agilent 7650A Autoinjector. The oven was temperature programmed with an initial isotherm of two minutes at 90°C. The temperature was then increased by 10 °C per minute to 350 °C followed by a final isotherm of 20 minutes. The carrier gas used was helium (He) at a constant flow rate of 2.0 ml per minute.

The gas chromatograph was connected to an Agilent 5977B Mass Selective Detector via an interface with temperature 350 °C. The fragmentation of separated compounds was done by electronic ionization (EI) at 70 eV. The temperature in the ion source was 230 °C. The mass filter was set to scan in the range m/z 50-800, giving 1.5 scans/sec, and its temperature is 150 °C. Data collection and processing was done using Masshunter 10 and NIST Mass Spectral Search Program 2.3 software and the NIST 2017 MS database.

We adopted first an explorative non-targeted approach, investigating to what extent the extractives from these residues could be identified (cf. *7*). Due to the small sample sizes and the relatively large reduction in volume of extraction media (1500 μl) compared to analytical solvent (50 μl, i.e. 30 times) we were expecting an enrichment in laboratory contaminants even though we used analytical grade solvents and reagents. To tackle this potential problem, it was crucial to run both instrument blanks and method blanks in parallel with the samples to monitor this effect. The instrument blank showed no evidence of serious contamination (Fig. S3.) and the methods blank showed evident effects of the treatment (Fig. S4). Contaminants were identified as a number of different siloxanes, probably deriving from vial cap gaskets etc., and of various kinds of plasticizers and polymer stabilizers deriving from various plastic materials, most likely from packing materials. There were also minute traces of the fatty acid palmitic acid in the methods blank, maybe deriving from handling.

In the non-targeted analysis of the chromatographic and mass spectral data using the Masshunter and NIST Mass Spectral Search Program software's a number of components were detected in a number of the samples that were not present in any of the blanks. Only compounds with a match score better than "poor" were considered (Match score < 600 = Poor match, 600-700 = between Poor and Fair match, 700-800 = Fair match, 800-900 = Good match, > 900 = Excellent match).

Protruding from these analyses were the detection of traces of epibuphanisine (Fig. S5) and buphandrin (Fig. S6), as well as traces of the diterpenoid dehydroabietic acid and fatty acids. To elucidate the presence of these compounds in the samples we produced ion chromatograms of characteristic ion fragments (table S1) of these compounds (Data S1-10).

The ethnographic material was initially analysed slightly differently, but comparably, due to the larger sample sizes available. Approximately 50 mg of arrow poison residue was collected for the analysis. The solvable organic components were extracted using chloroform and methanol (2:1, v:v) by means of ultrasonication for thirty minutes after which the samples were centrifuged for thirty minutes at 3000 rpm. The clear solvent was collected and transferred to vials and the solvent was removed using a gentle flow of nitrogen gas. The samples were immediately treated with 100 μl of bis(trimethylsilyl)trifluoroacetamide with 10% (v) chlorotrimethylsilane for 20 minutes at 70 °C. Access reagent was removed again using a gentle flow of nitrogen gas, and the extractives were redissolved in 200-400 μl of n-hexane.

Also, these analyses were performed on an Agilent 8860 Gas Chromatograph with the same type of column (HP5 MS UI non-polar capillary column, 30m x 250μm x 0.25μm) and using the same injection technique (1 μl injection volume, done pulsed splitless (pulse pressure 26.1 Psi) at 325 °C using an Agilent 7650A Autoinjector). The oven temperature program was adjusted (see below) with an initial isotherm of two minutes at 50°C. The temperature was then increased by 10 °C per minute to 350 °C followed by a final isotherm of 20 minutes. The carrier gas used was helium (He) at a constant flow rate of 2.0 ml per minute. The gas chromatograph was connected to an Agilent 5977B Mass Selective Detector with identical settings as for the authentic samples.

To obtain the so-called solvent effect, which focus the sample at the front of the GC column at the start of an analysis improving peak shapes and performance, the initial oven temperature is adjusted to the boiling point of the sample solvent. Since we used n-hexane as the final solvent the initial isotherm of the GC temperature program was 50 °C in this analysis of reference materials. As stated above toluene was used as solvent for the much more minute samples and the initial isothermal of the GC temperature program was adjusted to the higher boiling-point of this solvent, i.e., to 90 °C. This causes a systematic shift in the retention time of peaks between the two temperature programs but since everything else is kept the same the relative order of peaks is the same between the two setups. Similar to some of the archaeological quartz backed pieces from Umhlatuzana Rock Shelter, we found epibuphanisine (Fig. S5) and buphandrin (Fig. S6) in the poison residues of four of these arrows (Fig. S2), for chromatograms see Data S11-S14.

To verify the authenticity of the detection of these two alkaloids in the minute archaeological samples, we sub-sampled and re-analyzed the four ethnographic samples using exactly the same procedure and equipment as for the samples extracted from the quartz microliths. The chromatographic and mass spectrometric results are matching, see Data S15-18. To further authenticate the identification of epibuphanisine and buphandrin recorded on both the archaeological and ethnographic arrow tips, we sampled extracts from a *B. distichia* bulb provided by the Bergius Botanical Garden, Stockholm, Sweden (Bergius Botanical Garden accession number: SBT:V:24220, accession year 2016 – sown from seed from an older plant (SBT:V:87 – source H. Wanntorp/T. Höijer 1995)). Using the same procedure and equipment as for the archaeological samples, and for the second analysis of the ethnographic samples, we obtained identical chromatographic and mass spectrometric results for the modern *B. disticha* bulb exudate, see Data S19.

The detection of unknown compounds would require the use of high-resolution mass spectrometry (which we do not have access to at present, but intend to incorporate in future work to explore more complex or less well-characterized constituents such as potential decomposition products). Identifying the same known compounds (i.e., buphanidrine and epibuphanisine) in extracts from the authentic plant tissue (*B. disticha* bulb), however, in authentic ethno-historical arrow poison samples from the same geographical region as the archaeological samples, with a well-known history of *B. disticha* arrow poison – using the same chromatography and EI mass spectrometry – provides the most robust contextual and chemical support for the identification of ancient arrow poisons presented to date.

The chemical and physical properties of buphanidrine and epibuphanisine

Buphanidrine has a topological polar surface area (TPSA) of 40.2 Å² and a computationally predicted octanol-water partition coefficient (XlogP) of 2.2, indicating moderate hydrophobicity. It possesses five hydrogen bond acceptors, no hydrogen bond donors, and contains only two rotatable bonds. The five hydrogen bond acceptors of buphanidrine suggest it can interact well with hydrophilic mineral surfaces, especially those with hydroxyl groups such as silica. However, pH, water competition, and mineral type will significantly affect how strongly it adsorbs. In dry environments, adsorption is likely stronger, while in high-water-content systems, competition with water may reduce binding efficiency. However, the lack of hydrogen bond donors provides buphanidrine with a fairly low solubility in water. Since buphanidrine lacks hydrogen bond donors, it is also less prone to both oxidation and hydrolysis, which increases its stability in various conditions. While hydrogen bond acceptors allow some interaction with water and biological molecules, they don’t make the molecule highly reactive. The low number of rotatable bonds makes the compound structurally rigid, and less likely to undergo conformational changes in

different environments. This rigidity can enhance stability in biological systems and prevent degradation via conformational shifts. With an XlogP of 2.2, buphanidrine is moderately lipophilic, meaning it can cross membranes but is not overly hydrophobic, reducing the risk of rapid fat-based degradation. This balance suggests that buphanidrine is not overly hydrophilic or reactive, meaning it won't break down too easily in aqueous environments. In living cells, it can be predicted to be metabolized by enzymes, suggesting some metabolic processing but not necessarily rapid degradation. The very low TPSA of 40.2 indicate a high probability of blood-brain barrier penetration also indicating that it would remain chemically intact long enough to reach the central nervous system. This however, has more to do with the toxic properties of the compound. In conclusion, buphanidrine is a comparatively stable compound, due to its rigidity, lack of hydrogen bond donors, and moderate lipophilicity.

While specific values for epibuphanisine are not readily available in standard databases (such as https://www.ncbi.nlm.nih.gov/pccompound), we can estimate some of the properties of epibuphanisine based on its chemical structure. Estimates for TPSA is 75-90 Å² and for XlogP it is 2.5-3.5. Further, epibuphanisine contain four hydrogen bond acceptors, no hydrogen bond donors, and one rotatable bond. This suggests that epibuphanisine is slightly more lipophilic than buphanidrine and slightly less prone to adsorption to mineral surfaces, but potentially less prone to conformational changes.

Molecular Age of Amaryllidaceae

According to Costa et al. (*40*), the crown node of Amaryllidaceae appeared 67.9 Mya (77.2-58.5 Mya: 95% HPD), with a probable Gondwanan stem age and distribution in parts of South America, Africa, and India. From there, the three main subfamilies followed different evolutionary paths. The most recent common ancestors of Agapanthoideae and Amaryllidoideae diversified from Africa approximately 62.7 Mya (68.4-55.8 Mya: 95% HPD), with the former remaining in Africa, while the latter colonized regions of Europe, Asia and South America. Allioideae split shortly after separating from the other subfamilies 63.2 Mya (67.5-53.7 Mya: 95% HPD). One of the lineages became Allieae 52.2 Mya (58.1-44.4 Mya: 95% HPD), rapidly colonizing parts of Asia and North America after arriving presumably via the Indian Subcontinent. From there, Allieae colonized most regions of the Northern hemisphere. The other lineage split 54.1 Mya (65.1-37.11 Mya: 95% HPD) into Tulbaghieae and Gilliesieae. Tulbaghieae did not expand from Africa while Gilliesieae diversified in South America, splitting further into the more widespread Gilliesiinae and the Chilean Andean Leucocorynae approximately 45 Mya (61.232.2 Mya: 95% HPD). The Amaryllidoideae subfamily to which all the indigenous species that could have been the sources for buphandrin, epibuphanisine belong was present in southern Africa by 68.4-55.8 Mya (*40*). Based on the Costa et al. (*40*) phylogenetic analysis, an age estimate for current southern African Amaryllidaceae that contain these alkaline toxins, such as *Ammocharis* spp., *Boophone disticha*, *Brunsvigia* spp. *Crinum* spp. *Crossyne* spp. and *Nerine* spp. (table S2), seem to have grown in the region by 20-10 Mya (*40*). They probably evolved as part of the primary grassland of southern Africa that originated during the Oligocene climate change, between ~ 33.9 and 23 Mya, becoming increasingly widespread thereafter (e.g., *56*, *57*), coinciding with molecular age estimates for early Miocene (~ 21–18 Ma) grass families (*58*).

Boophone distichia and its uses

B. disticha (Linné fil.) Herbert occurs from southeast Sudan to South Africa. It is widespread in grassland, savanna and Karoo vegetation. Synonyms include *Amaryllis disticha* Linné fil., *Brunsvigia disticha* (Linné fil.) Sweet, *Haemanthus distichus* (Linné fil.) Linnéfil. ex Savage, *Haemanthus ciliaris* Linné, *Brunsvigia ciliaris* (Linné) Ker-Gawler, *Haemanthus toxicarius* Linné

fil. ex Aiton, *Brunsvigia toxicaria* (Linné fil. ex Aiton) Ker-Gawler, *Boophone toxicaria* (Linné fil. ex Aiton) Herbert, *Amaryllis toxicaria* (Linné fil. ex Aiton) D. Dietrich, *Haemanthus sinuatus* Schultes & Schultes fil., *Boophone intermedia* M. Roemer, *Boophone longipedicellata* Pax, *Haemanthus robustus* Pax, incl. *Brunsvigia rautanenii* Baker, and *Haemanthus lemairei* De Wildeman (*41*). The rootstock is a bulb up to 30 cm in diameter. Bulbs are solitary, growing partly above ground, recognised by the thick layers of papery scales surrounding the fleshy inner part, from which the symmetrically arranged leaves emerge after flowering. The flowers are a deep rose colour (pink to reddish), borne in a rounded inflorescence. The fruiting umbel breaks off and is blown along by the wind, the capsules breaking and liberating the seeds (*41, 59*).

Neuwinger (*4*) describes *B. disticha* as the main hunting poison in the south of Africa, where it was in especially plentiful supply. Most observations of such use were made before 1900 (table S3). The main users were the Nama Khoe from Little Namaland in Namibia as well as San hunter-gatherers scattered throughout the southeast Kalahari around the Gariep (previously the Orange River) in the Northern Cape of South Africa, as well as those living in the Western and Easten Cape. Masson (*60*), travelling with Thunberg, was the first to report that the Khoe used the bulb as one of their arrow poison sources. Paterson (*45*) noted that the leaves were fatal to cattle, and the bulb and leaves proved toxic to sheep. The outer bulb scales are used by indigenous peoples as antiseptic wrapping after circumcision (*59*). Based on the antibacterial activity of crinane alkaloids, *B. disticha* has remarkable preservation properties (*61*), and may preserve better than other organics. It has been found in archaeological contexts other than poisoned arrows. For example, a 500-year-old cattle-horn medicine container from the Eastern Cape, South Africa, was found wrapped in a bundle of *B. disticha* leaves and grass to help protect and preserve it (*35*). At Boomplaas Cave in the Western Cape, storage pits dating to 1400 and 4100 BP, and used to store *Pappea capensis* seeds, amongst other things, were lined with *B. disticha* bulbar leaves (*33*). In the Kouga Mountains, Eastern Cape, hunter-gatherers wrapped a man 2000 years ago in the bulb scales of *B. disticha*, which helped to mummify his body (*34*). It is also a highly valued medicinal plant with hallucinatory effects (*4*).

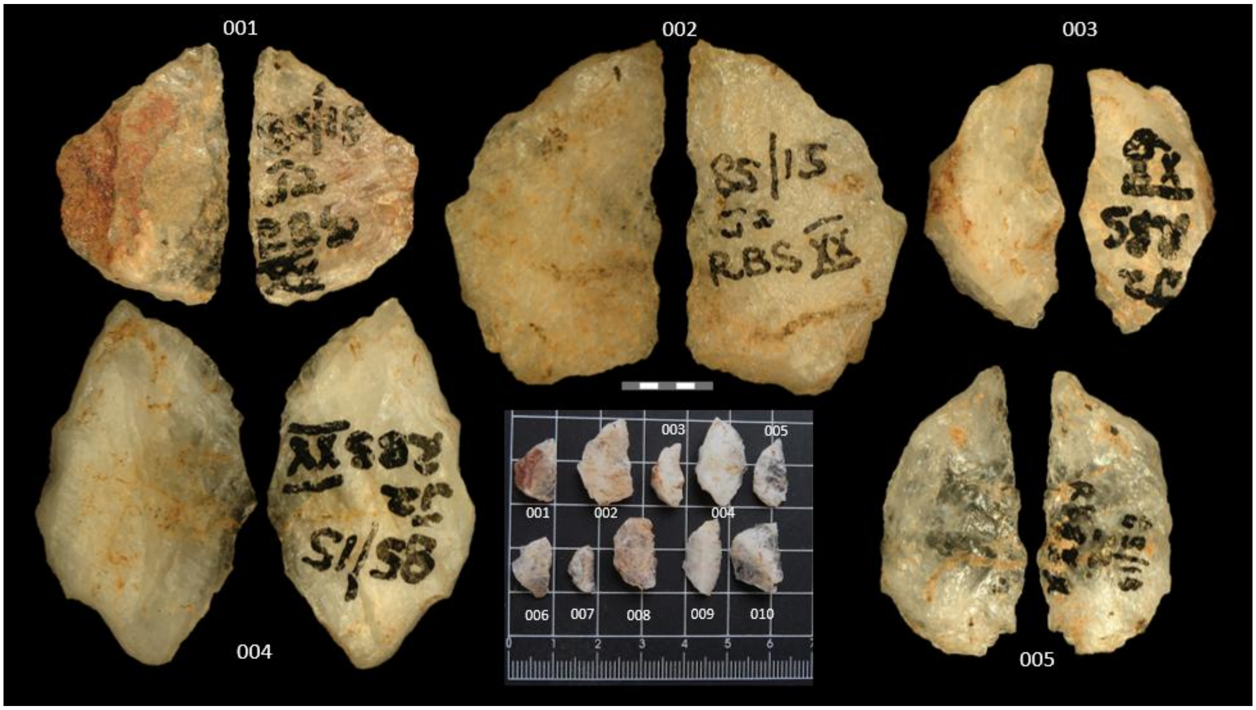

Fig. S1. Closeup of the five quartz backed microliths from Umhlatuzana Rock Shelter with buphandrin, epibuphanisine alkaloid toxins (001-005 [artefact numbers = sample numbers] scale = 5 mm). The inset shows all ten archaeological artefacts analysed, all curated at the KwaZulu-Natal Museum in Pietermaritzburg.

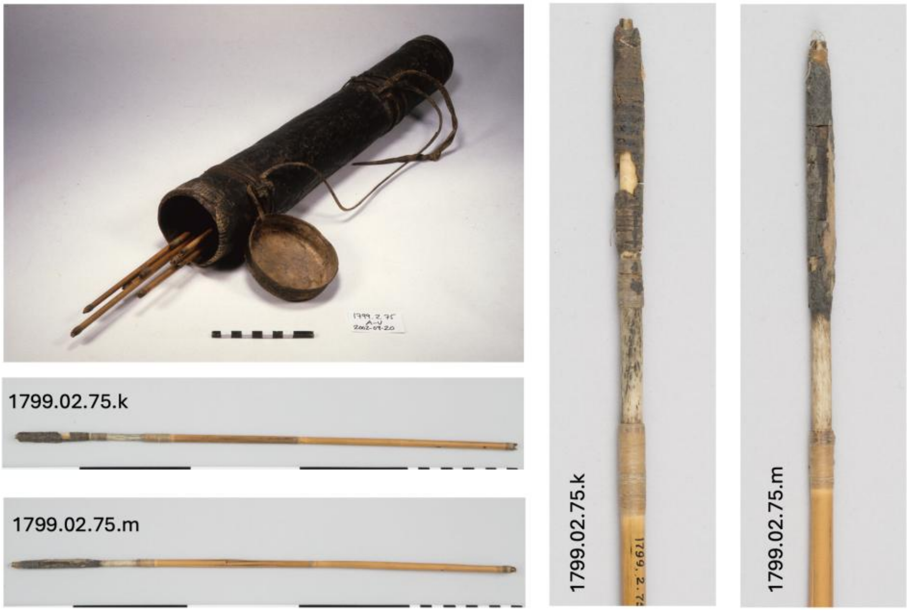

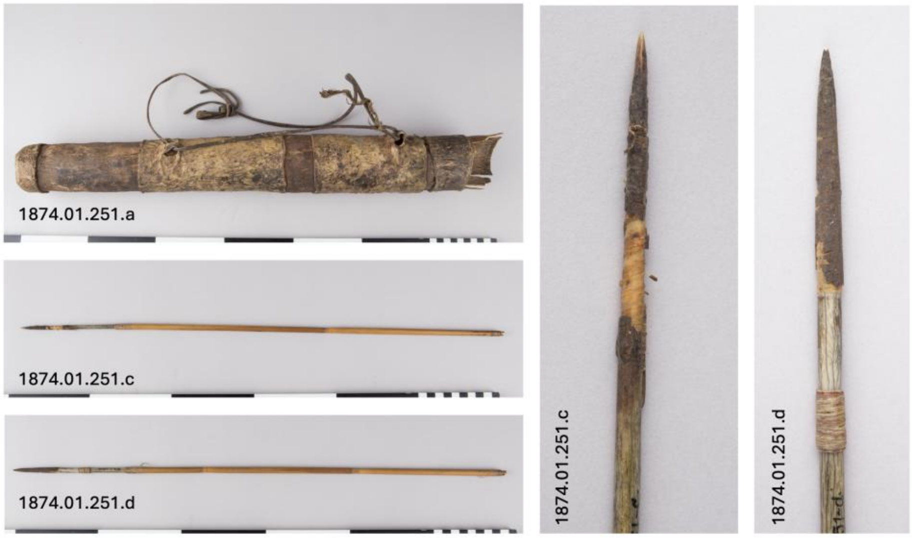

Fig S2.
Ethno-historically collected arrows from South Africa with buphandrin, epibuphanisine alkaloid toxins. Top: Quiver (museum accession number 1799.02.075) and two poison-arrows, both with close up on tip (museum accession number 1799.02.075.k and 1799.02.075.m), collected by Sparrman 1775–1776. Photos of quiver with arrows, Lars-Erik Barkman. Photos of single arrows by Johan Jeppson (Deed - Attribution 4.0 International - Creative Commons).
Bottom: Quiver (museum accession number 1874.01.251) and two poison-arrows, both with close up on tip (1874.01.251.c and 1874.01.251.d), collected by Thunberg in 1772 or 1774. Photos by Johan Jeppson (Deed - Attribution 4.0 International - Creative Commons).

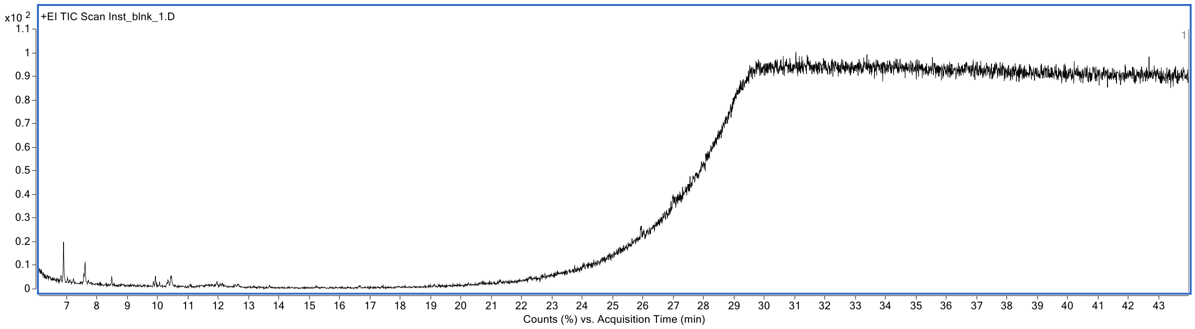

Fig. S3.
Total Ion Chromatogram of the instrument blank.

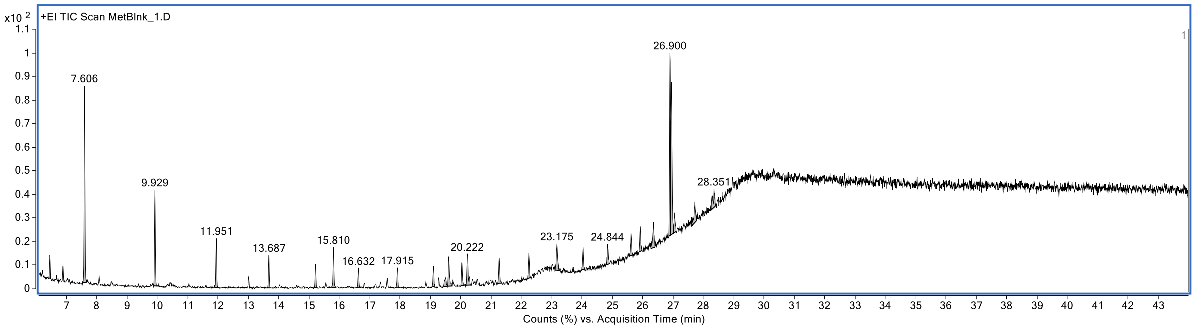

Fig. S4.

Total Ion Chromatogram of the method blank. Through the non-targeted procedure, the peaks are identified as different siloxanes, plasticizers and polymer stabilizers.

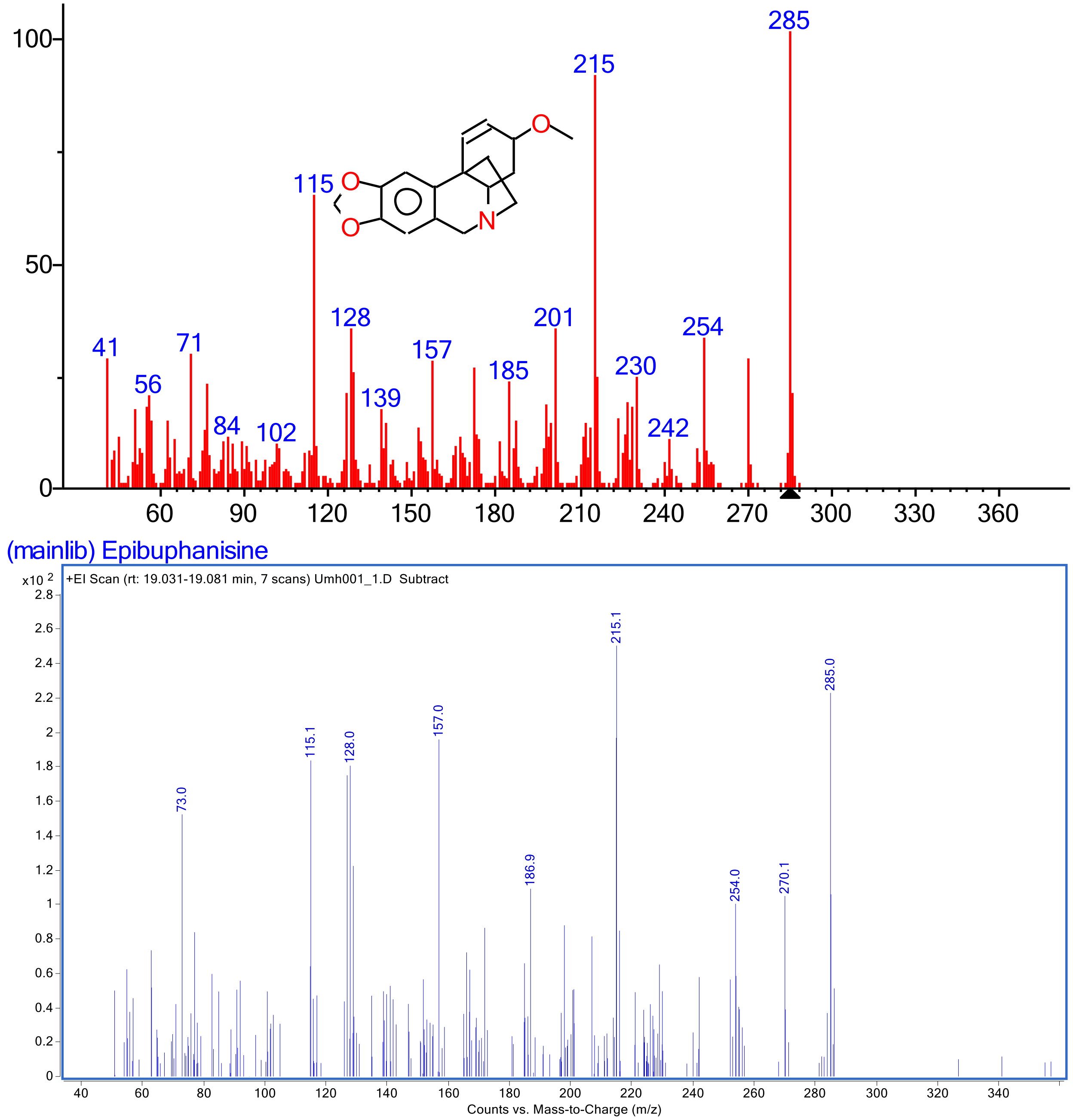

Fig. S5.
Mass spectra of epibuphanisine. a) From the NIST 2017 MS database. b) From sample 001, match score for the sample compared to this standard is 729 = Fair match.

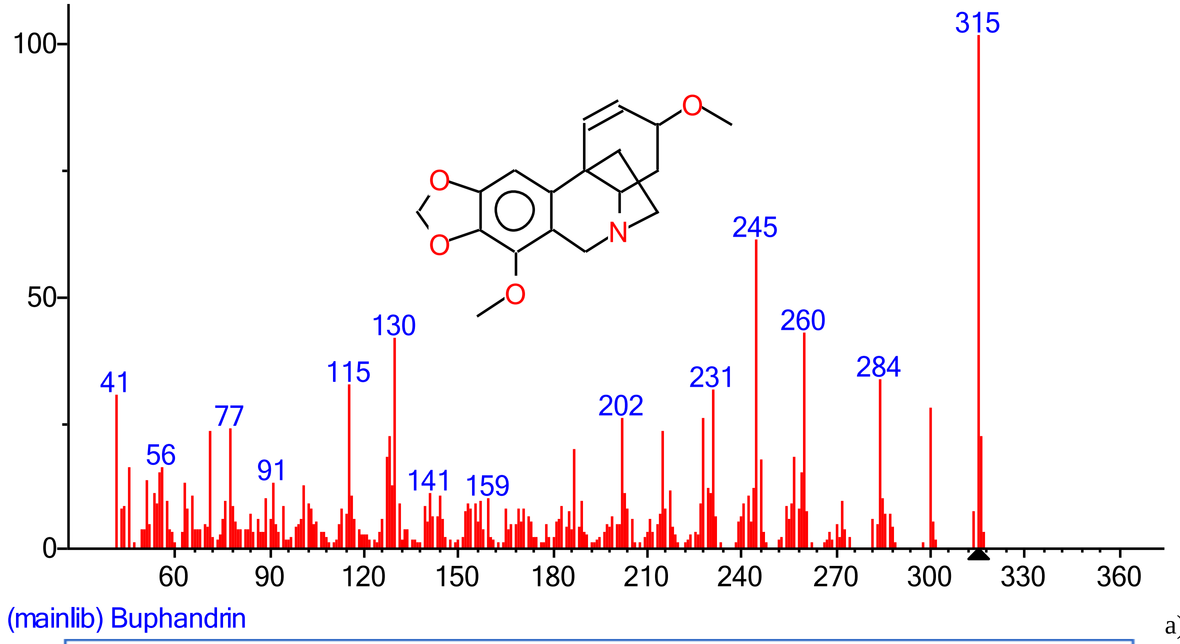

a)

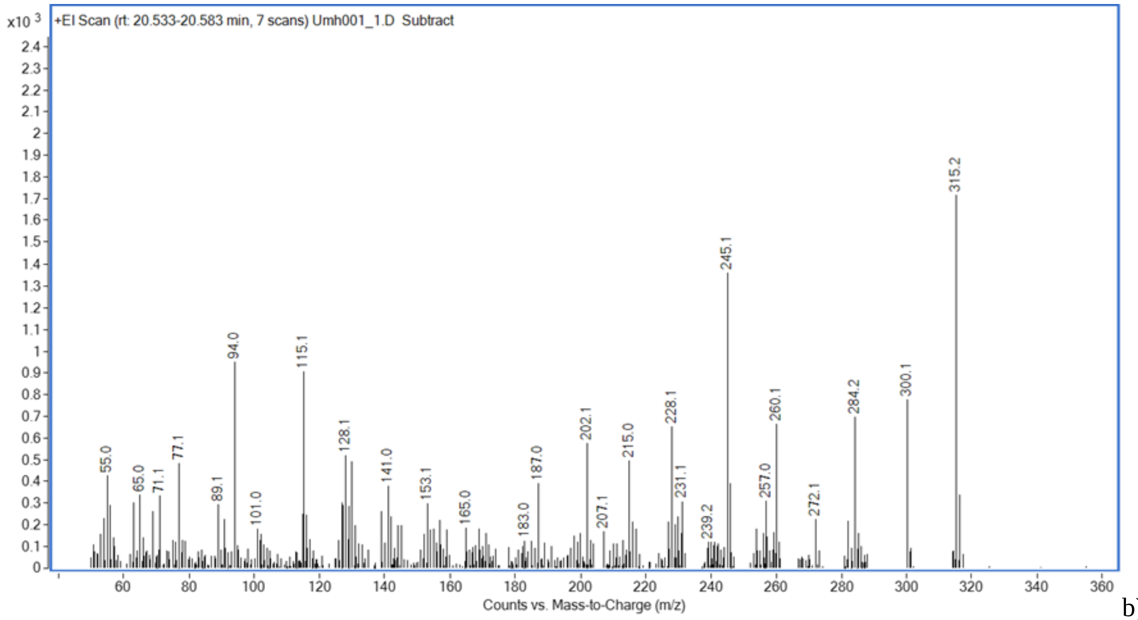

b)

Fig. S6.

Mass spectra of Buphandrin. a) From the NIST 2017 MS database. b) From sample 001, match score for the sample compared to this standard is 873 = Good match.

Table S1.

Characteristic ion fragments of compounds used to produce ion chromatograms (Data S1-S14).

Ion Fragment	Compound
115	Epibuphanisine, buphandrin
117	Fatty acids
130	Buphandrin
157	Epibuphanisine
215	Epibuphanisine
239	Dehydroabietic acid
245	Buphandrin
254	Epibuphanisine
260	Buphandrin
285	Epibuphanisine
315	Buphandrin

Table S2.

South African indigenous plant sources for buphandrin and epibuphanisine. Their current distribution according to the open-source Global Biodiversity Information Facility (https://www.gbif.org/what-is-gbif, visited March 2025). Red marker indicated the location of Umhlatuzana Rock Shelter.

**Potential source**	**Current distribution**
Amaryllis belladonna L.<br>https://www.gbif.org/species/2687632<br>Buphandrin: (*37*)<br>Indigenous to: South Africa<br>Grows: >100 km from Umhlatuzana<br>Family: Amaryllidaceae<br>Sub-family: Amaryllidoideae<br>Common names: belladonna lily, March lily, naked lady (Eng.); Maartblom, Maartlelie, belladonnalelie (Afr.)<br>https://pza.sanbi.org/amaryllis-belladonna	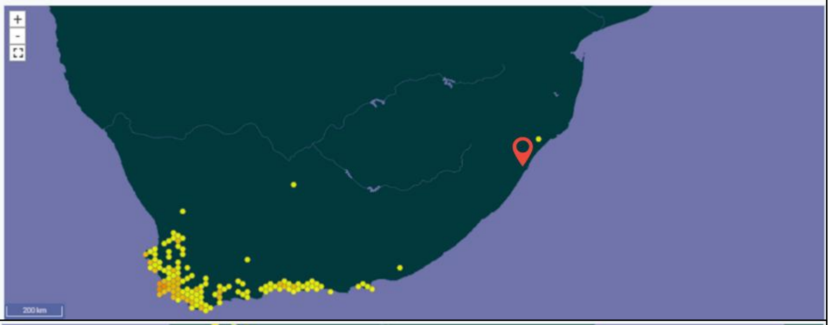
Ammocharis coranica (Ker Gawl.) Herb.<br>https://www.gbif.org/species/5325614<br>Epibuphanisine: (*62*)<br>Indigenous to: South Africa<br>Grows: <70 km from Umhlatuzana<br>Family: Amaryllidaceae<br>Subfamily: Amaryllidoideae<br>Common names: karoo lily, koranna lily, sore eye flower, bible flower, ground lily (Eng.); berglelie, gifbol, seeroogblom (Afr.); boka (Sesotho); isidiya, icukudo, incotho (isiZulu)<br>https://pza.sanbi.org/ammocharis-coranica	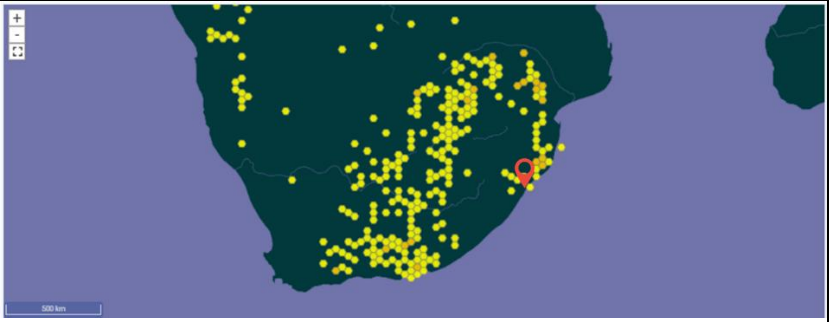
Boophone disticha (L.f.) Herb.<br>https://www.gbif.org/species/2854664<br>Buphandrin & Epibuphanisine: PubChem database, (*62*, *63*) Indigenous to: South Africa & KwaZulu-Natal<br>Grows: <12.5 km from Umhlatuzana<br>Family: Amaryllidaceae<br>Subfamily: Amaryllidoideae<br>Common names: century plant, poison bulb, sore-eye flower (Eng.); gifbol, seeroogblom, kopseerblom, boesmangif, perdespook (Afr.); kxutsana-yanaha, motlatsisa (South Sotho); incumbe, siphahluka (Swazi); incotho, incwadi<br>(Xhosa, Zulu); ibhade (Zulu)<br>https://pza.sanbi.org/boophone-disticha	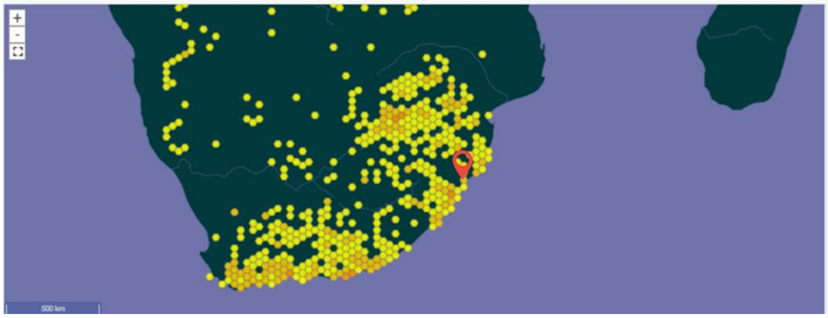
Boophone haemanthoides F.M. Leight.<br>https://www.gbif.org/species/2854663<br>Buphandrin: (*64*, *65*)<br>Indigenous to: South Africa<br>Grows: >1000 km from Umhlatuzana<br>Family: Amaryllidaceae<br>Subfamily: Amaryllidoideae<br>Common names: Namaqua century plant (Eng.); gifbol, kwaslelie (Afr.)<br>https://pza.sanbi.org/boophone-haemanthoides	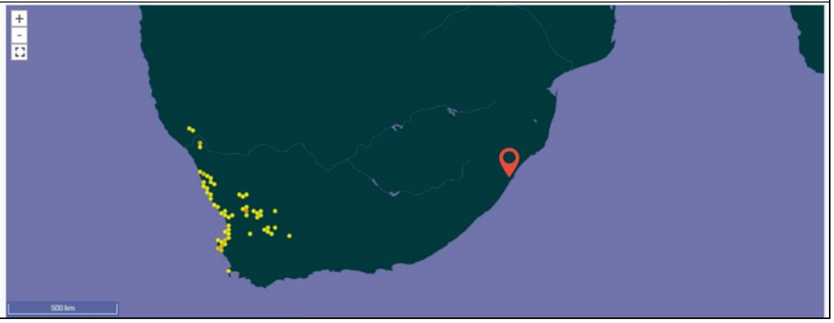

Brunsvigia josephinae (Redoute) Ker Gawl.<br>https://www.gbif.org/species/4013911<br>Buphandrin: (*62*)<br>Indigenous to: South Africa<br>Grows: >500 km from Umhlatuzana<br>Family: Amaryllidaceae<br>Subfamily: Amaryllidoideae<br>Common names: candelabra lily (Eng.); kandelaarblom, lantanter (Afr.)<br>https://pza.sanbi.org/brunsvigia-josephinae	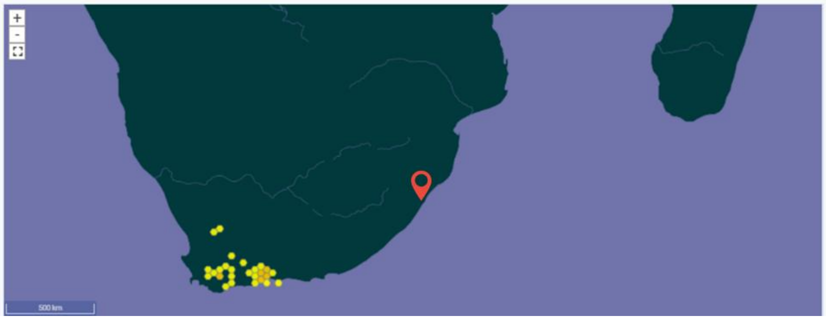
Brunsvigia orientalis (L.) Aiton ex Eckl.<br>https://www.gbif.org/species/2853381<br>Buphandrin & Epibuphanisine: (*62*)<br>Indigenous to: South Africa<br>Grows: >500 km from Umhlatuzana<br>Family: Amaryllidaceae<br>Subfamily: Amaryllidoideae<br>Common names: candelabra flower, red candelabra flower, king candelabra, chandelier lily, chandelier plant, sore-eye flower (Eng.), ajuinbol, koningskandelaarblom, kandelaarblom, kandelaarlelie, lantanter, Maartblom, marsblom, perdespookbossie, rolbossie, seeroogblom and tanteletant (Afr.)<br>https://pza.sanbi.org/brunsvigia-orientalis	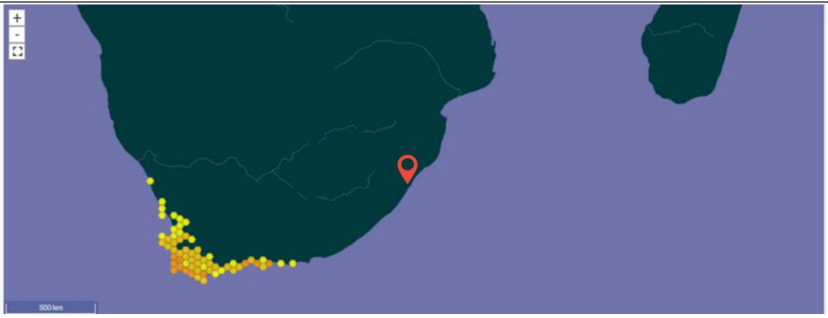
Crinum bulbispermum (Burm.f.) Milne-Redh. & Schweick.<br>https://www.gbif.org/species/2853668<br>Epibuphanisine: (*66*)<br>Indigenous to: South Africa, KwaZulu-Natal<br>Grows: <70 km from Umhlatuzana<br>Family: Amaryllidaceae<br>Subfamily: Amaryllidoideae<br>Common names: Orange River lily, Vaal River lily (Eng.); Oranjerivierlelie (Afr.); umnduze (Zulu).<br>https://pza.sanbi.org/crinum-bulbispermum	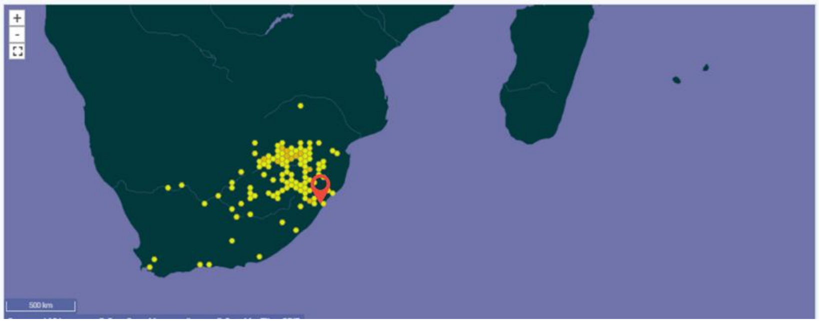
Crinum macowanii Baker<br>https://www.gbif.org/species/2853929<br>Buphandrin & Epibuphanisine: (*37, 67*)<br>Indigenous to: South Africa & KwaZulu-Natal<br>Grows: ≤12.5 km from Umhlatuzana<br>Family: Amaryllidaceae<br>Subfamily: Amaryllidoideae<br>Common names: river crinum, river lily, common vlei-lily, Sabie crinum, Cape coast lily (Eng.); rivierlelie, boslelie, Sabielelie (Afr.); intelezi (isiXhosa); umduze (isiZulu)<br>https://pza.sanbi.org/crinum-macowanii	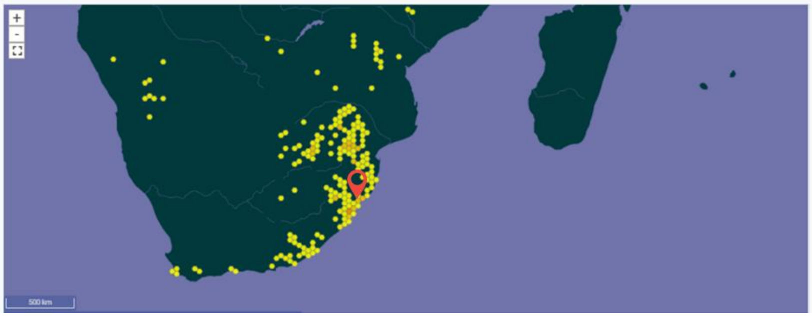
Crinum moorei Hook.f.<br>https://www.gbif.org/species/4928395<br>Epibuphanisine: (*66, 67*)<br>Indigenous to: South Africa & KwaZulu-Natal<br>Grows: ≤12.5 km from Umhlatuzana<br>Family: Amaryllidaceae<br>Subfamily: Amaryllidoideae<br>Common names: Natal lily, Moore's crinum, Ngomi lily, Inanda lily (Eng.); boslelie, Natallelie, Ngomilelie, rivierlelie (Afr.); umnduze (Zulu)	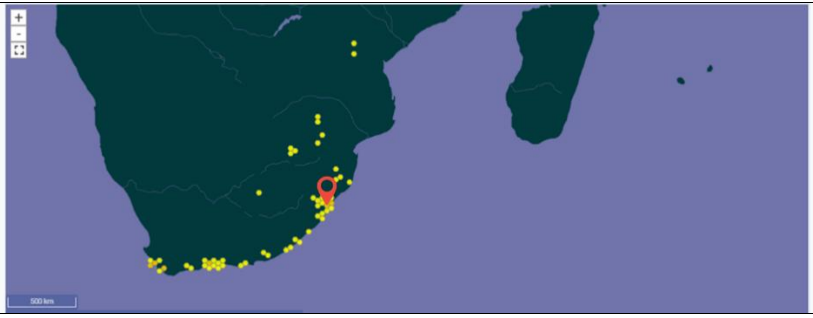

https://pza.sanbi.org/crinum-moorei	
Crossyne flava (W.F.Barker ex Snijman) D.Mull.-Doblies & U.Mull.-Doblies [a.k.a. *Boophone flava* W.F.Barker ex Snijman]<br>https://www.gbif.org/species/2853222<br>Buphandrin & Epibuphanisine: (*62*, *65*)<br>Indigenous to: South Africa<br>Grows: >1000 km from Umhlatuxana<br>Family: Amaryllidaceae<br>Subfamily: Amaryllidoideae<br>Common names: Parasol Lily (Eng.) geelsambreelblom (Afr.)<br>http://redlist.sanbi.org/species.php?species=5349-2	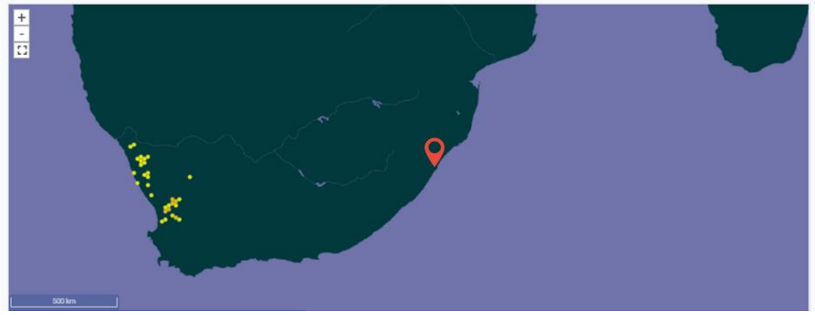
Nerine bowdenii W.Watson<br>https://www.gbif.org/species/5326845<br>Buphandrin: (*62*, *65*)<br>Indigenous to: South Africa & KwaZulu-Natal<br>Grows: >150 km from Umhlatuzana<br>Family: Amaryllidaceae<br>Subfamily: Amaryllidoideae<br>Common names: large pink nerine (Eng.); grootpienknerina (Afr.)<br>https://pza.sanbi.org/nerine-bowdenii	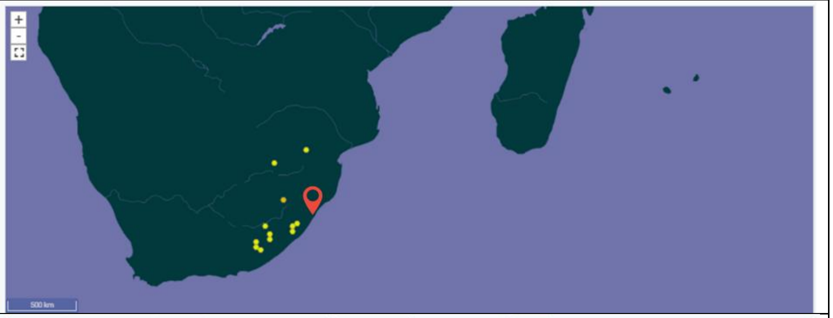
Nerine undulata (L.) Herb. [a.k.a. *Nerine Crispa* Hort.]<br>https://www.gbif.org/species/5326908<br>Buphandrin: (*62*)<br>Indigenous to: South Africa<br>Grows: >100 km from Umhlatuzana<br>Family: Amaryllidaceae<br>Subfamily: Amaryllidoideae<br>Common names: nerine (Eng.); seeroogblom, berglelie (Afr.)<br>https://pza.sanbi.org/nerine-undulata	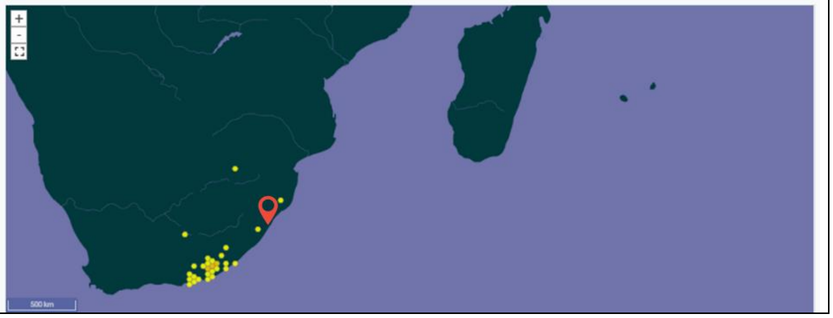

Table S3.

Ethno-historical records of *Boophone distichia* used as arrow poison in southern Africa.

**Arrow poisons** (non-verbatim, but accurate excerpts from the original texts)	**Region**	**Source**
About fifty miles N.N.W. from Cape Town, we found a great variety of curious plants, in particular a large bulbous root, growing on dry precipices, which the Dutch call *vergift-boll*, poison bulb; the juice of which, they say, the Khoe use as an ingredient to poison their arrows. We found it to be a species of Amaryllis, and by the leaves growing in a fan shape, we called it *Amaryllis disticha*.	South Africa, Western Cape (Khoe)	*60*
Gifbol poison is also called 'Mad Poison', from the effects it usually produced on the animals that are wounded by the weapons impregnated with it. The indigenous people prepare this poison in the following manner: They take the bulbs around the time when the plants are pushing out their leaves, and cut them transversely to extract a thick fluid. The fluid is kept in the sun until it becomes the consistency of gum, when it can be used to poison arrows. The hunters employ poison of this plant species mainly for the purpose of killing animals intended for food, such as antelopes and other small quadrupeds. After the animals are wounded, they can, and do in general, run for several miles; and it frequently happens that they are not found until the next day, notwithstanding the poisonous substance having penetrated their muscles.	South Africa, Cape (Khoe and San)	*45*
The root, which is poisonous, is almost as big as one's fist. The Khoe use it chiefly for poisoning arrows with which they shoot smaller game, such as springbok and the like. Bulbs that grow in the shade are thought to possess stronger poison than those exposed to the sun.	South Africa, Western Cape (Khoe)	*36*
Extracted from the bulb is a sharp alkali, and when delivered into the bloodstream, decomposes it immediately. The San call this sap *bolletjies* poison. If used against a man, they add a larger proportion of animal poisons, if used against animals in chance hunting, then the *Boophone* sap is the prevailing ingredient. Although these people know very well that the poisons are only noxious when delivered into the bloodstream, they cautiously avoid preparing it with their naked hands. Instead, the poison is mixed with a wooden stick, in a hollow stone that has been heated. They are particularly cautious in smearing their arrows, since they might easily wound themselves by giving an awkward turn to the weapon while they are doing it. For this purpose, a stone with a small channel or gutter is filled with the poison, the point of the arrow is laid into it, and the mixture rubbed on with the little stick until the proper quantity is imbibed.	South Africa, Cape (San)	*68*
In front of them there lay 30-40 bulbs, which I recognised as poison bulbs by their fan-like leaves, which bear such beautiful flowers. The ends of the roots were cut off and laid on the silky-looking dried scales which they had removed from the external hull of the bulb, and a milky liquid trickled out. When the juice stopped flowing, new cuts were made about an inch deep, and the juice began to flow out again; so it went on until the bulb was empty. Approximately 2 quarts (about 2 litres) of the milky exudation was thus collected in one of our round iron pots, which we had up to now used to hold our wagon grease, and it was set on the fire. The poison glands of a number of newly-caught snakes, powdered dried spiders and ground stone to thicken were added to the mass using a small piece of metal, the 15 cm-long wooden arrow points were smeared with the thick poison mass and dried in the sun. The remaining poison was formed into small balls, which were carried as extra supplies and warmed when needed.	Kalahari (San)	*69*
Amaryllis (*gifbol*) sap was mixed with snake venom, crushed scorpions and spiders, and other milky plant extracts. The people adopted the following method in the preparation of their poisons. A flat, smooth, stone was procured, very similar to the one used for roasting ant eggs; this was placed on the fire, and the milky juice of the *Motlatsisa* or of Amaryllis was set upon it. This was then worked up with a wooden spatula until it began to attain a certain consistency, when snake venom or other poisonous ingredients were gradually added, and the mixing continued until the whole mass had acquired a dark wax-like appearance, when it was worked up into a lump and reserved for use. The season for making this preparation was during the summer months. The poison was kept in a little bag until required for use. In employing it, a small portion was placed upon a poison-stone, and the part of the arrow to be anointed pressed upon it and worked round-and-round until it had acquired the proper shape. The poison was never touched with the fingers, and great care was taken that none adhered to the hands or nails during these processes.	South Africa, Northern Cape, Gariep (San)	*70*
The poison was prepared by Ngqabayi, the chief. He used the root of a shrub mixed with the bark of a tree. I know the shrub, but not the kind of tree from which he got the bark. The root and bark were boiled together in a clay pot until they became a black-looking jelly. It took days to prepare the poison; when ready, Ngqabayi served it out. The poison was deadly. Hartebeest died from it quickly, also gnus. Buffaloes were stronger and lived longer. If we wounded a buffalo in the daytime, we expected to find it dead the next morning.	South Africa, Easten Cape (San)	*71*
It is like a pumpkin, it is round. Its juice is white; it is like water. Its juice is not a little white; its whiteness resembles milk. It is poison. We make an incision and set it down; and then we hold a tortoiseshell underneath it because we wish its juice to be upon the tortoiseshell, that we may make *lkwqi* of it. And we warm it by the fire, making it hot; and we beat it, when it is hot. Then, we beat cooling it. And we take it up in this manner with a *Driedoorn* (*Rhigozum trichotomum*) stick; as we make it round while we think that we intend to make little springbok arrows.	Northern Cape (/Kam San)	*72*
The *gifbol* juice is first extracted and put into a pot to boil, being constantly stirred. To this is added the scraped root of *Aconcanthera*, and still further boiled. When it is sufficiently boiled, the poison bags of snakes (puffadder or cobra) are added, either whole or dried and pounded, and finally, though not in all cases, pounded caterpillar is added. The mixture, after boiling for sometime longer, is tested for its consistency, and if found satisfactory is taken off the fire and emptied into moulds made of hollow pebbles. The moulds into which the poison is poured have little spiral grooves or canals leading from each side to the hollow. The use of these is to prevent accidents to the fingers of the hand when the poison is smeared on the arrows. The arrow point is laid in the groove and gently pushed along, being turned in the process, so that the poison is laid on uniformly. It is then set in the wind and soon dries, into what looks very much like wax.	Kalahari (San)	*73*
Boophone disticha exudate is used with exudates from *Strychnos* and *Euphorbia* species as additive to snake venom and *Diamphidia* poison.	Namibia (Hei//om San, Ju/wasi San)	*74*

Data S1.

Ion chromatogram Sample 001 of characteristic ions for epibuphanisine, buphandrin, fatty acids and dehydroabietic acid (cf. Tab. S1). Compound acquisition time & match score: Palmitic acid 15.818 min., 876; stearic acid 17.588 min., 819, dehydroabietic acid (?) 18.872 min., 598; epibuphanisine 19.056 min., 729; buphandrin 20.549 min., 873.

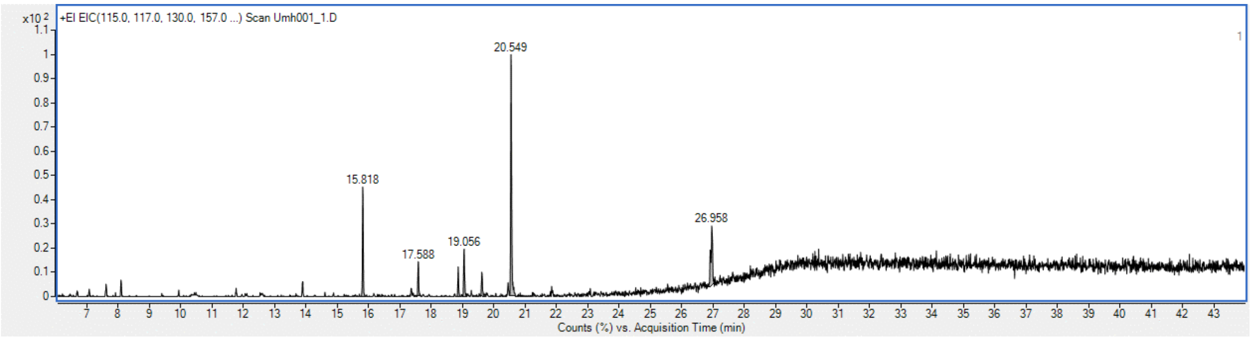

Mass spectrum identified as Epibuphanisine (match score 729) in Sample 001.

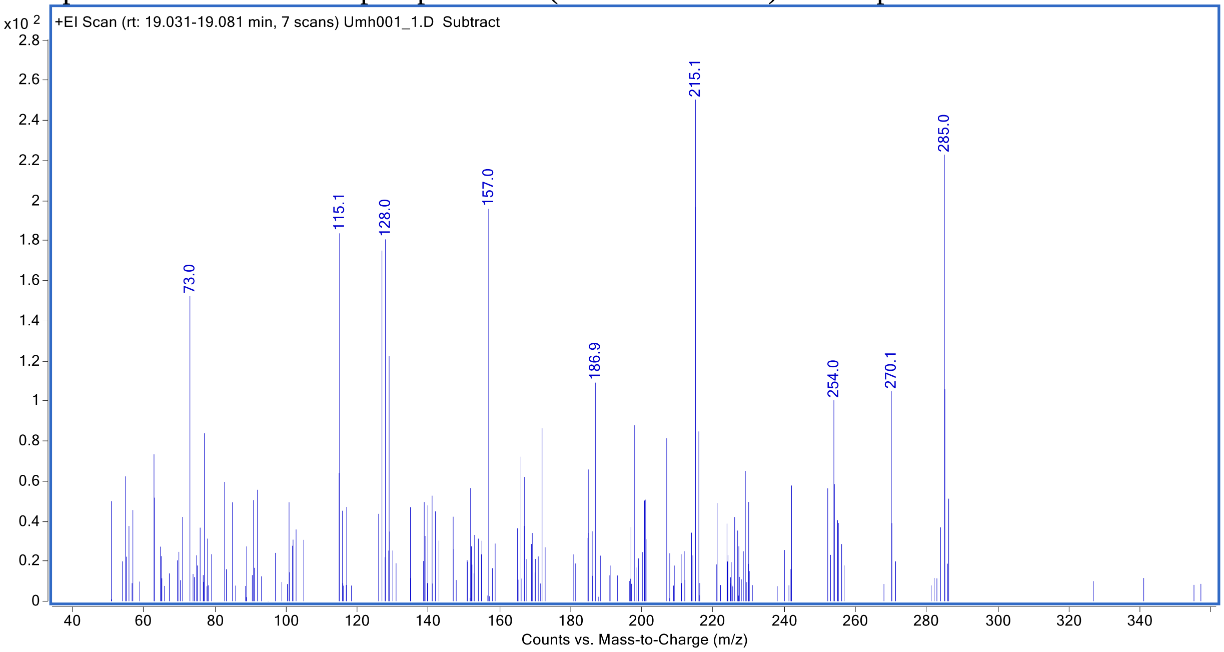

Mass spectrum identified as buphandrin (match score 873) in Sample 001.

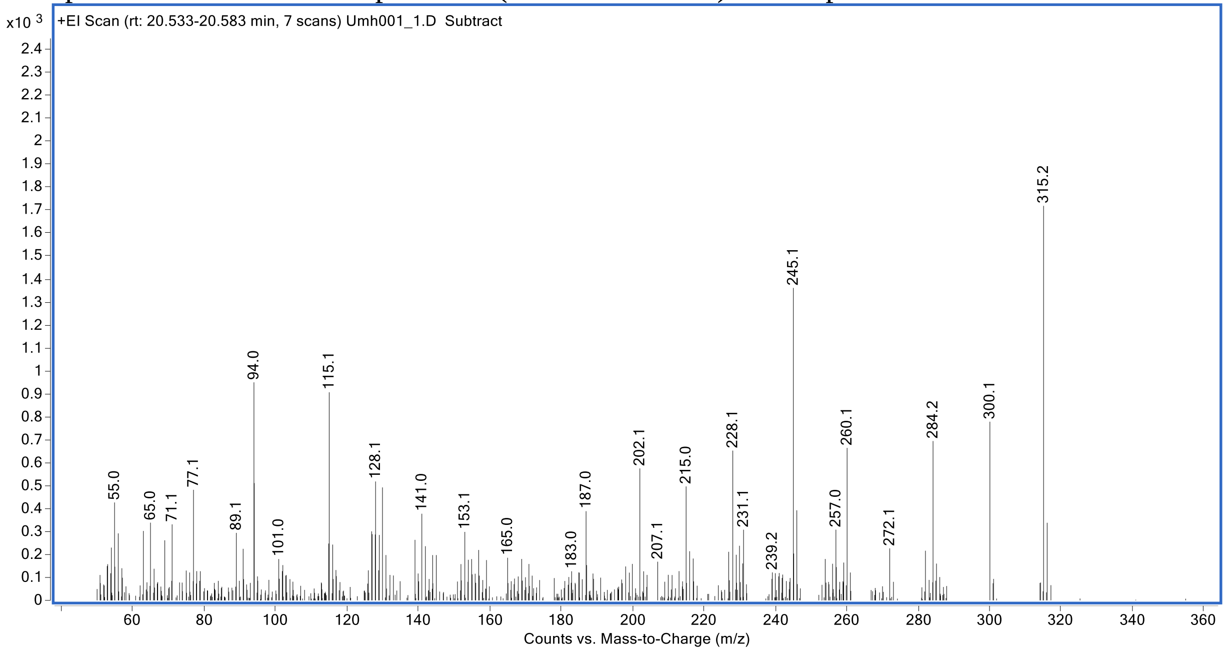

Data S2.

Ion chromatogram Sample 002 of characteristic ions for epibuphanisine, buphandrin, fatty acids and dehydroabietic acid (cf. Tab. S1). Compound acquisition time & match score: Myristic acid 13.897 min., 816; palmitic acid 15.818 min., 897; stearic acid 17.588 min., 838; buphandrin 20.549 min., 810.

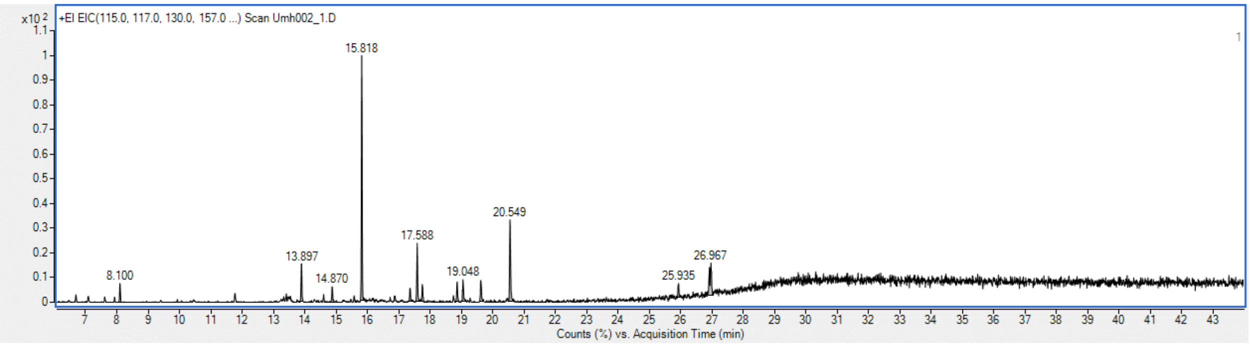

Mass spectrum identified as buphandrin (match score 810) in Sample 002.

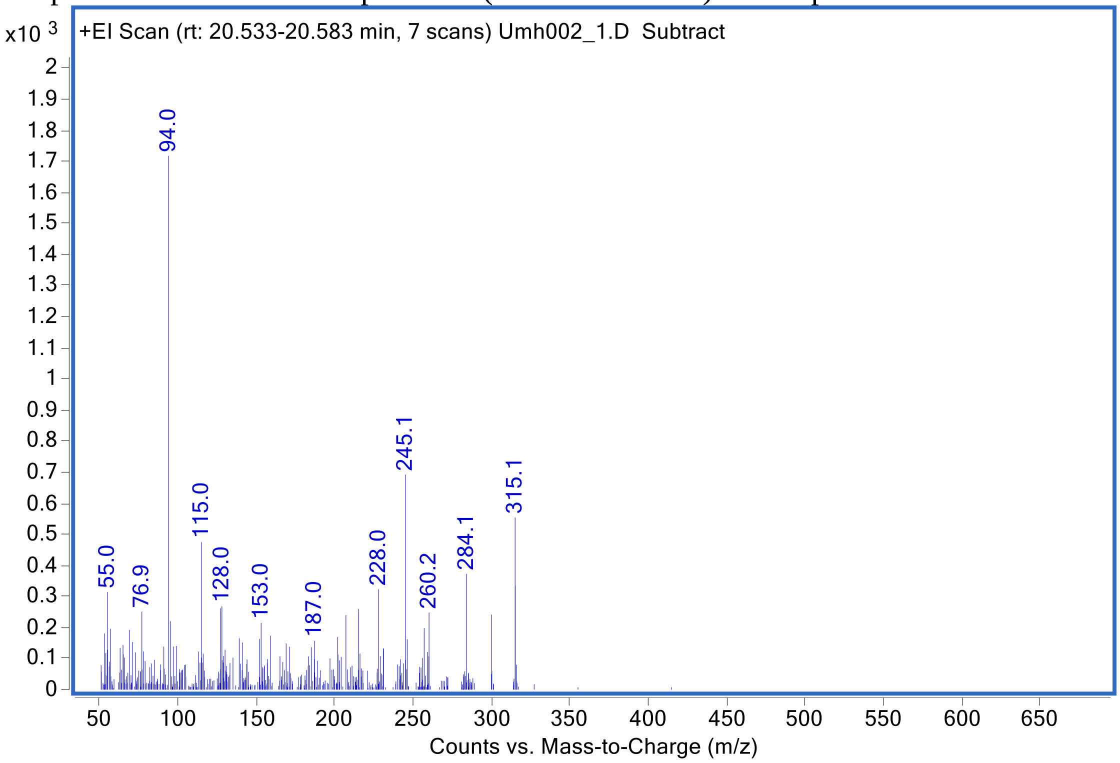

Data S3.

Ion chromatogram Sample 003 of characteristic ions for epibuphanisine, buphandrin, fatty acids and dehydroabietic acid (cf. Tab. S1). Compound acquisition time & match score: Palmitic acid 15.818 min., 861; buphandrin 20.558 min., 682.

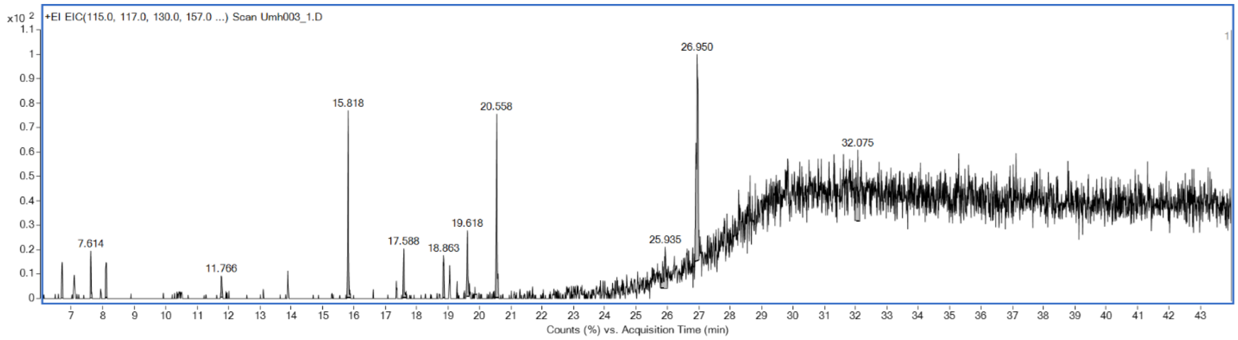

Mass spectrum identified as buphandrin (match score 682) in Sample 003.

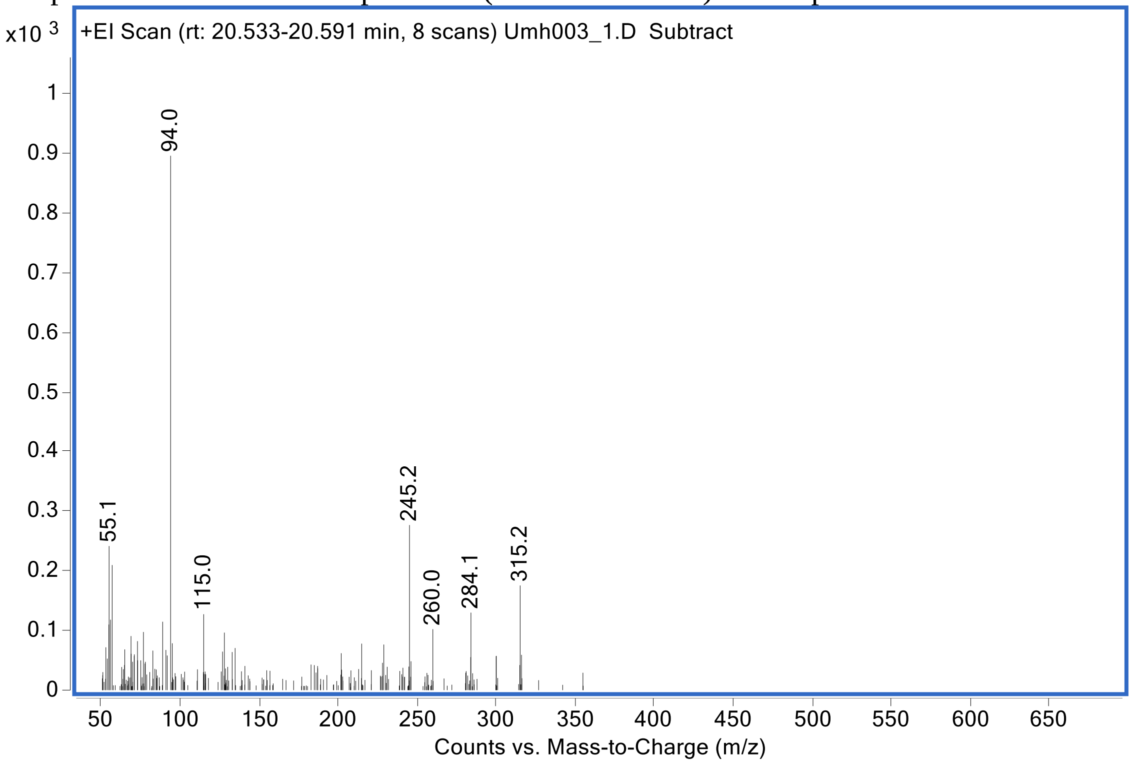

Data S4.

Ion chromatogram Sample 004 of characteristic ions for epibuphanisine, buphandrin, fatty acids and dehydroabietic acid (cf. Tab. S1). Compound acquisition time & match score: Palmitic acid 15.818 min., 839; stearic acid 17.588 min. 751; buphandrin 20.558 min., 683.

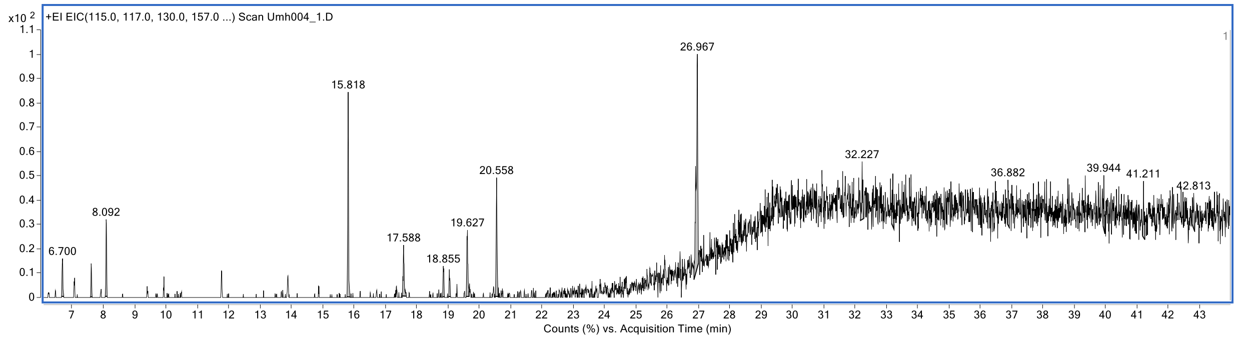

Mass spectrum identified as buphandrin (match score 683) in Sample 004.

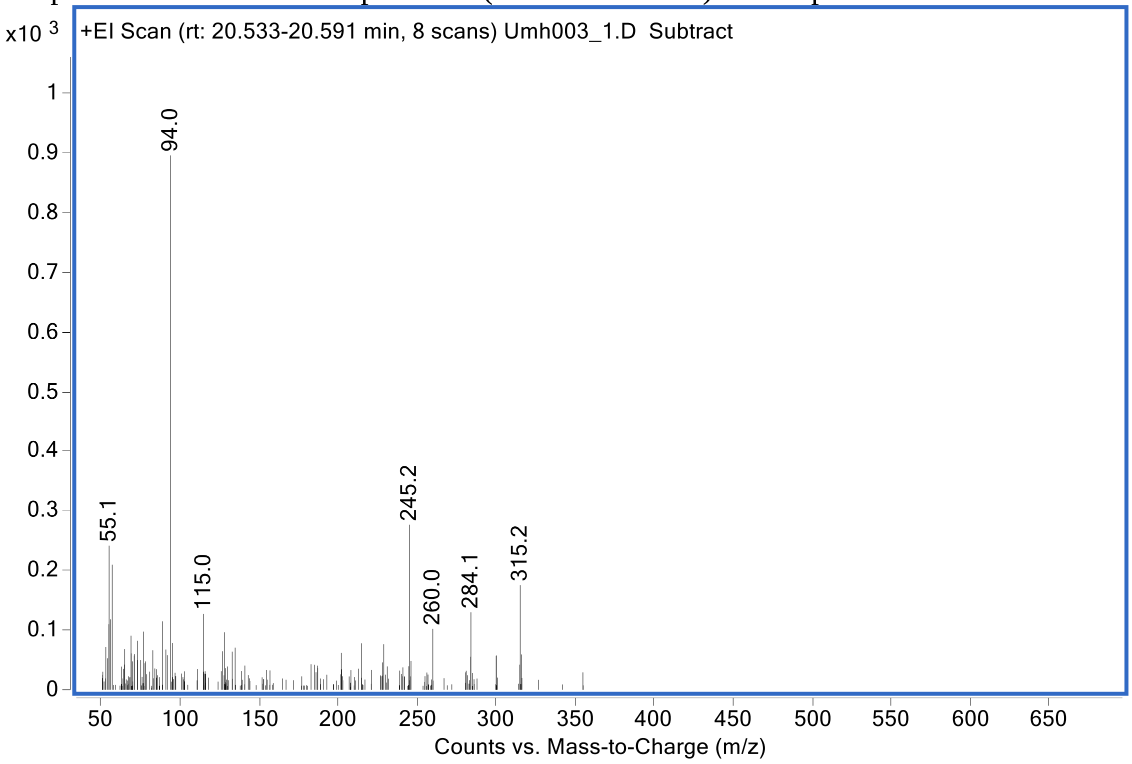

Data S5.

Ion chromatogram Sample 005 of characteristic ions for epibuphanisine, buphandrin, fatty acids and dehydroabietic acid (cf. Tab. S1). Compound acquisition time & match score: Palmitic acid 15.818 min., 875; stearic acid 17.580 min., 797; buphandrin 20.558 min., 729.

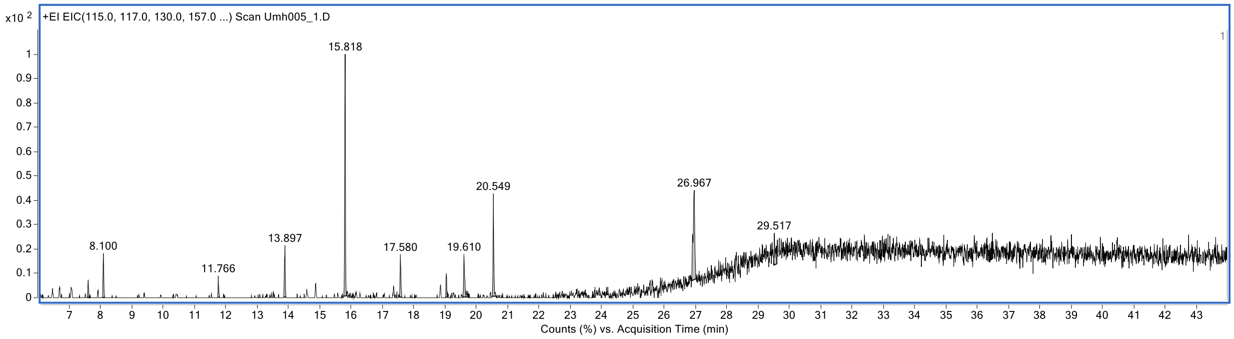

Mass spectrum identified as Buphandrin (match score 729) in Sample 005.

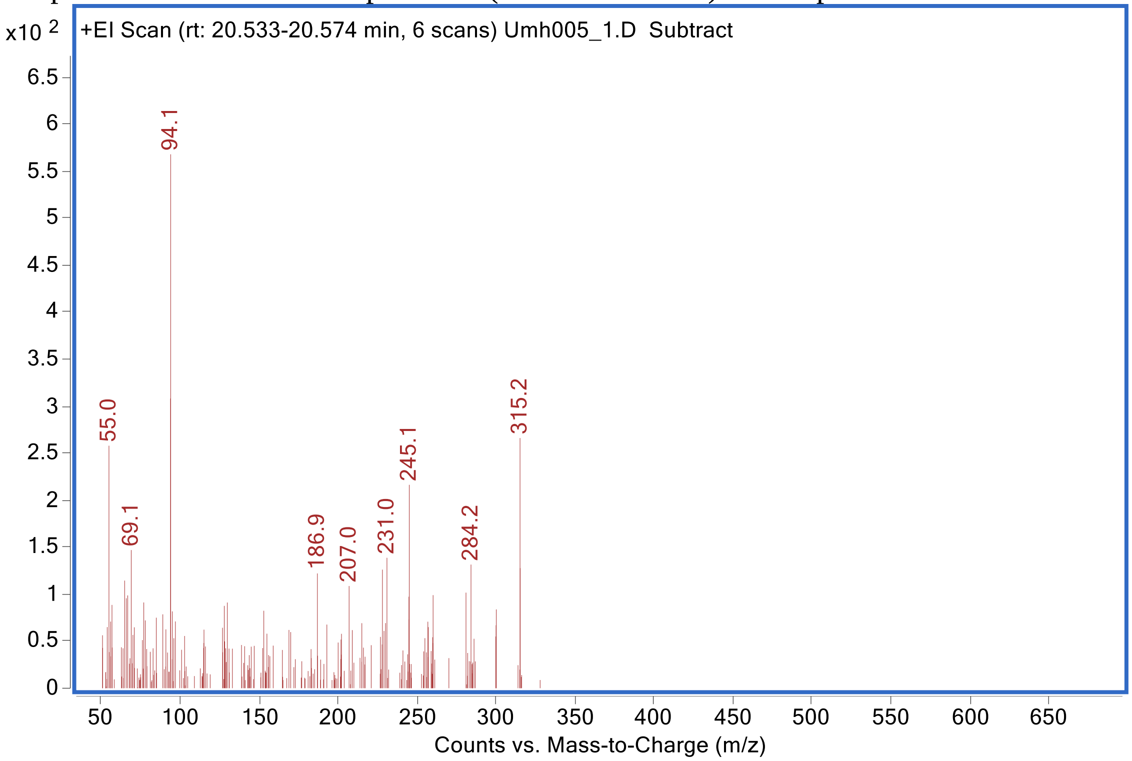

Data S6.

Ion chromatogram Sample 006 of characteristic ions for epibuphanisine, buphandrin, fatty acids and dehydroabietic acid (cf. Tab. S1). Compound acquisition time & match score: Palmitic acid 15.818 min., 875; stearic acid 17.580 min., 797. Peak at 20.549 min., match score < 600

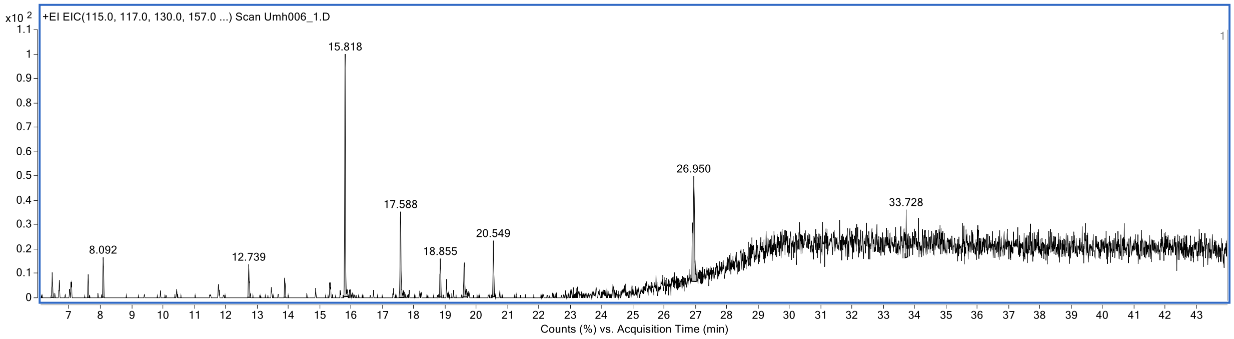

Data S7.

Ion chromatogram Sample 007 of characteristic ions for epibuphanisine, buphandrin, fatty acids and dehydroabietic acid (cf. Tab. S1). Compound acquisition time & match score: Palmitic acid 15.810 min., 882. All other peaks have match scores < 600.

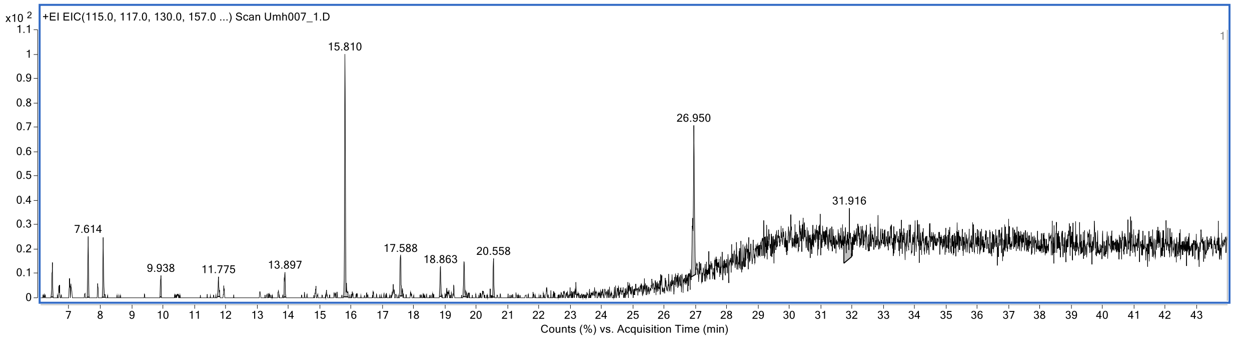

Data S8.

Ion chromatogram Sample 008 of characteristic ions for epibuphanisine, buphandrin, fatty acids and dehydroabietic acid (cf. Tab. S1). Compound acquisition time & match score: Palmitic acid 15.810 min., 882; stearic acid 17.580 min., 849. All other peaks have match scores < 600.

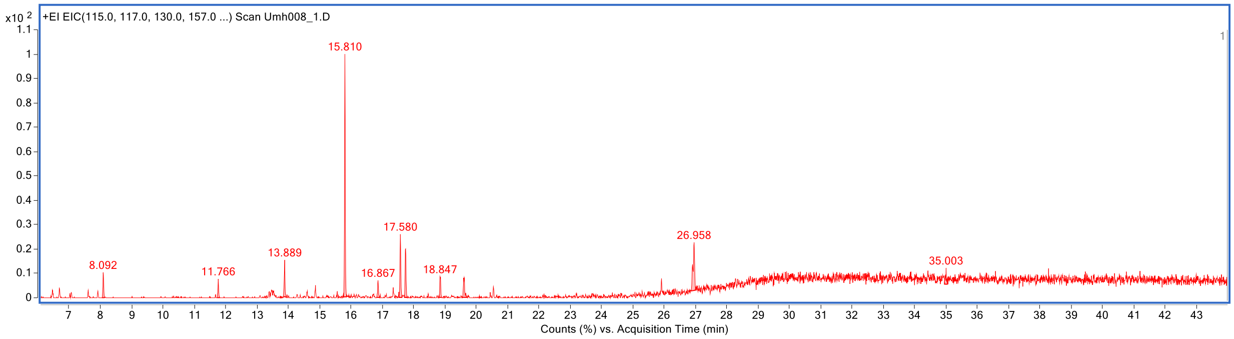

Data S9.

Ion chromatogram Sample 009 of characteristic ions for epibuphanisine, buphandrin, fatty acids and dehydroabietic acid (cf. Tab. S1). Compound acquisition time & match score: Palmitic acid 15.818 min., 863; stearic acid 17.588 min., 817. All other peaks have match scores < 600.

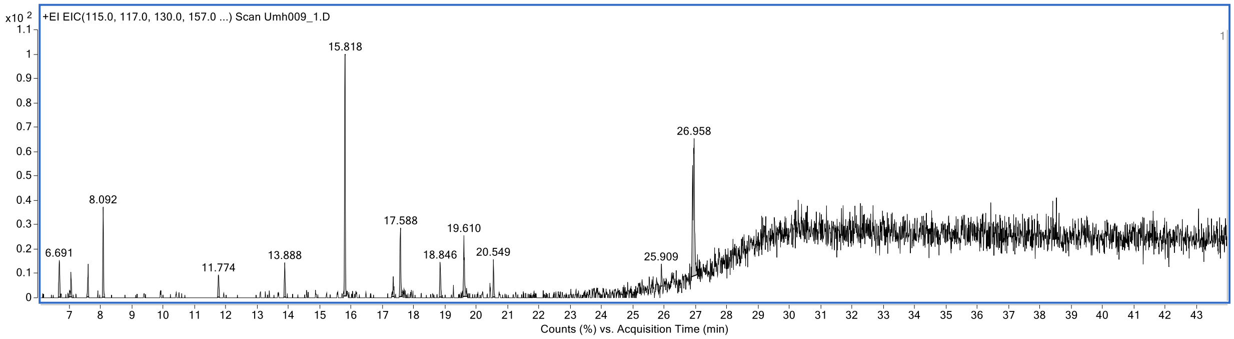

Data S10.

Ion chromatogram Sample 010 of characteristic ions for epibuphanisine, buphandrin, fatty acids and dehydroabietic acid (cf. Tab. S1). Compound acquisition time & match score: Palmitic acid 15.818 min., 834. All other peaks have match scores < 600.

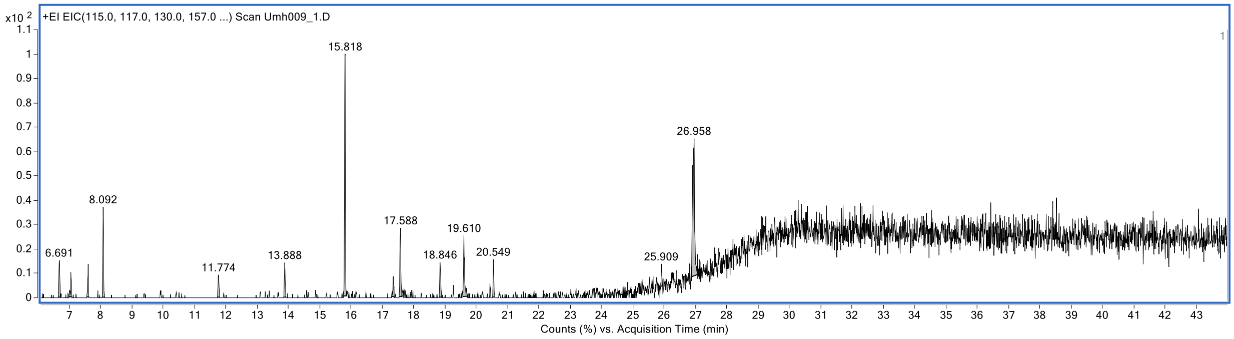

Data S11.
Total ion chromatogram of ethnographic sample 1799.02.75.k.

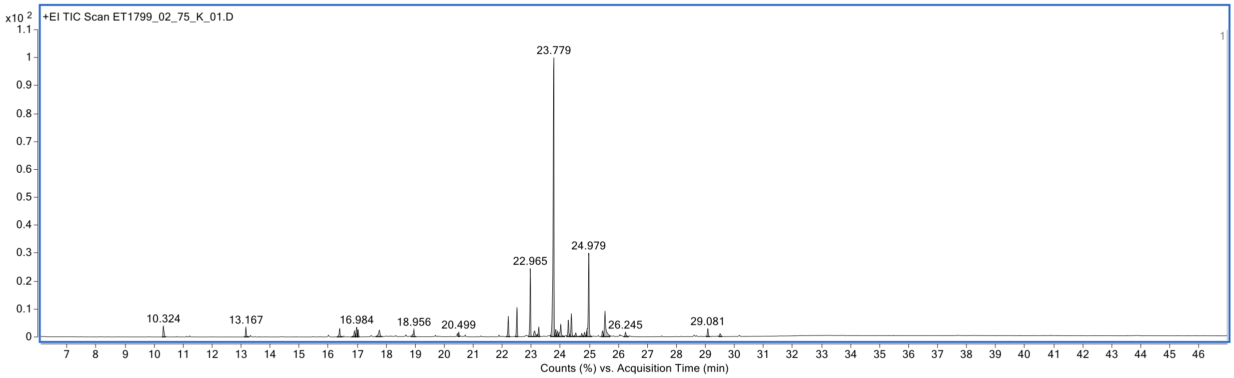

Mass spectrum identified as epibuphanisine (acquisition time 22.210 min., match score 893) sample 1799.02.75.k.

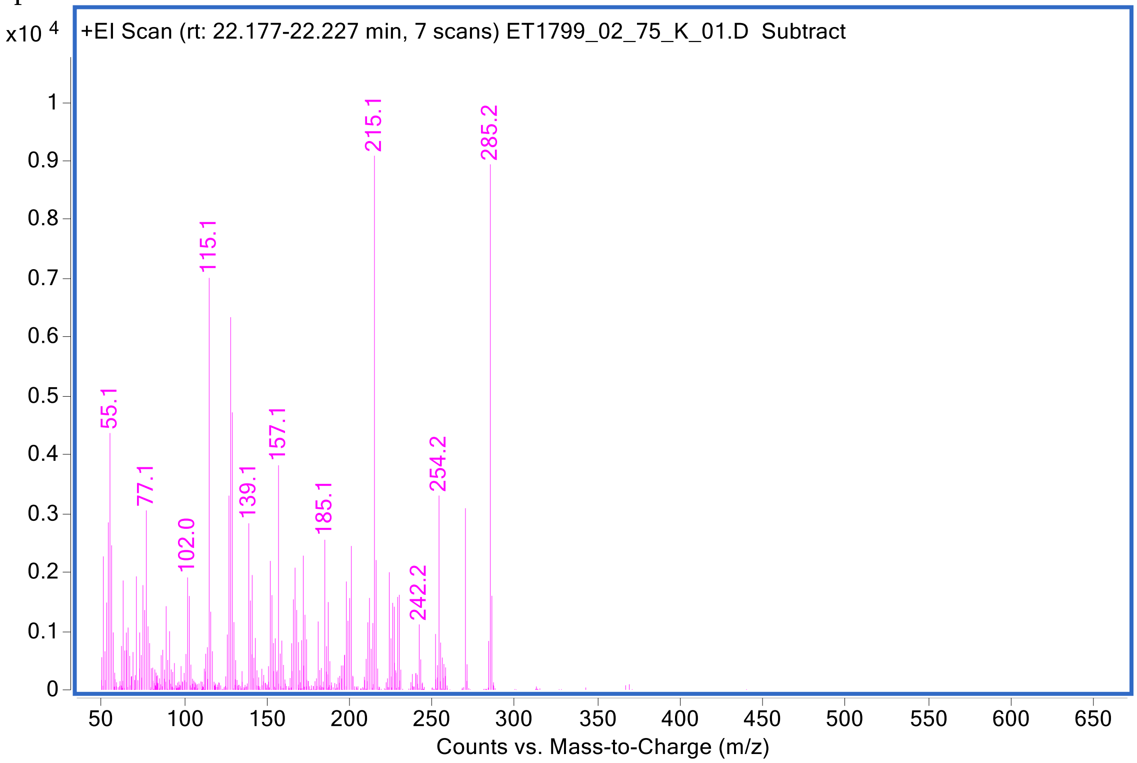

Mass spectrum identified as buphandrin (acquisition time 23.779 min., match score 897) sample 1799.02.75.k.

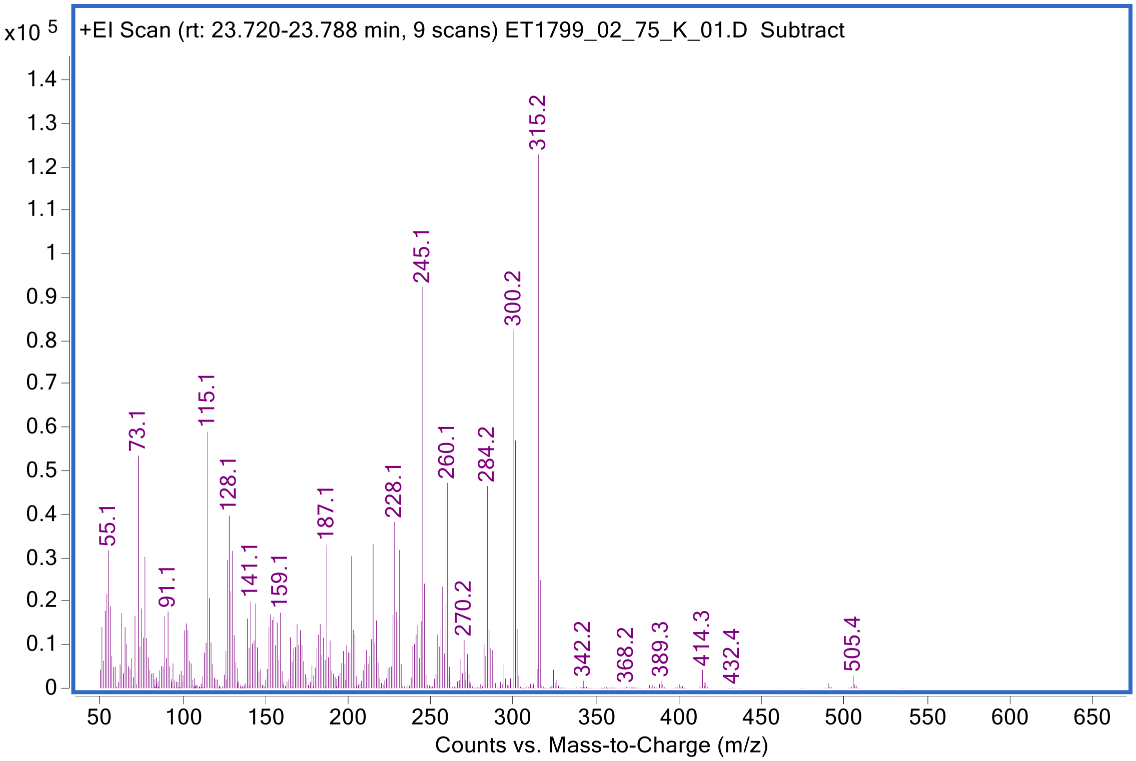

x10 5
+EI Scan (rt: 23.720-23.788 min, 9 scans) ET1799_02_75_K_01.D Subtract
1.4
1.3
1.2
1.1
1
0.9
0.8
0.7
0.6
0.5
0.4
0.3
0.2
0.1
0
55.1
73.1
91.1
115.1
128.1
141.1
159.1
187.1
228.1
245.1
260.1
270.2
284.2
300.2
315.2
342.2
368.2
389.3
414.3
432.4
505.4
50 100 150 200 250 300 350 400 450 500 550 600 650
Counts vs. Mass-to-Charge (m/z)

Data S12.

Total ion chromatogram of ethnographic sample 1799.02.75.m.

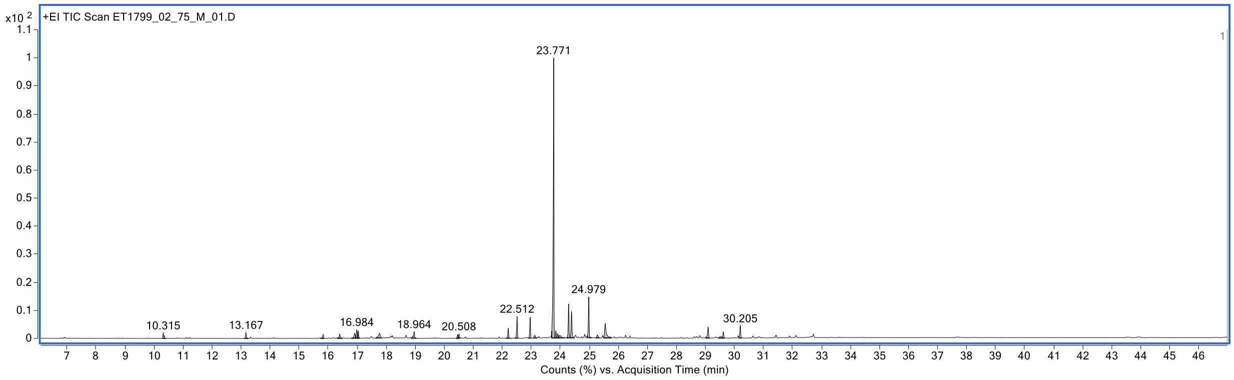

Mass spectrum identified as epibuphanisine (acquisition time 22.210 min., match score 869) sample 1799.02.75.m.

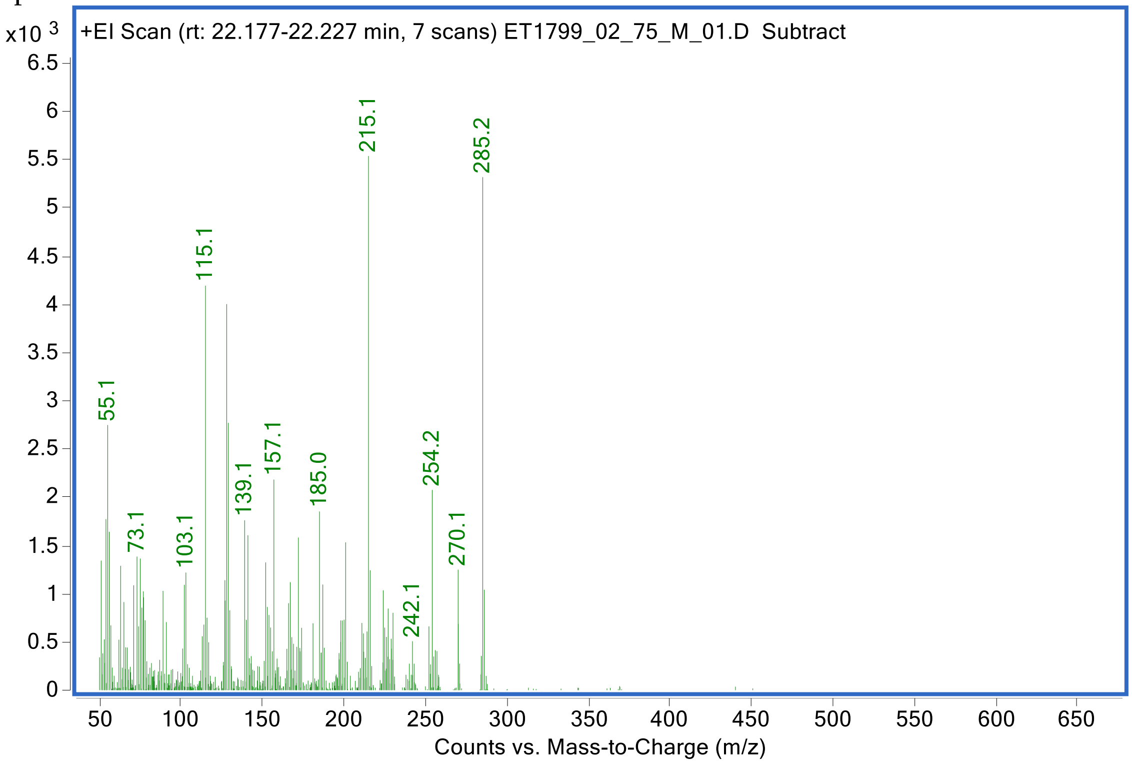

Mass spectrum identified as buphandrin (acquisition time 23.771 min., match score 842) sample 1799.02.75.m.

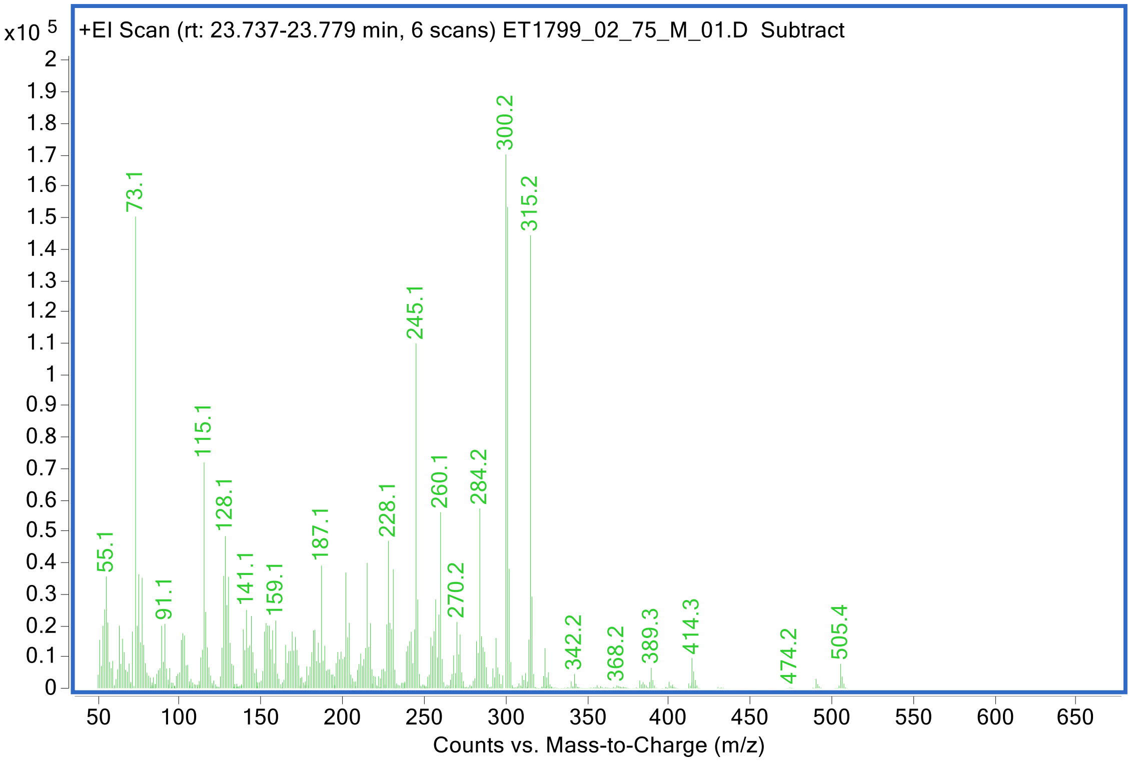
+EI Scan (rt: 23.737-23.779 min, 6 scans) ET1799_02_75_M_01.D Subtract
x10 5
2
1.9
1.8
1.7
1.6
1.5
1.4
1.3
1.2
1.1
1
0.9
0.8
0.7
0.6
0.5
0.4
0.3
0.2
0.1
0
55.1
73.1
91.1
115.1
128.1
141.1
159.1
187.1
228.1
245.1
260.1
270.2
284.2
300.2
315.2
342.2
368.2
389.3
414.3
474.2
505.4
50
100
150
200
250
300
350
400
450
500
550
600
650
Counts vs. Mass-to-Charge (m/z)

Data S13.

Total ion chromatogram of ethnographic sample 1874.01.251.c.

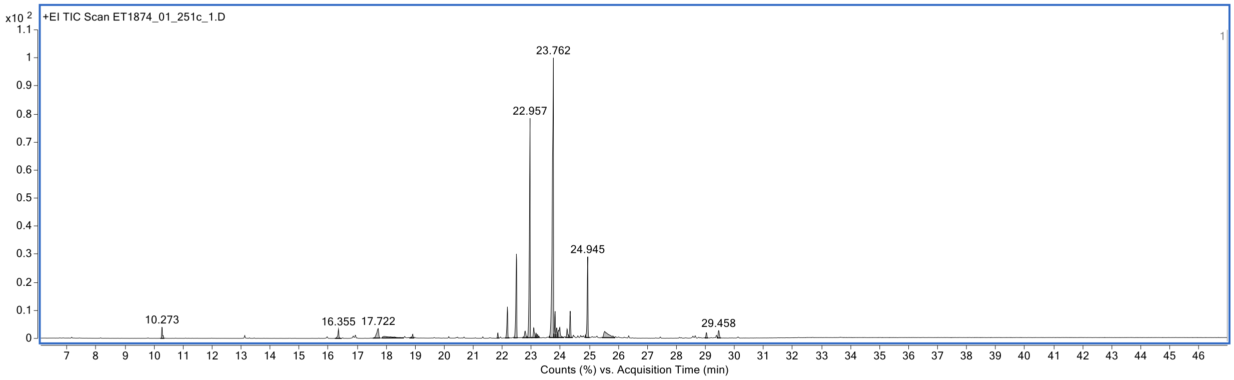

Mass spectrum identified as epibuphanisine (acquisition time 22.177 min., match score 889) sample 1874.01.251.c.

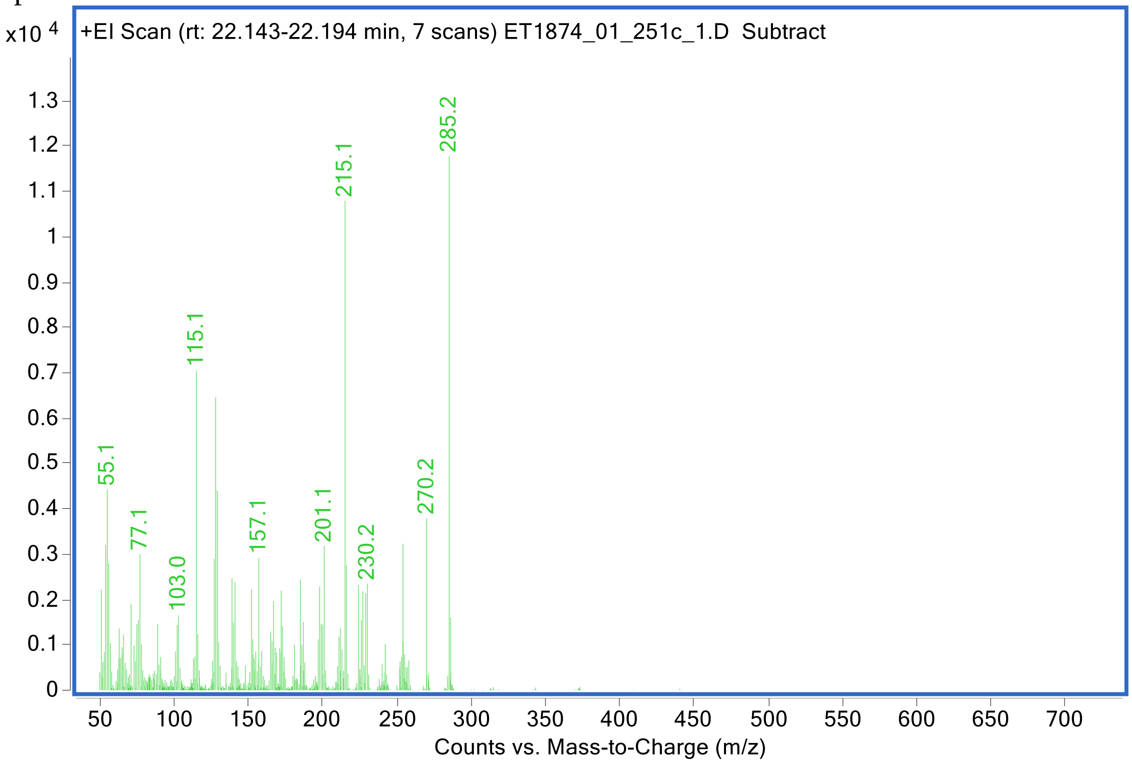

Mass spectrum identified as buphandrin (acquisition time 23.762 min., match score 902) sample 1874.01.251.c.

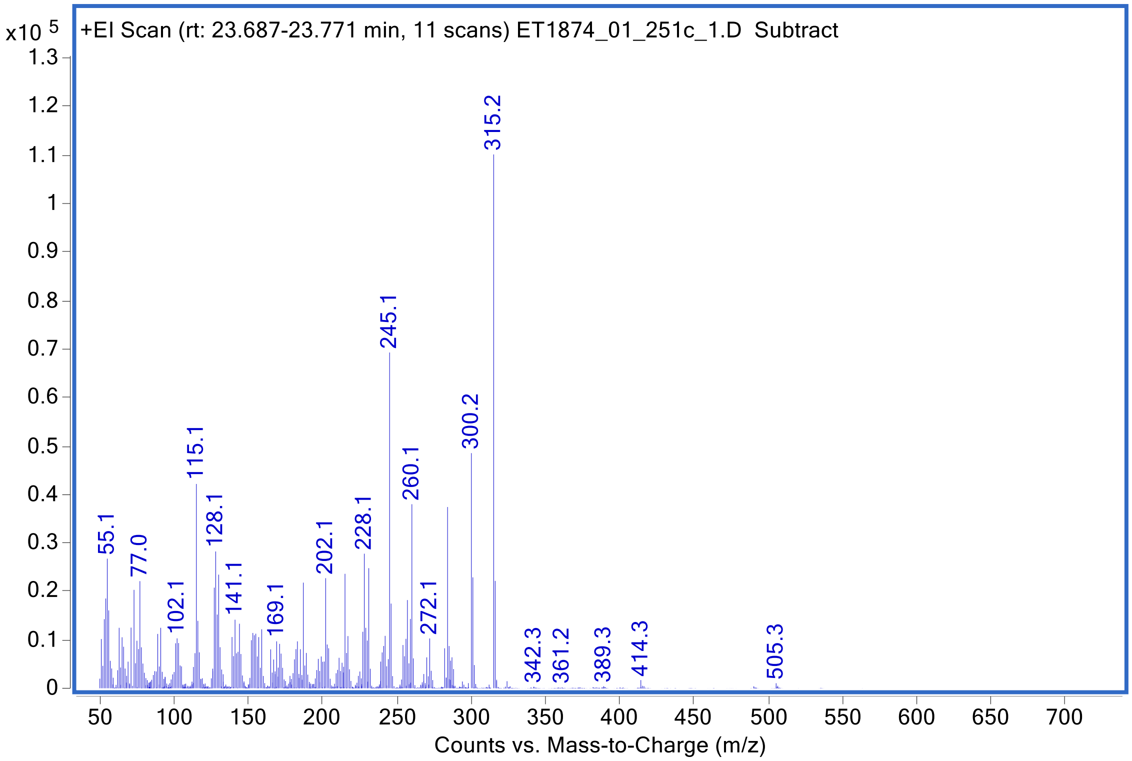
x10 5
+EI Scan (rt: 23.687-23.771 min, 11 scans) ET1874_01_251c_1.D Subtract
1.3
1.2
1.1
1
0.9
0.8
0.7
0.6
0.5
0.4
0.3
0.2
0.1
0
55.1
77.0
102.1
115.1
128.1
141.1
169.1
202.1
228.1
245.1
260.1
272.1
300.2
315.2
342.3
361.2
389.3
414.3
505.3
50
100
150
200
250
300
350
400
450
500
550
600
650
700
Counts vs. Mass-to-Charge (m/z)

Data S14.

Total ion chromatogram of ethnographic sample 1874.01.251.d.

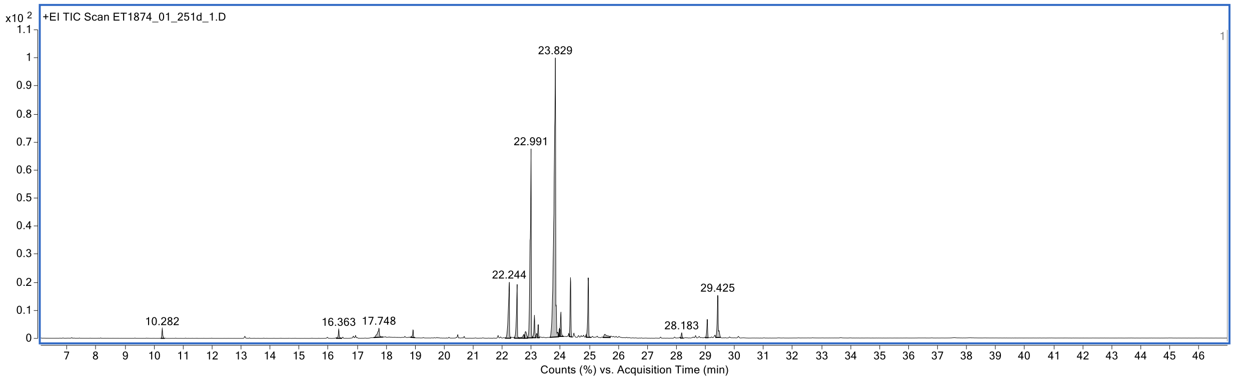

Mass spectrum identified as epibuphanisine (acquisition time 22.244 min., match score 895) sample 1874.01.251.d.

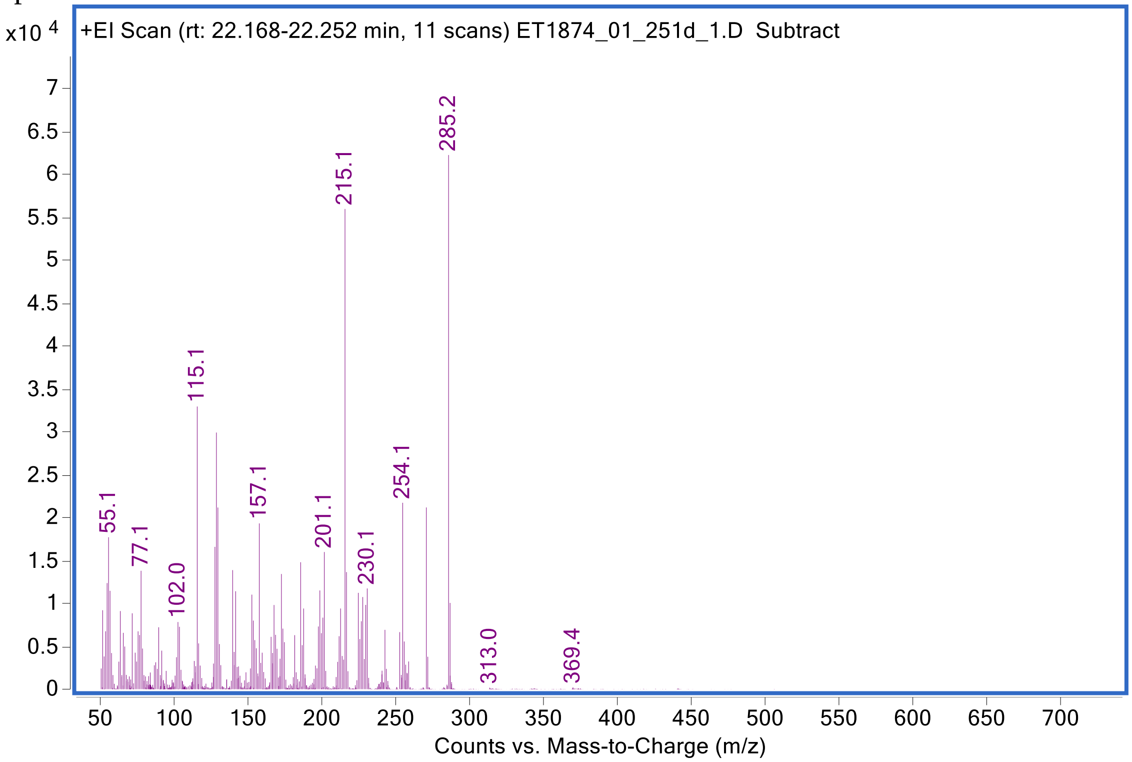

Mass spectrum identified as buphandrin (acquisition time 23.829 min., match score 900) sample 1874.01.251.d.

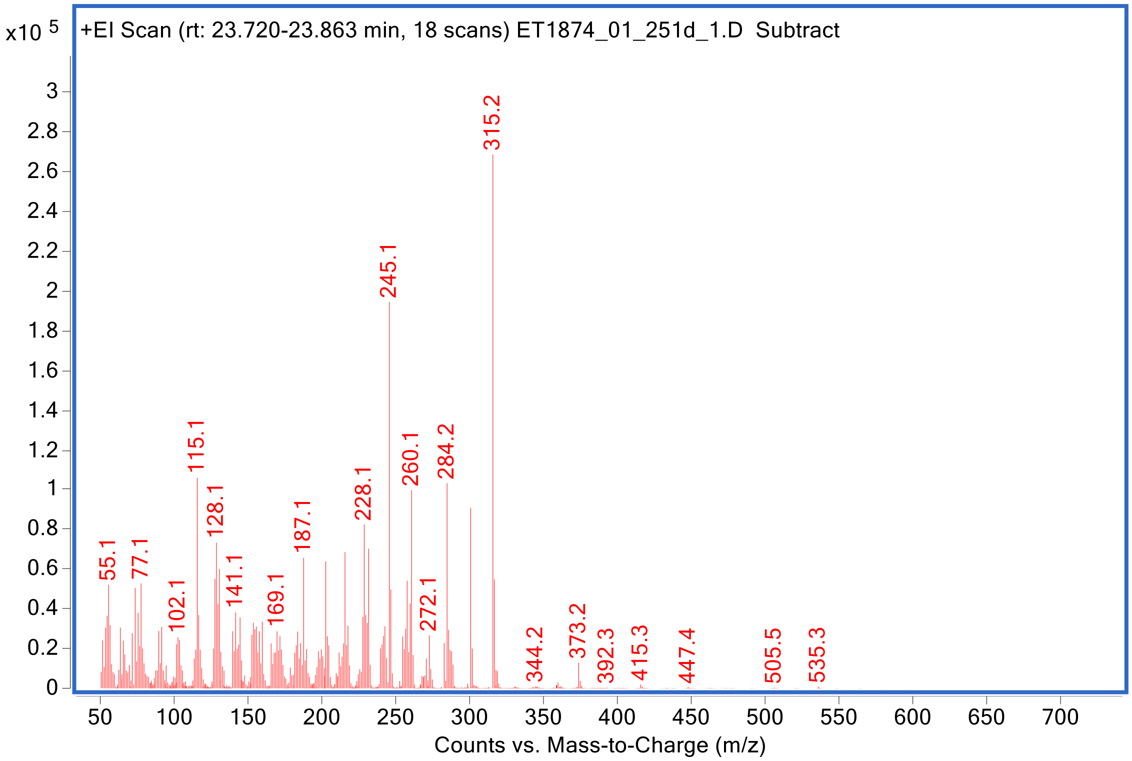

Data S15.

Ion chromatogram ethnographic sample 1799.02.75.k of characteristic ions for epibuphanisine, buphandrin, fatty acids and dehydroabietic acid (cf. Tab. S1). Compound acquisition time & match score: Epibuphanisine 18.964 min., 804; buphandrin 20.474 min., 887.

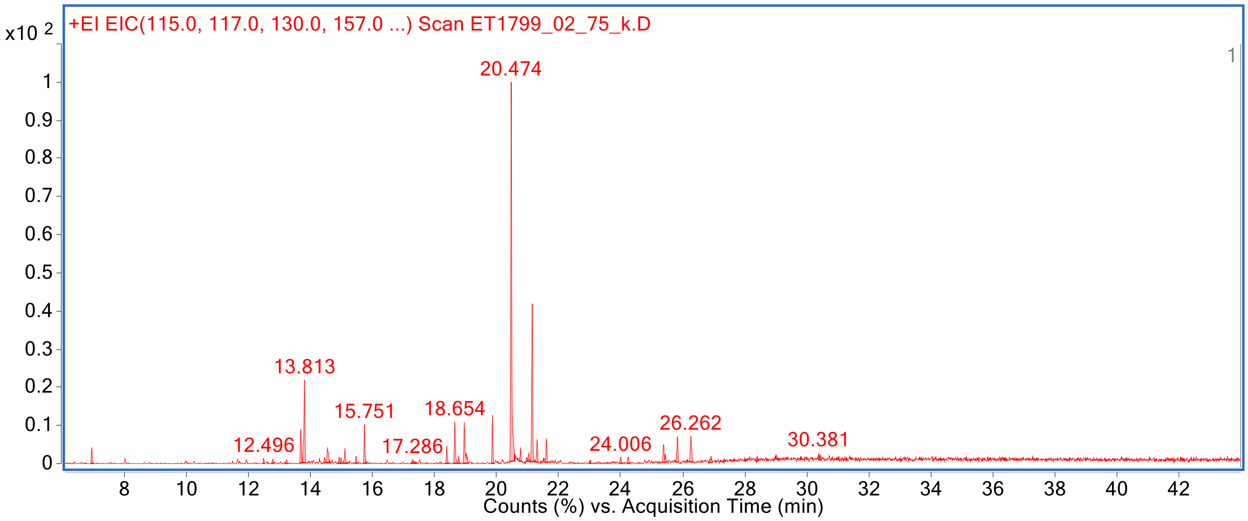

Mass spectrum identified as epibuphanisine (match score 804) in ethnographic sample 1799.02.75.k.

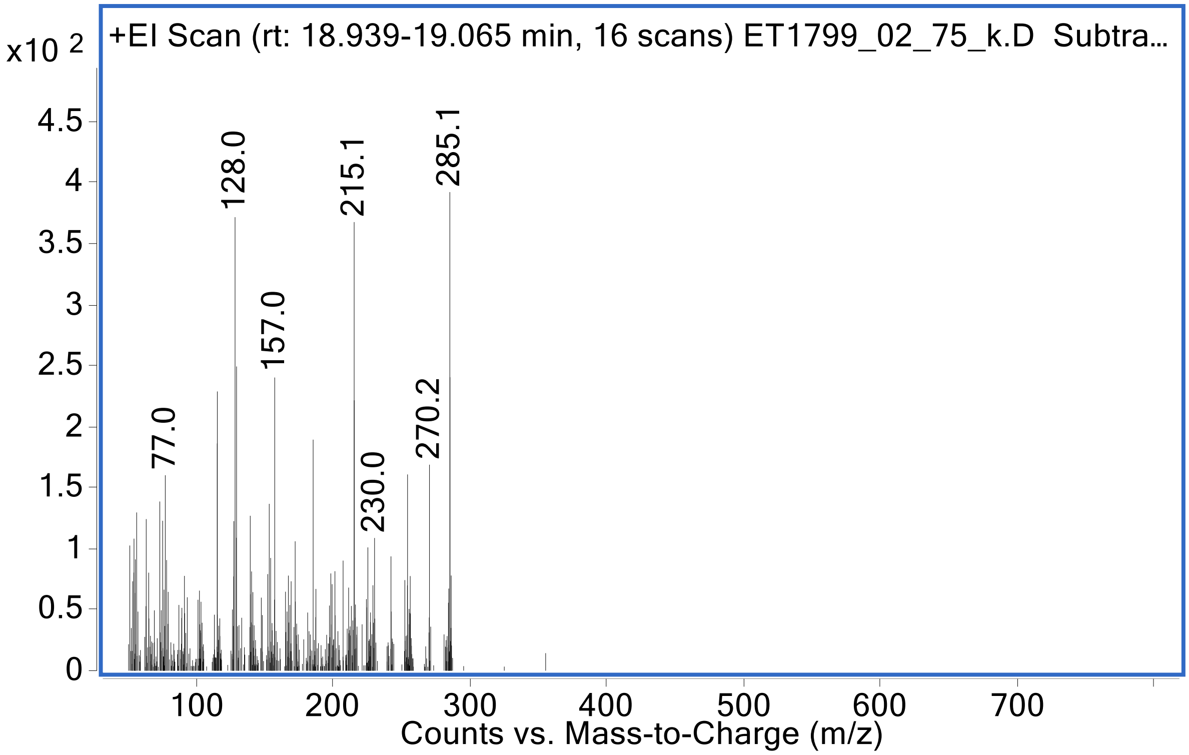

Mass spectrum identified as buphandrin (match score 887) in ethnographic sample 1799.02.75.k.

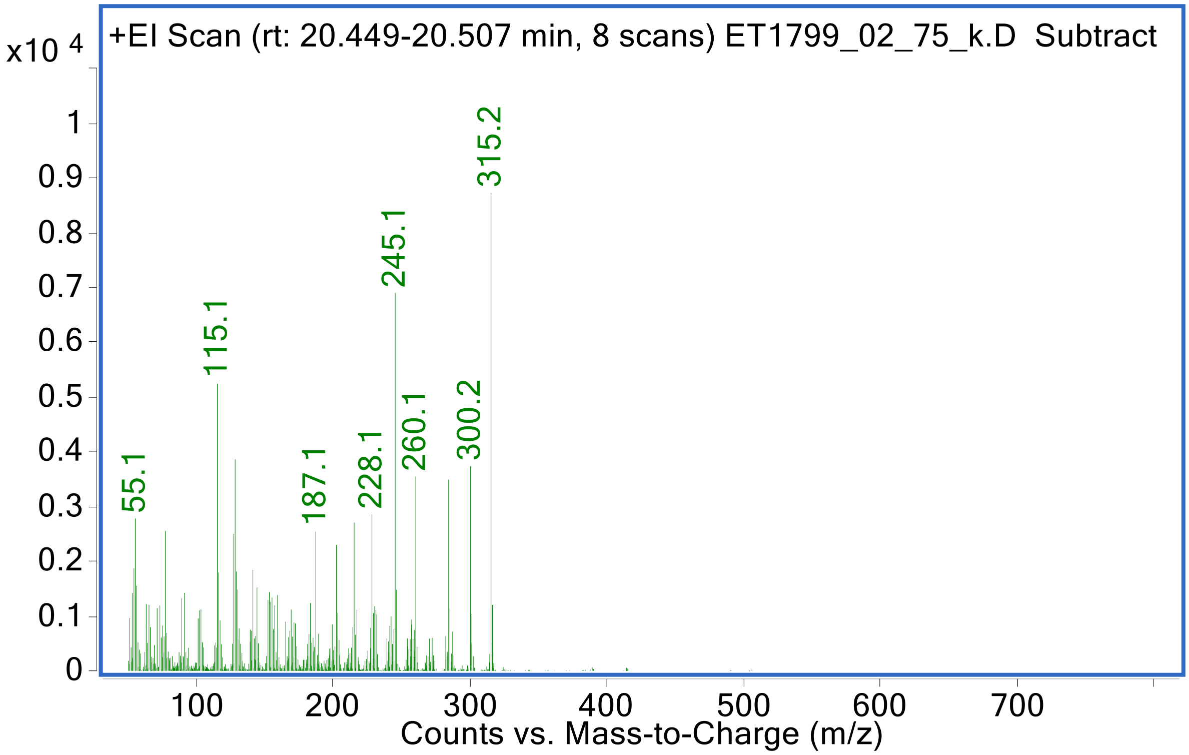

Data S16.

Ion chromatogram ethnographic sample 1799.02.75.m of characteristic ions for epibuphanisine, buphandrin, fatty acids and dehydroabietic acid (cf. Tab. S1). Compound acquisition time & match score: Epibuphanisine 18.956 min., 798; buphandrin 20.474 min., 890.

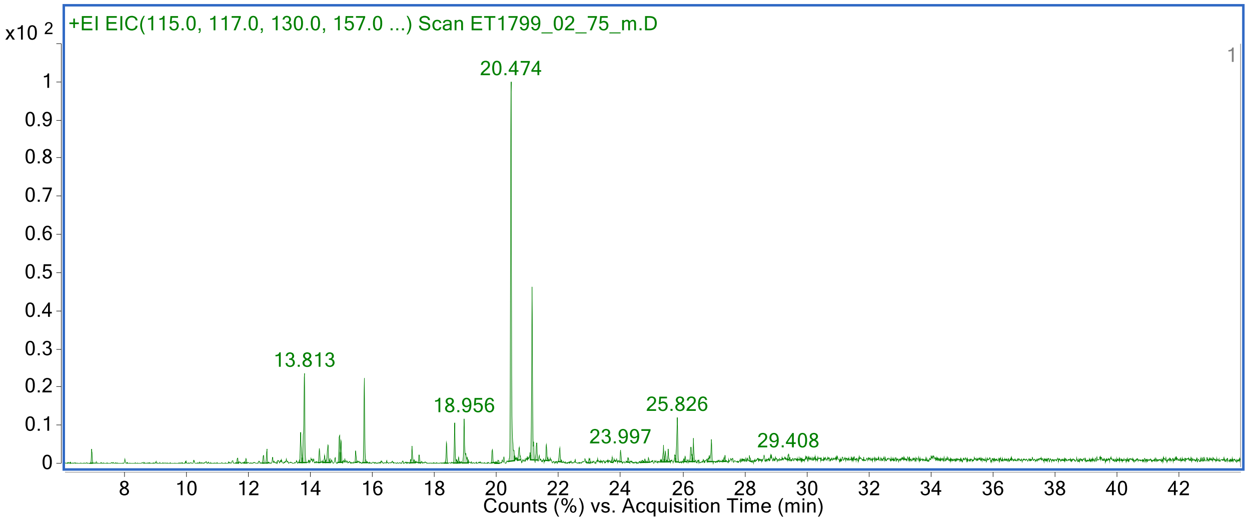

Mass spectrum identified as epibuphanisine (match score 798) in ethnographic sample 1799.02.75.m.

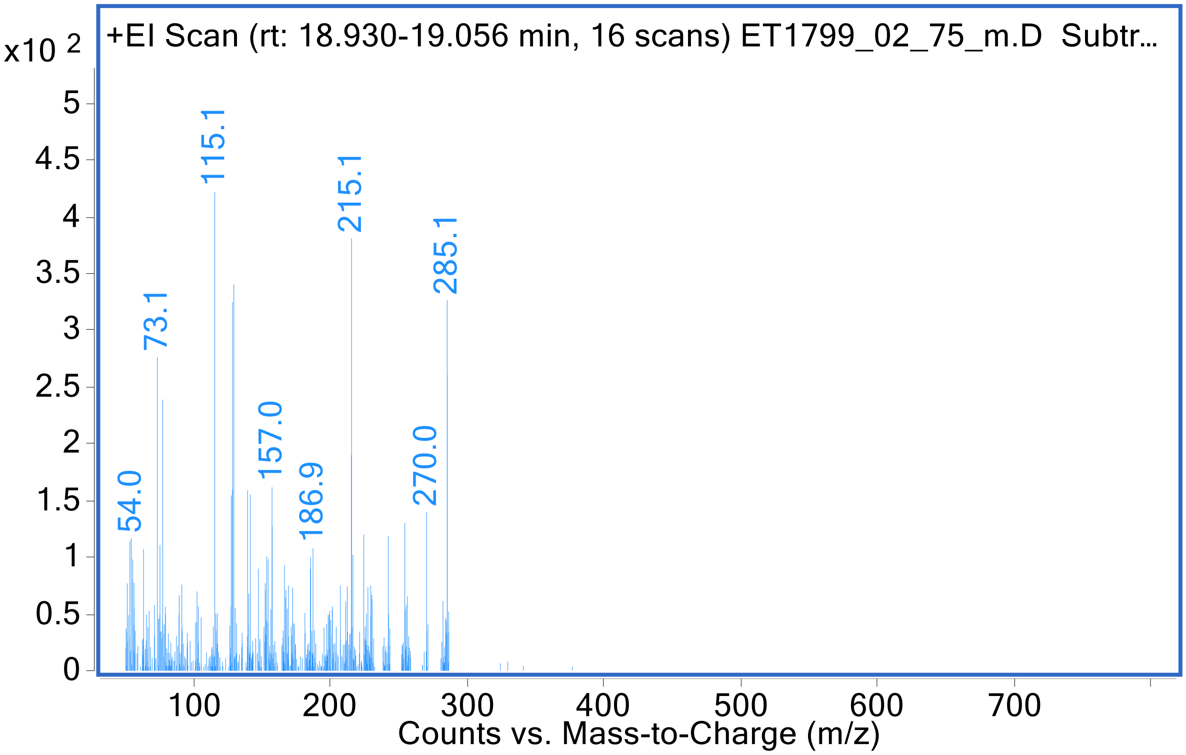

Mass spectrum identified as buphandrin (match score 890) in ethnographic sample 1799.02.75.m.

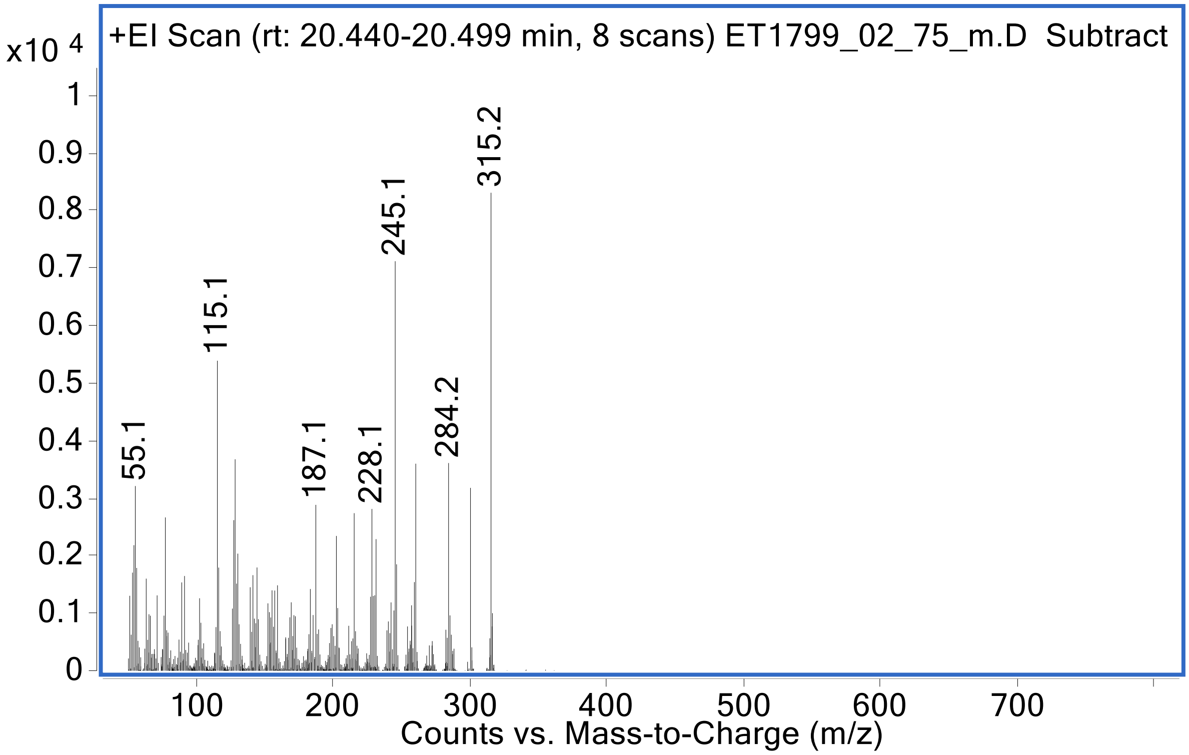

Data S17.
Ion chromatogram ethnographic sample 1874.01.251.c of characteristic ions for epibuphanisine, buphandrin, fatty acids and dehydroabietic acid (cf. Tab. S1). Compound acquisition time & match score: epibuphanisine 18.956 min., 798; Buphandrin 20.474 min., 890.

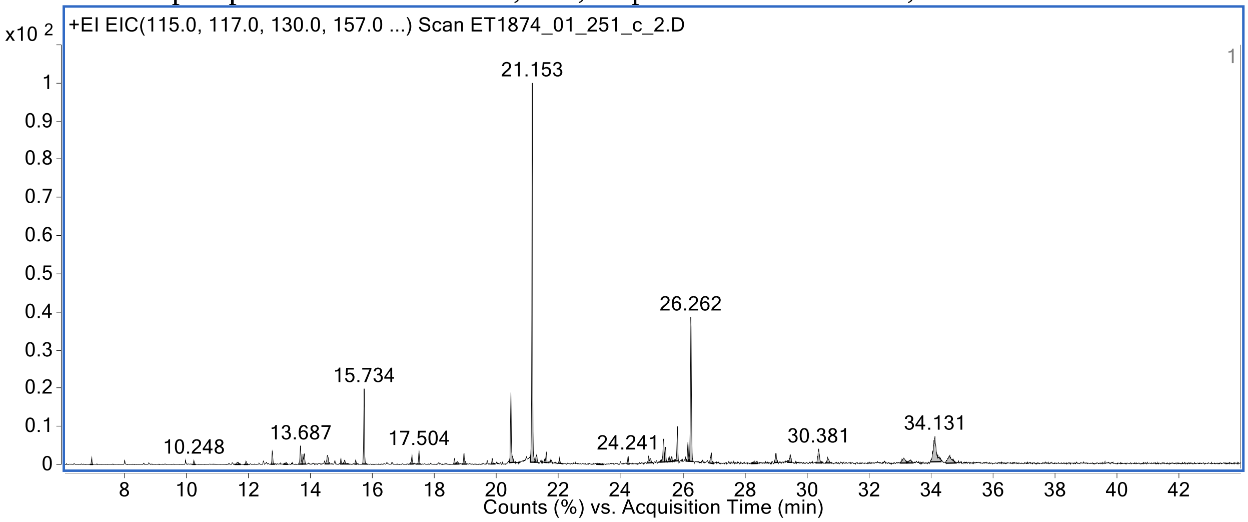

Mass spectrum identified as epibuphanisine (match score 798) in ethnographic sample 1874.01.251.c.

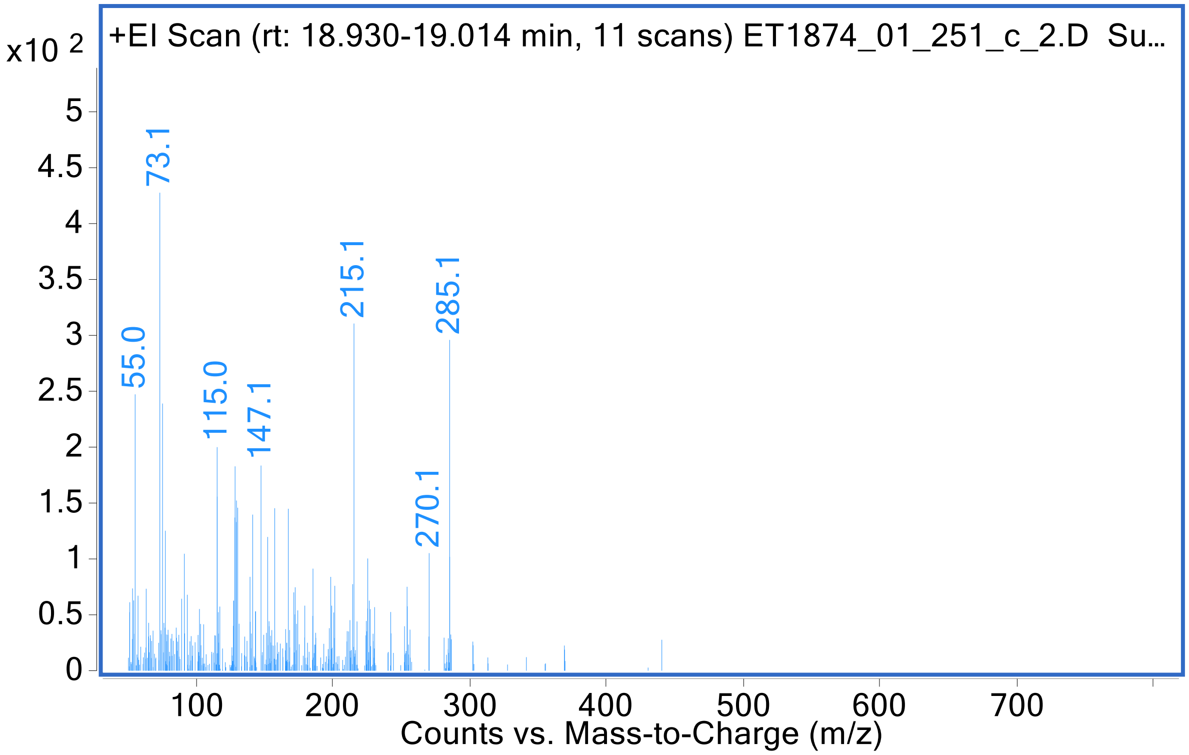

Mass spectrum identified as buphandrin (match score 890) in ethnographic sample 1874.01.251.c.

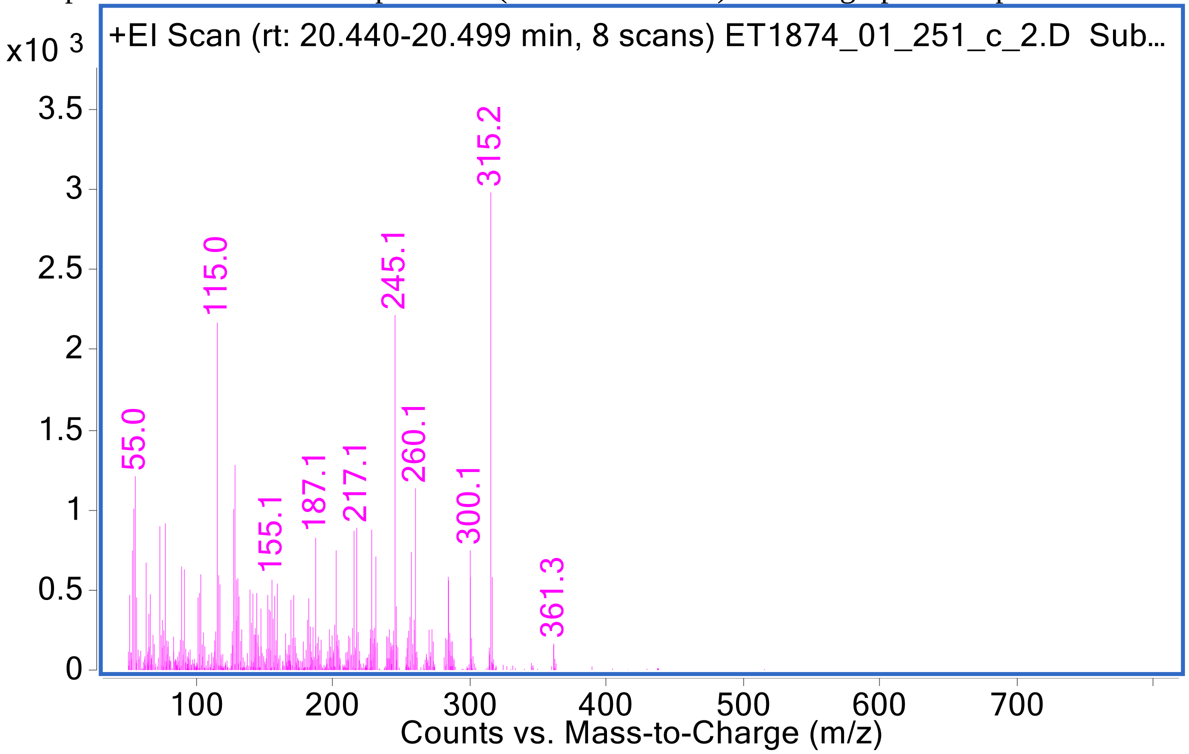

Data S18.

Ion chromatogram ethnographic sample 1874.01.251.d of characteristic ions for epibuphanisine, buphandrin, fatty acids and dehydroabietic acid (cf. Tab. S1). Compound acquisition time & match score: buphandrin 20.466 min., 818.

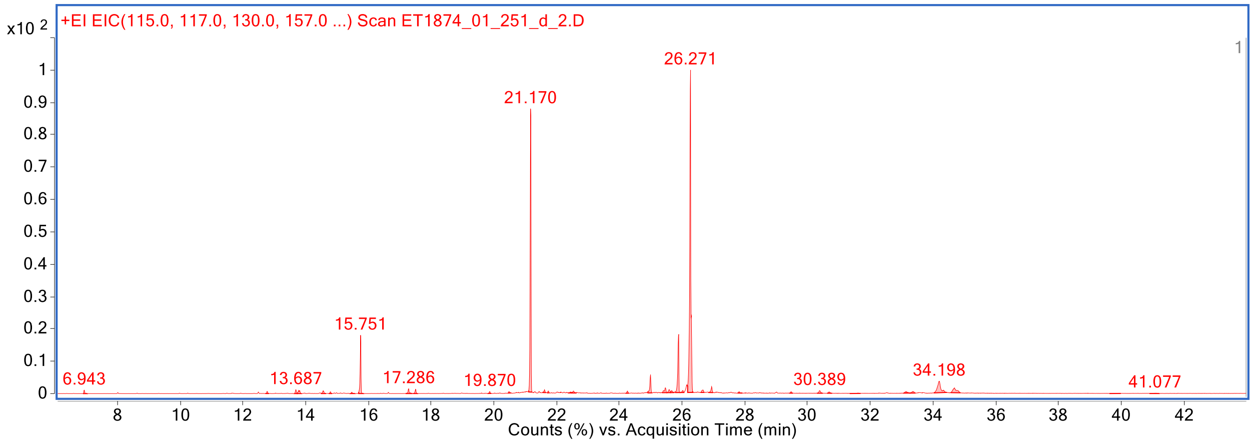

Mass spectrum identified as buphandrin (match score 887) in ethnographic sample 1874.01.251.d.

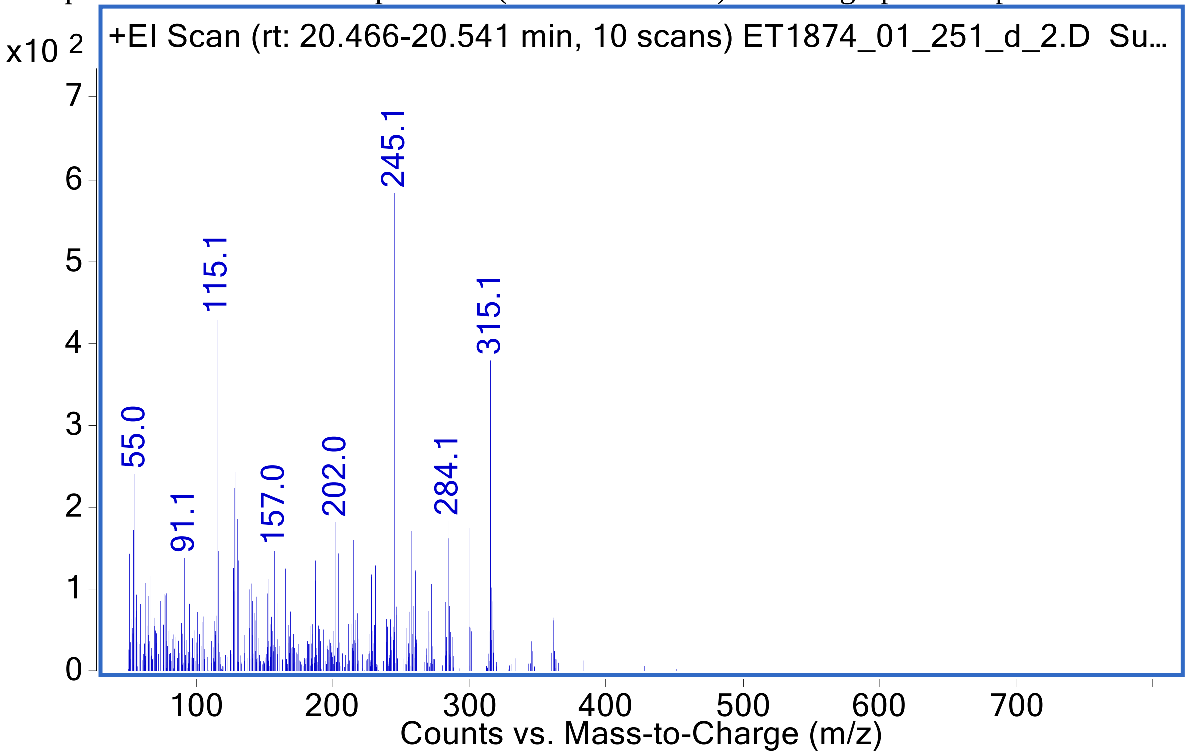

Data S19.

Bulb of *Boophone distichia* (Bergius Botanic Garden, Stockholm, Sweden). Ion chromatogram of characteristic ions for epibuphanisine, buphandrin, fatty acids and dehydroabietic acid (cf. Tab. S1). Compound acquisition time & match score: Epibuphanisine 18.947 min, 882; buphandrin 20.508 min., 920.

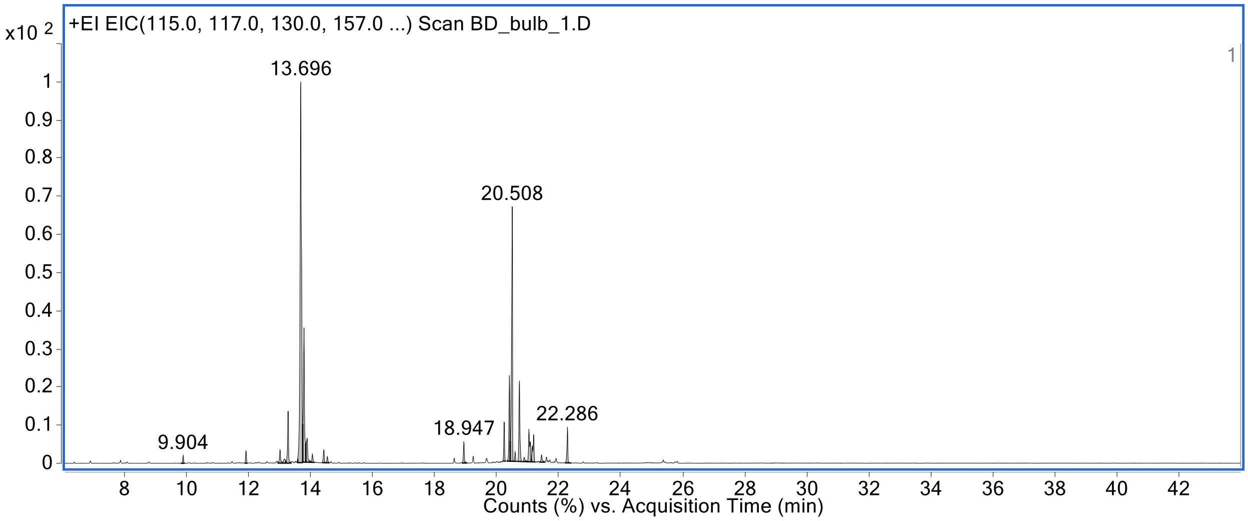

Mass spectrum identified as epibuphanisine (match score 882) in authentic sample of bulb of *Boophone distichia* (Bergius Botanic Garden, Stockholm, Sweden).

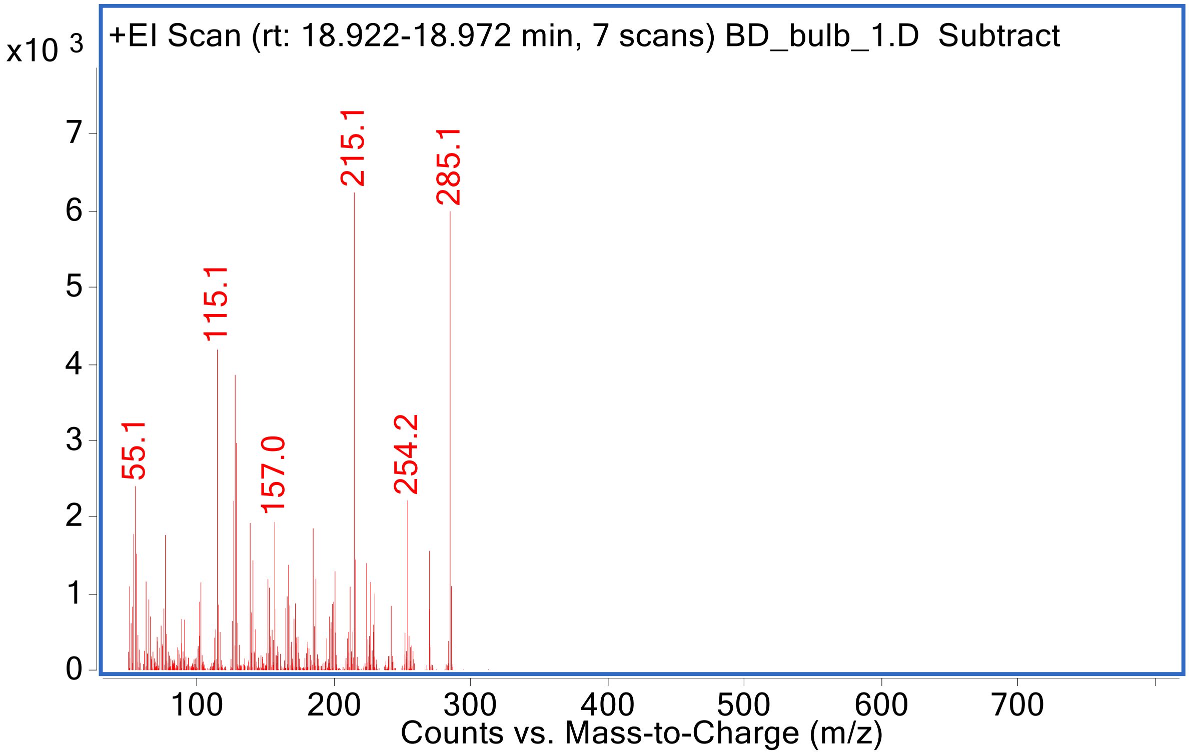

Mass spectrum identified as buphandrin (match score 920) in authentic sample of bulb of *Boophone distichia* (Bergius Botanic Garden, Stockholm, Sweden).

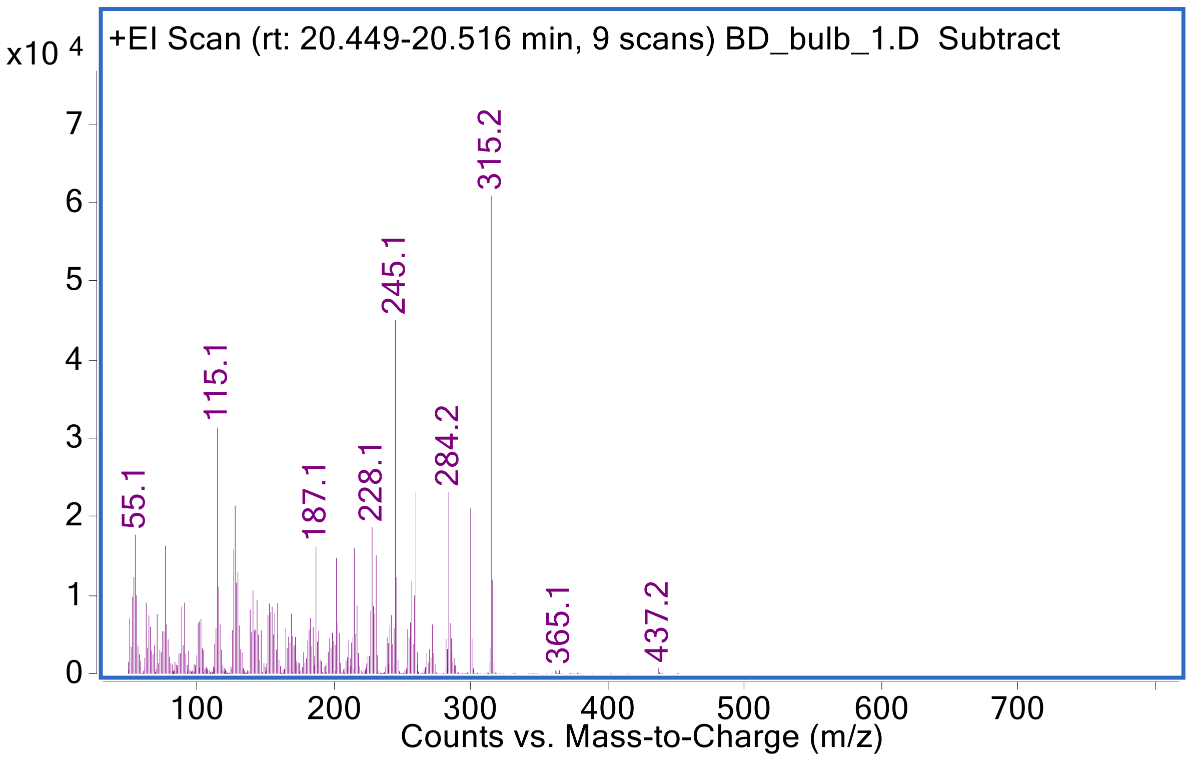
+EI Scan (rt: 20.449-20.516 min, 9 scans) BD_bulb_1.D Subtract
x10 4
0
1
2
3
4
5
6
7
55.1
115.1
187.1
228.1
245.1
284.2
315.2
365.1
437.2
100
200
300
400
500
600
700
Counts vs. Mass-to-Charge (m/z)

REFERENCES

1. C. E. Finch, C. B. Stanford, Meat-adaptive genes and the evolution of slower aging in humans. *Q. Rev. Biol.* **79**, 3–50 (2004).

2. L. Backwell, J. Bradfield, K. J. Carlson, T. Jashashvili, L. Wadley, F. d'Errico, The antiquity of bow-and-arrow technology: Evidence from Middle Stone Age layers at Sibudu Cave. *Antiquity* **92**, 289–303 (2018).

3. J. Bradfield, M. Lombard, J. Reynard, A. Wurz, Further evidence for bow hunting and its implications more than 60 000 years ago: Results of a use-trace analysis of the bone point from Klasies River Main site, South Africa. *Quat. Sci. Rev.* **236**, 106295 (2020).

4. H.D. Neuwinger, *African ethnobotany: Poisons and drugs: Chemistry*, *pharmacology*, *toxicology* (CRC Press, 1996).

5. M. Lombard, From complex techno-behaviour to complex attention through the genes of the precuneus. *J. Archaeol. Method Theory* **32**, e46 (2025).

6. M. Shaw, P. Woolley, F. Rae, Bushmen arrow poisons. *Cimbebasia* **7**, 2–41 (1963).

7. L. Lewin, Untersuchungen über Buphane disticha (*Haemanthus toxicarius*). *Archiv f. experiment. Pathol. u. Pharmakol.* **68**, 333–340 (1912).

8. S. Isaksson, A. Högberg, M. Lombard, J. Bradfield, Potential biomarkers for southern African hunter-gatherer arrow poisons applied to ethno-historical and archaeological samples. *Sci. Rep.* **13**, 11877 (2023).

9. J. D. Clark, J. L. Phillips, P. S. Staley, *Interpretations of prehistoric technology from ancient Egyptian and other Sources. Part I: Ancient Egyptian bows and arrows and their relevance for African prehistory*. *Paléorient* **2**, 323–388 (1974).

10. J. Bradfield, I. A. Dubery, P. A. Steenkamp, A 7,000-year-old multi-component arrow poison from Kruger Cave, South Africa. *iScience* **27**, 111438 (2024).

11. F. d'Errico, L. Backwell, P. Villa, I. Degano, J. J. Lucejko, M. K. Bamford, T. F. G. Higham, M. P. Colombini, P. B. Beaumont, Early evidence of San material culture represented by organic artifacts from Border Cave, South Africa. *Proc. Natl. Acad. Sci. U.S.A.* **109**, 13214–13219 (2012).

12. J. N. F. Binneman, A unique stone-tipped arrowhead from Adam's Kranz Cave, Eastern Cape. *South. Afr. field archaeol.* **3**, 58–60 (1994).

13. M. M. Lahr, F. Rivera, R. K. Power, A. Mounier, B. Copsey, F. Crivellaro, J. E. Edung, J. M. Fernandez, C. Kiarie, J. Lawrence, A. Leakey, E. Mbua, H. Miller, A. Muigai, D. M. Mukhongo, A. van Baelen, R. Wood, J.-L. Schwenninger, R. Grün, H. Achyuthan, A. Wilshaw, R. A. Foley, Inter-group violence among early Holocene hunter-gatherers of West Turkana, Kenya. *Nature* **529**, 394–398 (2016).

14. S. T. Goldstein, C. M. Shaffer, Experimental and archaeological investigations of backed microlith function among mid-to-late Holocene herders in southwestern Kenya. *Archaeol. Anthropol. Sci.* **9**, 1767–1788 (2017).

15. M. Lombard, Quartz-tipped arrows older than 60 ka: Further use-trace evidence from Sibudu, KwaZulu-Natal, South Africa. *J. Archaeol. Sci.* **38**, 1918–1930 (2011).

16. P. de la Peña, N. Taipale, L. Wadley, V. Rots, A techno-functional perspective on quartz micro-notches in Sibudu's Howiesons Poort indicates the use of barbs in hunting technology. *J. Archaeol. Sci.* **93**, 166–195 (2018).

17. D. W. Phillipson, Some speculations on the beginning of backed-microlith manufacture, in *Proceedings of the Eighth Pan-African Congress of Prehistory and Quaternary Studies*. B. A. Ogot, R. E. Leakey, Eds., 229–230. (Nairobi: The International Louis Leakey Memorial Institute for Prehistory and Paleontology, 1980).

18. S. H. Ambrose, Small things remembered: Origins of early microlithic industries in sub-Saharan Africa. *Archeol. Pap. Am. Anthropol. Assoc.* **12**, 9–29 (2002).

19. J. Pargeter, J. J. Shea, Going big versus going small: Lithic miniaturization in hominin lithic technology. *Evol. Anthropol. Issues News Rev.* **28**, 72–85 (2019).

20. M. Lombard, L. Wadley, Z. Jacobs, M. Mohapi, R. G. Roberts, Still Bay and serrated points from Umhlatuzana rock shelter, KwaZulu-Natal, South Africa. *J. Archaeol. Sci.* **37**, 1773–1784 (2010).

21. M. Lombard, L. Phillipson, Indications of bow and stone-tipped arrow use 64 000 years ago in KwaZulu-Natal, South Africa. *Antiquity* **84**, 635–648 (2010).

22. M. Lombard, The gripping nature of ochre: The association of ochre with Howiesons Poort adhesives and Later Stone Age mastics from South Africa. *J. Hum. Evol.* **53**, 406–419 (2007).

23. M. Lombard, Testing for poisoned arrows in the Middle Stone Age: A tip cross-sectional analysis of backed microliths from southern Africa. *J. Archaeol. Sci. Rep.* **34**, 102630 (2020).

24. F. H. Reidsma, I. Sifogeorgaki, A. Dinckal, H. Huisman, M. J. Sier, B. van Os, G. L. Dusseldorp, Making the invisible stratigraphy visible: A grid-based, multi-proxy geoarchaeological study of Umhlatuzana Rockshelter, South Africa. *Front. Earth Sci.* **9**, 664105 (2021).

25. I. Sifogeorgaki, H. Huisman, P. Karkanas, V. C. Schmid, G. L. Dusseldorp, Sand, hearths, lithics and a bit of bioturbation: Site formation processes at Umhlatuzana rockshelter, South Africa. *Geoarchaeology* **39**, 212–237 (2024).

26. J. Kaplan, The Umhlatuzana rock shelter sequence: 100 000 years of Stone Age history. *South. Afr. Humanit.* **2**, 1–94 (1990).

27. I. Sifogeorgaki, G. L. Dusseldorp, True colours: Analysing apparent sedimentary divisions in the stratigraphic sequence of Umhlatuzana Rockshelter (South Africa) using Munsell-based colour determinations. *South. Afr. field archaeol.* **17**, e1329 (2023).

28. A. Fischer, P. V. Hansen, P. Rasmussen, Macro and micro wear traces on lithic projectile points. *J. Dan. Archaeol.* **3**, 19–46 (1984).

29. A. Yaroshevich, D. Kaufman, D. Nuzhnyy, O. Bar-Yosef, M. Weinstein-Evron, Design and performance of microlith implemented projectiles during the Middle and the Late Epipaleolithic of the Levant: Experimental and archaeological evidence. *J. Archaeol. Sci.* **37**, 368–388 (2010).

30. P. Villa, L. L. Pollarolo, I. Degano, L. Birolo, M. Pasero, C. Biagioni, K. Douka, R. Vinciguerra, J. J. Lucejko, L. Wadley, A milk and ochre paint mixture used 49,000 years ago at Sibudu, South Africa. *PLOS ONE* **10**, e0131273 (2015).

31. A. Casoli, P. C. Musini, G. Palla, Gas chromatographic-mass spectrometric approach to the problem of characterizing binding media in paintings. *J. Chromatogr. A* **731**, 237–246 (1996).

32. R. Chasan, M.-A. Veall, L. I. Baron, A. Aleo, P. R. B. Kozowyk, G. H. J. Langejans, Podocarpaceae and Cupressaceae: A tale of two conifers and ancient adhesives production in South Africa. *PLOS ONE* **19**, e0306402 (2024).

33. H. J. Deacon, Excavations at Boomplaas Cave: A sequence through the upper Pleistocene and Holocene in South Africa. *World Archaeol.* **10**, 241–257 (1979).

34. M. Steyn, J. Binneman, M. Loots, The Kouga mummified human remains. *S. Afr. Archaeol. Bull.* **62**, 3–8 (2007).

35. J. Bradfield, S. Woodborne, J. Hollmann, I. Dubery, A 500-year-old medicine container discovered near Misgund, Eastern Cape, South Africa: Residue characterisation by GC-MS. *S. Afr. J. Sci.* **119**, 1–8 (2023).

36. C. P. Thunberg, *Travels in Europe*, *Africa and Asia made between the years 1772 and 1779*, *Volume II: Containing two expeditions to the interior part of the country adjacent to the Cape of Good Hope and a voyage to the island of Java*, *performed in the years 1773*, *1774*, *and 1775*. Second Edition. (London: Printed for F. & C. Rivington and sold by W. Richardson, 1795).

37. J. J. Nair, J. Van Staden, Traditional usage, phytochemistry and pharmacology of the South African medicinal plant *Boophone disticha* (L.f.) Herb. (Amaryllidaceae). *J. Ethnopharmacol.* **151**, 12–26 (2014).

38. L. Wadley, G. Trower, L. Backwell, F. d'Errico, Traditional glue, adhesive and poison used for composite weapons by Ju/hoan San in Nyae Nyae, Namibia. Implications for the evolution of hunting equipment in prehistory. *PLOS ONE* **10**, e0140269 (2015).

39. L. Wadley, C. Sievers, M. Bamford, P. Goldberg, F. Berna, C. Miller, Middle Stone Age bedding construction and settlement patterns at Sibudu, South Africa. *Science* **334**, 1388–1391 (2011).

40. L. Costa, H. Jimenez, R. Carvalho, J. Carvalho-Sobrinho, I. Escobar, G. Souza, Divide to conquer: Evolutionary history of Allioideae tribes (Amaryllidaceae) is linked to distinct trends of karyotype evolution. *Front. Plant Sci.* **11**, e320 (2020).

41. E. Van Jaarsveld, *Boophone* Amaryllidaceae, in *Monocotyledons*, *Illustrated Handbook of Succulent Plants*, U. Eggli, R. Nyffeler, Eds., (Berlin, Heidelberg, Springer, 2020).

42. P. Xaba, G. Duncan, *Boophone disticha*, the century plant. *Veld & Flora* **94**, 38–40 (2008).

43. G. Williamson, The remarkable *Boophone disticha* (L.f.) Herb. (Amaryllidaceae); Survivor from the northern tropical savanna woodlands to the South African semi-arid Karoo. *Cact. Succ. J.* **84**, 88–91 (2012).

44. P. Dietrich, F. Guillocheau, G. A. Douillet, N. P. Griffis, G. Baby, D. P. Le Héron, L. Barrier, M. Mathian, I. P. Montañez, C. Robin, T. Gyomlai, C. Kettler, A. Hofmann, The Glacial Paleolandscapes of Southern Africa: The legacy of the late Paleozoic ice age. *EGUsphere*, 10.5194/egusphere-2024-467. hal-04675295 (2024).

45. W. Paterson, *Quatre voyages chez les Hottentots et chez les Cafres*, *Depuis mai 1777 jusqu'en décembre 1779*. (Didot, 1790). [Four Journeys to the Hottentots and the Caffres, From May 1777 to December 1779].

46. D. Mebs, J. Rohrich, G. Kauert, P. R. Becker, Pfeilgifte: Eine toxokologische Spurensuche im Mumeum. *Dtsch. Apoth. Ztg.* **136**, 24–27 (1996). [Arrow Poisons: A Toxicological Investigation in the Museum].

47. L. Wadley, What stimulated rapid, cumulative innovation after 100,000 years ago? *J. Archaeol. Method Theory* **28**, 120–141 (2021).

48. T. Wynn, F. L. Coolidge, A Stone-Age meeting of minds: Neandertals became extinct while *Homo sapiens* prospered. A marked contrast in mental capacities may account for these different fates. *Am. Sci.* **96**, 44–51 (2008).

49. L. Wadley, Were snares and traps used in the Middle Stone Age and does it matter? A review and a case study from Sibudu, South Africa. *J. Hum. Evol.* **58**, 179–192 (2010).

50. P. Gärdenfors, M. Lombard, Causal cognition, force dynamics and early hunting technologies. *Front. Psychol.* **9**, 87 (2018).

51. G. B. Silberbauer, *Hunter and habitat in the central Kalahari Desert*. (Cambridge Univ. Press, 1981).

52. T. Rito, D. Vieira, M. Silva, E. Conde-Sousa, L. Pereira, P. Mellars, M. B. Richards, P. Soares, A dispersal of *Homo sapiens* from southern to eastern Africa immediately preceded the out-of-Africa migration. *Sci. Rep.* **9**, 4728 (2019).

53. I. Sifogeorgaki, V. Klinkenberg, I. Esteban, M. Murungi, A. S. Carr, V. B. van den Brink, G. L. Dusseldorp, New Excavations at Umhlatuzana Rockshelter, KwaZulu-Natal, South Africa: A Stratigraphic and taphonomic evaluation. *Afr. Archaeol Rev.* **37**, 551-578 (2020).

54. A. Sparrman, *A Voyage to the Cape of Good Hope*, *Towards the Antarctic Polar Circle*, *and Round the World: But Chiefly Into the Country of the Hottentots and Caffres*, *from the year 1772*, *to 1776* (Vol. 1). G. G. J. and J. Robinson, Pater-noster-row: London, (1786).

55. V. Papakosta, R. H. Smittenberg, K. Gibbs, P. Jordan, S. Isaksson, Extraction and derivatization of absorbed lipid residues from very small and very old samples of ceramic potsherds for molecular analysis by gas chromatography–mass spectrometry (GC–MS) and single compound stable carbon isotope analysis by gas chromatography–combustion–isotope ratio mass spectrometry (GC–C–IRMS). *Microchem. J.* **123**, 196–200 (2015).

56. L. Scott, H. M. Anderson, J. M. Anderson, R. M. Cowling, D. M. Richardson, S. M. Pierce, Vegetation history. *Vegetation of southern Africa* , 62–84 (1997).

57. G. J. Bredenkamp, F. Spada, E. Kazmierczak, On the origin of northern and southern hemisphere grasslands. *Plant Ecol.* **163**, 209–229 (2002).

58. M. E. Bianconi, J. Hackel, M. S. Vorontsova, A. Alberti, W. Arthan, S. V. Burke, M. R. Duvall, E. A. Kellogg, S. Lavergne, M. R. McKain, A. Meunier, C. P. Osborne, P. Traiperm, P.-A. Christin, G. Besnard, Continued adaptation of C4 photosynthesis after an initial burst of changes in the Andropogoneae grasses. *Syst. Biol.* **69**, 445–461 (2020).

59. C. A. Smith, *Common names of South African plants* (The Government Printer of South Africa: Pretoria, 1966).

60. F. Masson, XVI. An account of three journeys from the Cape Town into the southern parts of Africa; undertaken for the discovery of new plants, towards the improvement of the Royal Botanical Gardens at Kew. By Mr. Francis Masson, one of his Majesty's gardeners. Addressed to Sir John Pringle, Bart. P.R.S. *Phil. Trans. R. Soc.* **66**, 268–317 (1776).

61. L. Cheesman, J. J. Nair, J. van Staden, Antibacterial activity of crinane alkaloids from *Boophone disticha* (Amaryllidaceae). *J. Ethnopharmacol.* **140**, 405–408 (2012).

62. F. Viladomat, J. Bastida, C. Codina, J.J. Nair, W. E. Campbell, Alkaloids of the South African Amaryllidaceae in *Recent Research Developments in Phytochemistry*, S. G. Pandalai Ed., (Research Signpost Publishers, Trivandrum, 1997) pp. 131–117.

63. S. Tonisi, K. Okaiyeto, L. V. Mabinya, A. I. Okoh, Evaluation of bioactive compounds, free radical scavenging and anticancer activities of bulb extracts of *Boophone disticha* from Eastern Cape Province, South Africa. *Saudi J. Biol. Sci.* **27**, 3559–3569 (2020).

64. J. J. Nair, J. Bastida, C. Codina, F. Viladomat, J. van Staden, Alkaloids of the South African Amaryllidaceae: A review. *Nat. Prod. Commun.* **8**, 1335–1350 (2013).

65. A. S. M. Ibrakaw, Chemical investigation of some species of Amaryllidaceae from the Greater Cape Region of South Africa as a source of bioactive compounds, PhD thesis, University of the Western Cape, (2020).

66. E. E. Elgorashi, Phytochemistry and pharmacology of the family Amaryllidaceae: An overview of research at RCPGD. *Nat. Prod. Commun.* **14**, 1934578X19872929 (2019).

67. E. E. Elgorashi, S. E. Drewes, C. Morris, J. van Staden, Variation among three *Crinum* species in alkaloid content. *Biochem. Syst. Ecol.* **31**, 601–615 (2003).

68. H. Lichtenstein, *Reisen in südlichen Africa in den Jahren 1803*, *1804*, *1805 und 1806* (C. Salfeld: Berlin, 1812). [Travels in southern Africa in the years 1803, 1804, 1805, and 1806].

69. G. A. Farini, *Through the Kalahari Desert: A Narrative of a Journey with Gun*, *Camera*, *and Note-book to Lake N'Gami and back* (Vol. 32). (S. Lw, Marston, Searle, & Rivington. 1886).

70. G. W. Stow, *The Native Races of South Africa: A History of the Intrusion of the Hottentots and Bantu into the Hunting Grounds of the Bushmen*. (S. Sonnenschein & Company limited, 1905).

71. W. E. Stanford, Statement of Silayi, with reference to his life among the Bushmen. *Trans. R. Soc. S. Afr.* **1**, 435–440 (1910).

72. W. H. I. Bleek, L. Lloyd, *Specimens of Bushman folklore*. (G. Allen limited, 1911).

73. S. S. Dornan, *Pygmies and Bushmen of the Kalahari: An Account of the Hunting Tribes Inhabiting the Great Arid Plateau of the Kalahari Desert: Their Precarious Manner of Living*, *Their Habits*, *Customs and Beliefs*, *with some Reference to Bushman Art*, *both Early and of Recent Date*, *and to the Neighbouring African Tribes*. (Seeley, Service, 1925).

74. I. C. Hall, R. W. Whitehead, A pharmaco-bacteriologic study of African poisoned arrows. *J Infect Dis* **41**, 51–69 (1927).
